# An immunoinformatics study reveals a new BoLA-DR-restricted CD4+ T cell epitopes on the Gag protein of bovine leukemia virus

**DOI:** 10.1038/s41598-023-48899-4

**Published:** 2023-12-15

**Authors:** Aneta Pluta, Tasia Marie Taxis, Frank van der Meer, Sulav Shrestha, Dominic Qualley, Paul Coussens, Marzena Rola-Łuszczak, Anna Ryło, Ali Sakhawat, Saltanat Mamanova, Jacek Kuźmak

**Affiliations:** 1 Department of Biochemistry, National Veterinary Research Institute, 24-100 Puławy, Poland; 2 Department of Animal Science, College of Agriculture and Natural Resources, Michigan State University, East Lansing, MI 48824 USA; 3 Faculty of Veterinary Medicine, University of Calgary, Calgary, AB Canada; 4 Department of Chemistry and Biochemistry, and Center for One Health Studies, Berry College, Mt. Berry, GA 30149 USA; 5 Animal Quarantine Department, Ministry of National Food Security and Research, Peshawar, 25000 Pakistan; 6 Laboratory of Virology, Kazakh Scientific Research Veterinary Institute, LLP, 223 Raiymbek Avenue, 050000 Almaty, Republic of Kazakhstan

**Keywords:** Computational biology and bioinformatics, Pathogens, Virology

## Abstract

Bovine leukemia virus (BLV) is the causative agent of enzootic bovine leucosis (EBL), which has been reported worldwide. The expression of viral structural proteins: surface glycoprotein (gp51) and three core proteins - p15 (matrix), p24 (capsid), and p12 (nucleocapsid) induce a strong humoral and cellular immune response at first step of infection. CD4+ T-cell activation is generally induced by bovine leukocyte antigen (BoLA) region– positive antigen-presenting cells (APC) after processing of an exogenous viral antigen. Limited data are available on the BLV epitopes from the core proteins recognized by CD4+ T-cells. Thus, immunoinformatic analysis of Gag sequences obtained from 125 BLV isolates from Poland, Canada, Pakistan, Kazakhstan, Moldova and United States was performed to identify the presence of BoLA-DRB3 restricted CD4+ T-cell epitopes. The 379 15-mer overlapping peptides spanning the entire Gag sequence were run in BoLA-DRB3 allele-binding regions using a BoLA-DRB- peptide binding affinity prediction algorithm. The analysis identified 22 CD4+ T-cell peptide epitopes of variable length ranging from 17 to 22 amino acids. The predicted epitopes interacted with 73 different BoLA-DRB3 alleles found in BLV-infected cattle. Importantly, two epitopes were found to be linked with high proviral load in PBMC. A majority of dominant and subdominant epitopes showed high conservation across different viral strains, and therefore could be attractive targets for vaccine development.

## Introduction

Bovine leukemia virus (BLV) is the etiological agent of enzootic bovine leucosis (EBL), a chronic, lymphoproliferative disease associated with persistent lymphocytosis and B-cell lymphomas^1^. BLV, together with human T-cell leukemia viruses type 1 and 2 (HTLV-1, HTLV-2), belong to the genus *Deltaretrovirus* of the family *Retroviridae*. BLV infection has a worldwide distribution and causes substantial economic losses in the livestock industry^2,3^, infection with this virus result in a negative effect on dairy production and cow longevity, which is very likely based on the resulting impaired immune function following infection^3–5^.

The complex BLV genome encodes structural genes (*env*, *gag*, *pol*/*pro*) and nonstructural, regulatory genes (*tax*, *rex*). The *env* gene gives rise to two glycoproteins: extracellular surface subunit (SU, gp51) implicated in receptor recognition and virion attachment, and transmembrane subunit (TM, gp30) responsible for anchoring the SU-TM complex into lipid bilayers (reviewed in^6^).

The *gag* gene encodes the internal structural polyprotein- Gag (group-specific antigen), responsible for initiating the process of virion budding from the infected cell and RNA packaging in the viral particle formation process^7,8^. During viral maturation, the precursor Gag is processed into three separate proteins: matrix (MA, p15), capsid (CA, p24) and nucleocapsid (NC, p12), which undergo substantial conformational rearrangements—and confer infectivity to virions^9^. The *tax* gene encodes Tax protein, involved in activation of transcription of viral mRNA.

Both humoral and cell-mediated immune responses strongly limits BLV replicative cycle in cows naturally infected with the virus^10–14^. With respect to cellular immunity, CD4+ T-cell response to BLV, mediated through recognition of short viral peptides presented by bovine leukocyte antigen (BoLA) class II molecules on the surface of antigen-presenting cells (APC), is required for multiple anti-viral processes. These anti-viral processes confer protection from progression to persistent lymphocytosis and tumor development during BLV infection^15,16^. The CD4+ T-cell response can be directed toward any virion protein; yet, many studies into T cell immunity to BLV have focused on the external glycoprotein gp51. Indeed, existing molecular studies on CD4+ T-cells and BLV are limited to the gp51 CD4+ T-cell epitopes: peptide 98–117, peptide 169–188, and peptide 177–192^17^; peptide 51–70 and peptide 61–80^18^; peptide 98–117^19^. Recently, CD4+ T-cell epitopes on gp30 protein of the *env* gene were mapped (peptide gp30N5, peptide gp30N6 and peptide gp30N7)^19^. Furthermore, peptide 131–150 and peptide 111–130^20^; and peptides tax16/17, peptides tax19/20 and peptides tax 22–24^19^, were recognized as T-cell epitopes for Tax. To date, there are no structurally defined CD4+ T-cell epitopes from the internal gag proteins–MA (p15) and NC (p12). Experimental data regarding Gag polypeptide are limited to CA (p24). An in vitro study based on lymphocyte proliferation assays two non-universal CD4+ T-cell epitopes: peptide 31–55 and peptide 141–165 were defined^13^.

Resistance or susceptibility to progression of BLV infection in cattle was linked to the polymorphism in the BoLA class II gene^21,22^. Cattle express only two BoLA class II proteins, DR and DQ^23^. The only source of diversity in DR molecules is from the polymorphic DRB chain^24^. So far, 384 BoLA-DRB3 alleles have been identified according to the BoLA Nomenclature Committee of IPD-MHC Database [Available from: https://www.ebi.ac.uk/ipd/mhc/group/BoLA/species/]. Polymorphisms in the BoLA-DRB3 gene can influence immune response by peptide binding, antigen presentation, and T-cell receptor (TCR) gene sequences in a T-cell population and group of cytokines that mediate and regulate immunity^25,26^. Furthermore, the affinity of BLV epitopes to bind to certain BoLA-DRB3 molecules was suggested to correlate with the capacity to induce T-cell proliferation^27,28^. However, the BLV epitope peptides responsible for this binding remains unknown.

The aim of this study was to determine epitopes on Gag-derived proteins (MA, CA and NC) binding to different BoLA-DRB3 alleles and link them with blood proviral load (PVL) levels of BLV-infected cattle.

## Results

### BoLA-DRB3 allele genotyping

Out of 125 samples, 113 identified two alleles, 11 identified one allele, and genotyping failed in one sample. A total of 73 different BoLA-DRB3 alleles were identified (Table 1). Alleles with the highest frequencies were DRB3*01:01, *11:01, *10:01 and *15:01 with respective percentage values of 15.7%, 8.4%, 8.0% and 8.0%. Thirteen alleles (^*^12:01, ^*^14:01:01, ^*^27:03, ^*^07:01, ^*^18:01, ^*^41:01, ^*^105:02, ^*^116:01, ^*^160:01, ^*^09:02, ^*^24:33, ^*^25:01:01 and ^*^57:02) had frequencies ranging from 1.7% to 3.8%; 20 alleles (DRB3^*^09:01,^*^44:01, ^*^107:01, ^*^107:04, ^*^130:01, ^*^134:01,^*^139:01, ^*^142:01, ^*^02:01, ^*^04:01, ^*^05:02, ^*^05:03, ^*^08:01, ^*^09:04, ^*^13:01, ^*^15:04, ^*^17:01, ^*^24:03, ^*^31:01 and ^*^20:01:01) had frequencies ranging from 0.8% to 1.3%; and the remaining 36 alleles had frequencies below 0.5% (Table 1).

**Table 1:** Characterization of BLV-infected cattle in this study.

No.	Cow ID	Country of origin/region/farm	Breed	BoLA-DRB3 genotype		BLV isolate ID	BLV proviral copy number per 100,000 cells	Gag gene GenBank Accession Number
1	A_274	Canada/Alberta/A	Holstein	010:01	011:01	1Can	34.57	OP146577
2	A_395	Canada/Alberta/A	Holstein	010:01	011:01	2Can	25.71	OP146578
3	A_2613	Canada/Alberta/A	Holstein	001:01	107:01:00	3Can	6.88	OP146579
4	B_537	Canada/Alberta/B	Holstein–Friesian	015:01	001:01	4Can	16.68	OP146580
5	B_671	Canada/Alberta/B	Holstein–Friesian	015:01	011:01	5Can	15.86	OP146581
6	B_700	Canada/Alberta/B	Jersey	015:01	044:01	7Can	23.35	OP146583
7	B_705	Canada/Alberta/B	Montbeliarde	015:01	005:03	8Can	15.14	OP146584
8	B_759	Canada/Alberta/B	Holstein–Friesian	027:03	010:01	9Can	103.71	OP146585
9	B_873	Canada/Alberta/B	Montbeliarde	005:08	024:33	10Can	117.38	OP146586
10	C_3282	Canada/Alberta/C	Holstein	015:01	010:01	11Can	26.02	OP146587
11	C_3284	Canada/Alberta/C	Holstein	015:01	015:01	12Can	70.83	OP146588
12	C_3326	Canada/Alberta/C	Holstein	015:01	015:01	13Can	136.68	OP146589
13	C_3394	Canada/Alberta/C	Holstein	105:02:00	001:01	14Can	26.67	OP146590
14	C_10946	Canada/Alberta/C	Holstein	012:01	114:01:00	15Can	82.56	OP146591
15	C_10963	Canada/Alberta/C	Holstein	010:01	011:01	16Can	59.12	OP146592
16	C_18672	Canada/Alberta/C	Holstein	015:01	010:01	17Can	71.87	OP146593
17	C_18735	Canada/Alberta/C	Holstein	010:01	15:01	18Can	41.35	OP146594
18	D_6	Canada/Alberta/D	Holstein	001:01	112:02:00	19Can	93.64	OP146595
19	D_24	Canada/Alberta/D	Holstein	015:01	007:01	20Can	45.74	OP146596
20	D_45	Canada/Alberta/D	Holstein	015:01	011:01	21Can	61.66	OP146597
21	D_61	Canada/Alberta/D	Holstein	024:15	027:18	22Can	169.24	OP146598
22	D_81	Canada/Alberta/D	Holstein	015:01	012:01	23Can	59.01	OP146599
23	D_90	Canada/Alberta/D	Holstein	105:02:00	001:01	24Can	14.17	OP146600
24	D_148	Canada/Alberta/D	Holstein	105:02:00	001:01	25Can	174.19	OP146601
25	L368	Pakistan/Punjab/B	Friesian (cross)	080:01	139:01	1Pak	565.44	OP146492
26	L376	Pakistan/Punjab/B	Friesian (cross)	116:01:00	015:01	2Pak	631.50	OP146493
27	L391Q	Pakistan/Punjab/B	Friesian (cross)	057:02	043:03	3Pak	266.17	OP146494
28	L392	Pakistan/Punjab/B	Friesian (cross)	057:02	005:02	4Pak	103.59	OP146495
29	L348	Pakistan/Punjab/B	Friesian (cross)	057:02	107:01:00	5Pak	40.76	OP146496
30	L364	Pakistan/Punjab/B	Friesian (cross)	057:02	005:02	6Pak	267.93	OP146497
31	L367	Pakistan/Punjab/B	Friesian (cross)	018:01	086:03	7Pak	149.21	OP146498
32	L371	Pakistan/Punjab/B	Friesian (cross)	001:01	024:33	8Pak	88.86	OP146499
33	L382	Pakistan/Punjab/B	Friesian (cross)	009:02	107:04:00	9Pak	210.03	OP146500
34	P474	Pakistan/ Khyber Pakhtunkhwa/H	Friesian	012:01	010:01	10Pak	826.02	OP146501
35	P496	Pakistan/ Khyber Pakhtunkhwa/H	Friesian	010:01	081:01	11Pak	832.73	OP146502
36	P506	Pakistan/ Khyber Pakhtunkhwa/H	Jersey	008:01	011:01	12Pak	953.91	OP146503
37	P2	Pakistan/ Khyber Pakhtunkhwa/H	Friesian	010:01	010:01	14Pak	1,046.14	OP146504
38	P5	Pakistan/ Khyber Pakhtunkhwa/H	Friesian	011:01	015:04	15Pak	11.31	OP146505
39	P6	Pakistan/ Khyber Pakhtunkhwa/H	Jersey	025:01:01	044:01	17Pak	2,011.91	OP146507
40	P29	Pakistan/ Khyber Pakhtunkhwa/H	Jersey	002:01	020:01:01	18Pak	838.08	OP146508
41	P30	Pakistan/ Khyber Pakhtunkhwa/H	Jersey	086:02	044:01	19Pak	205.90	OP146509
42	P479	Pakistan/ Khyber Pakhtunkhwa/H	Jersey	025:01:01	018:01	21Pak	144.01	OP146510
43	P488	Pakistan/ Khyber Pakhtunkhwa/H	Friesian	001:01	nd	22Pak	881.88	OP146511
44	P492	Pakistan/ Khyber Pakhtunkhwa/H	Friesian	001:01	024:33	23Pak	38.73	OP146512
45	8MD	Moldova/Region Riscani/A	Black-Motley	012:01	nd	1M	7.91	OP146513
46	3MD	Moldova/Region Riscani/A	Black-Motley	020:01:01	028:01	2M	0.07	OP146514
47	6MD	Moldova/Region Riscani/A	Black-Motley	018:01	105:02:00	3M	12.38	OP146515
48	1MD	Moldova/Region Riscani/A	Black-Motley	005:04	019:02	4M	410.39	OP146516
49	7MD	Moldova/Region Riscani/A	Black-Motley	008:01	011:02	5M	0.92	OP146517
50	13MD	Moldova/Region Hincesti/B	Black-Motley	002:01	010:01	6M	15.67	OP146518
51	15MD	Moldova/Region Hincesti/B	Black-Motley	012:01	010:01	7M	31.33	OP146519
52	19MD	Moldova/Region Hincesti/B	Black-Motley	001:01	010:01	8M	29.07	OP146520
53	20MD	Moldova/Region Hincesti/B	Black-Motley	001:01	015:05	9M	0.01	OP146521
54	16MD	Moldova/Region Hincesti/B	Black-Motley	014:01:01	001:01	10M	5.57	OP146522
55	14MD	Moldova/Region Hincesti/B	Black-Motley	014:03	075:03	11M	0.06	OP146523
56	17MD	Moldova/Region Hincesti/B	Black-Motley	001:01	nd	12M	0.03	OP146524
57	9MD	Moldova/Region Anenii Noi/C	Black-Motley	010:01	nd	14M	0.001	OP146525
58	11MD	Moldova/Region Anenii Noi/C	Black-Motley	007:01	024:32	16M	0.02	OP146526
59	0081Z_P	Poland/Podlaskie/A	Holstein–Friesian	011:01	001:01	1P	103.60	OP146529
60	011TL_L	Poland/Lublin/B	Holstein–Friesian	011:01	018:01	2P	0.06	OP146530
61	019L_P	Poland/Podlaskie/C	Holstein–Friesian	001:01	024:33	3P	0.05	OP146531
62	11W_W-M	Poland/ Warmian-Masurian/D	Holstein–Friesian	011:01	009:04	4P	0.02	OP146532
63	10Sz_W-M	Poland/ Warmian-Masurian/E	Holstein–Friesian	028:05	004:01	5P	157.45	OP146533
64	0405W_W-M	Poland/ Warmian-Masurian/F	Holstein–Friesian	018:01	025:01:01	6P	14.84	OP146534
65	0253G_W-M	Poland/ Warmian-Masurian/G	Holstein–Friesian	001:01	001:01	7P	2.42	OP146535
66	0183S_P	Poland/Podlaskie/H	Holstein–Friesian	009:02	009:02	8P	0.05	OP146536
67	297WS_M	Poland/Masovian/I	Holstein–Friesian	010:01	035:01	12P	36.62	OP146538
68	0741M_S	Poland/Silesian/J	Holstein–Friesian	011:01	001:01	14P	137.50	OP146539
69	0742M_S	Poland/Silesian/J	Holstein–Friesian	027:03	027:03	15P	20.894	OP146540
70	0138O_L-S	Poland/Lower Silesian/K	Holstein–Friesian	038:01	nd	16P	142.06	OP146541
71	030O_W-M	Poland/ Warmian-Masurian/L	Holstein–Friesian	015:01	025:01:01	17P	81.33	OP146542
72	0132O_L-S	Poland/Lower Silesian/K	Holstein–Friesian	009:01	012:01	18P	38.29	OP146543
73	0136O_L-S	Poland/Lower Silesian/K	Holstein–Friesian	012:03	142:01:00	19P	308.69	OP146544
74	0184S_P	Poland/Podlaskie/H	Holstein–Friesian	014:01:01	014:01:01	20P	144.93	OP146545
75	01310O_L-S	Poland/Lower Silesian/K	Holstein–Friesian	024:03	nd	23P	0.03	OP146547
76	0134O_L-S	Poland/Lower Silesian/K	Holstein–Friesian	014:01:01	014:04	24P	1.20	OP146548
77	03510M_P	Poland/Podlaskie/P	Holstein–Friesian	nd	nd	25P	42,21	OP146549
78	020B_S	Poland/Silesian/M	Holstein–Friesian	016:01	010:01	27P	0.34	OP146550
79	019WM_P	Poland/Podlaskie/N	Holstein–Friesian	011:01	009:04	28P	0.02	OP146551
80	022(1A)AGD_K-P	Poland/Kuyavian-Pomeranian/O	Holstein–Friesian	006:01	141:01:00	30P	0.97	OP146552
81	14D_BKO	East Kazakhstan/Altai/DO	Local, without breed	013:01	130:01:00	1K	18.13	OP146469
82	15D_BKO	East Kazakhstan/Altai/DO	Local, without breed	013:01	015:04	2K	35.33	OP146470
83	11be_BKO	East Kazakhstan/Altai/BO	Local, without breed	017:01	009:01	4K	273.20	OP146490
84	17D_BKO	East Kazakhstan/Altai/DO	Alatau	017:01	009:01	5K	241.93	OP146491
85	32K_CKO	North Kazakhstan/Gabit Musirepov/MU	Black-Motley	027:03	139:01	7K	606.84	OP146471
86	8D_BKO	East Kazakhstan/Altai/DO	Alatau	009:02	107:04:00	9K	102.45	OP146473
87	3D_BKO	East Kazakhstan/Altai/DO	Alatau	041:01	157:01:00	10K	367.70	OP146474
88	2be_BKO	East Kazakhstan/Altai/BO	Local, without breed	041:01	nd	11K	284.45	OP146475
89	13be_BKO	East Kazakhstan/Altai/BO	Local, without breed	041:01	041:01	12K	273.16	OP146476
90	466P_CKO	North Kazakhstan/Gabit Musirepov/MU	Black-Motley	001:01	nd	14K	475.26	OP146477
91	17be_BKO	East Kazakhstan/Altai/BO	Local, without breed	041:01	nd	15K	364.70	OP146478
92	K1	North Kazakhstan/K	Black-Motley	007:01	011:01	17K	0.88	OP146479
93	K13	North Kazakhstan/K	Black-Motley	032:01	005:07	18K	267.71	OP146480
94	K7	North Kazakhstan/K	Black-Motley	027:13	007:01	19K	10.95	OP146481
95	K10	North Kazakhstan/K	Black-Motley	027:03	024:03	20K	5.20	OP146482
96	10_ALM	Kazakhstan/Almaty/A	Santa Gertrudis	003:01:01	004:01	22K	33.14	OP146483
97	42_ALM	Kazakhstan/Almaty/E	nd	045:01	134:01	23K	0.002	OP146484
98	44_ALM	Kazakhstan/Almaty/E	nd	001:01	007:01	24K	0.002	OP146489
99	3Z_ALM	Kazakhstan/Almaty/A	Santa Gertrudis	011:01	116:01:00	3Ż	0.41	OP146485
100	4Z_ALM	Kazakhstan/Almaty/A	Local, without breed	001:01	112:03:00	4Ż/2	481.84	OP146486
101	11UKR_BKO	East Kazakhstan/Ulansky/U	Simental	005:03	134:01:00	11UKR	0.06	OP146488
102	A_1652	USA/Michigan/A	Holstein	001:01	010:01	10USA	16.89	OP146553
103	A_1682	USA/Michigan/A	Holstein	011:01	010:03	12USA	9.84	OP146554
104	A_1748	USA/Michigan/A	Holstein	010:01	010:04	13USA	12.74	OP146555
105	A_1762	USA/Michigan/A	Holstein	011:01	116:01:00	15USA	18.16	OP146556
106	B_5031	USA/Michigan/B	Holstein	001:01	160:01:00	16USA	19.43	OP146557
107	B_5241	USA/Michigan/B	Holstein	001:01	160:01:00	17USA	15.09	OP146558
108	B_5277	USA/Michigan/B	Holstein	001:01	014:01:01	18USA	15.08	OP146559
109	A_479	USA/Michigan/A	Holstein	011:01	116:01:00	1USA	12.96	OP146560
110	B_5322	USA/Michigan/B	Holstein	011:01	015:01	20USA	32.61	OP146561
111	B_5365	USA/Michigan/B	Holstein	015:01	015:01	24USA	25.84	OP146562
112	B_5389	USA/Michigan/B	Holstein	001:01	130:01:00	26USA	28.64	OP146563
113	B_5393	USA/Michigan/B	Holstein	001:01	001:01	27USA	16.62	OP146564
114	B_5492	USA/Michigan/B	Holstein	001:01	011:01	28USA	25.87	OP146565
115	B_5499	USA/Michigan/B	Holstein	001:01	011:01	29USA	16.55	OP146566
116	A_1236	USA/Michigan/A	Holstein	001:01	160:01:00	2USA	0.31	OP146567
117	B_5503	USA/Michigan/B	Holstein	031:01	089:01	30USA	21.87	OP146568
118	B_5536	USA/Michigan/B	Holstein	001:01	160:01:00	31USA	13.49	OP146569
119	B_86101	USA/Michigan/B	Holstein	012:01	012:01	33USA	11.55	OP146570
120	A_1455	USA/Michigan/A	Holstein	001:01	014:01:01	3USA	0.77	OP146571
121	A_1540	USA/Michigan/A	Holstein	012:01	142:01:00	4USA	46.92	OP146572
122	A_1553	USA/Michigan/A	Holstein	001:01	031:01	5USA	0.67	OP146573
123	A_1568	USA/Michigan/A	Holstein	001:01	nd	6USA	16.46	OP146574
124	A_1600	USA/Michigan/A	Holstein	001:01	027:03	7USA	6.73	OP146575
125	A_1605	USA/Michigan/A	Holstein	001:01	nd	8USA	15.19	OP146576

### Analysis of amino acid sequence variability of the Gag protein

The Shannon entropy (Hx) plot exhibited 49 peaks with values ranging from 0.05 to 1.13 (Supplementary Fig. S1, Supplementary Table S1). Considering the three Gag domains, the highest total entropy (6.33) occurred in the matrix (MA). For the capsid (CA) and nucleocapsid (NC) domains, the total entropy was 4.09 and 1.38, respectively. A detailed multiple sequence alignment analysis on 395 amino acid sites indicated 345 as conserved and 49 with non-synonymous single nucleotide polymorphisms (nsSNPs). A substantial number of nsSNPs found in the MA and CA domains suggested the possibility of positive selection on variable sites of the protein (Supplementary Fig. S2). The dN/dS ratios were drawn over the midpoint window position (window length 9, step size 3) from the whole coding region. The following regions with putative positive selection sites were identified: 133–149 nt, 175–195 nt, 199–216 nt, 250–279 nt, 316–342 nt in MA, 829–843 nt, 952–972 nt in CA and 1087–1107 nt for NC domains, respectively. Thirteen codons located in these regions had dN/dS ratios > 1 that identified them as major sites for the occurrence of positive selection. These were codons 48, 61, 63, 69, 87, 88, 108, 109, 112 in MA domain; 278, 318, 323 in CA; and 365 in NC (Supplementary Table S2 and Supplementary Fig. S1).

### Determination of epitope peptides in the Gag protein consensus sequence based on BoLA-DRB3

In order to search for common epitope peptides on Gag protein, we determined the consensus sequence from 125 sequences described in this study. To detect putative binding sites for BoLA-DRB3, we used the 379 15-mer overlapping peptides that spanned the entire Gag consensus sequence in 73 BoLA-DRB3 allele binding regions in NetBoLAIIpan (pan-specific predictor for BoLA-DRB3 Ag presentation). Analysis revealed 22 putative regions within Gag proteins with high binding affinity to BoLA-DRB3 alleles. The binding affinities of the epitopes and complete calculations are presented in Supplementary Table S3.

**Figure 1:**
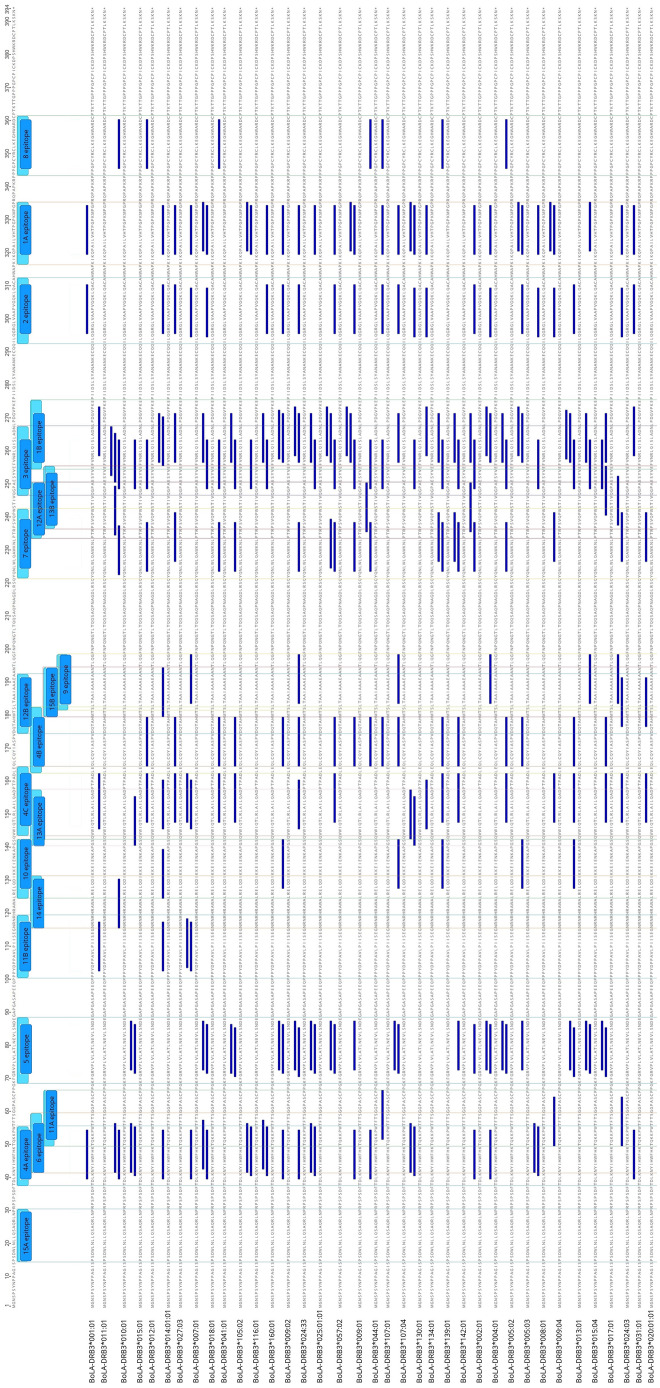
Distribution of the BoLA-DRB3-restricted CD4+ T-cell epitopes along the Gag polyprotein. The labeled blue bars in the upper part of figure refer to the identified 22 epitopes 1A–15B. The figure shows the localization of the epitopes for the most commonly detected BoLA-DRB3 (on the left side of the figure). The distribution of the epitopes for the all analysed alleles is shown in Supplementary Fig. S3.

Detailed analysis indicated that the highest number of BoLA-DRB3 alleles, 44 out of 73 (60.3%), had significant binding affinity to peptide 320#QPAILVHTPGPKMPG and additionally to 5 overlapping peptides sharing the same ILVHTPGPK core sequence (as shown in Table 2). A combined sequence 317-KIKQPAILVHTPGPKMPGPR-336, formed by the group of these peptides, was designated epitope 1A (as shown in Fig. 1 and Supplementary Fig. S3). The same number of BoLA-DRB3 alleles—44 out of 73 (60.3%)—with high binding affinity were determined for peptide 257#VNRLQISLADNLPDG and to a lesser extent, for 6 overlapping peptides sharing the common core sequence LQISLADNL. Summary sequence 255-EFVNRLQISLADNLPDGVPKE-275 for a group of the peptides was designated epitope 1B. The third most frequent binding alleles, 42 out of 73 (57.5%), were peptide 296#GRGLVAAPVGQKLQA and 5 other contiguous peptides with the common core sequence LVAAPVGQK. The completed sequence 293-ILQGRGLVAAPVGQKLQACA-312 for a group of these peptides was designated epitope 2. The fourth peptide with significant binding affinity for multiple alleles, 38 out of 73 (52.1%), was peptide 249#PAESYVEFVNRLQIS and 6 other contiguous peptides. Deduced sequence 247-QGPAESYVEFVNRLQISLADN-267, based on those peptides with common core sequence VEFVNRLQI, was designated as epitope 3. Additionally, a large number of BoLA-DRB3 alleles, 32 out of 73 (43.8%), had significant binding affinity to the 40#LKNYIHWFHKTQKKP, 165#QLCQYIASPVDQTAH and 147/148#QTLRLAILQADPTPAD main peptides. Summary sequences 38-TDLKNYIHWFHKTQKKPW-55, 163-LEQLCQYIASPVDQTAH MTS-182 and 144-VWIQTLRLAILQADPTPADLE-164 were respectively designated epitopes 4A, 4B and 4C.

**Table 2:** CD4+ T cell epitope peptides found in BLV Gag sequences.

No.	Peptide	Position	Averaged number of BoLA-DRB3 alleles that bind to a group of peptides with the same core sequence (%)	Number of BoLA-DRB3 alleles bound to the peptide (%)	Core sequence	Name of epitope
1	KIKQPAILVHTPGPK	317	27.3 (37.4)	9 (12.3)	ILVHTPGPK	1A
2	IKQPAILVHTPGPKM	318		22 (30.1)	ILVHTPGPK	
3	KQPAILVHTPGPKMP	319		39 (53.4)	ILVHTPGPK	
4	**QPAILVHTPGPKMPG**	**320**		**44 (60.3)**	**ILVHTPGPK**	
5	PAILVHTPGPKMPGP	321		43 (58.9)	ILVHTPGPK	
6	AILVHTPGPKMPGPR	322		7 (9.6)	LVHTPGPKM	
7	EFVNRLQISLADNLP	255	20.0 (27.4)	5 (6.8)	LQISLADNL	1B
8	FVNRLQISLADNLPD	256		26 (35.6)	LQISLADNL	
9	**VNRLQISLADNLPDG**	**257**		**44 (60.3)**	**LQISLADNL**	
10	NRLQISLADNLPDGV	258		33 (45.2)	ISLADNLPD	
11	RLQISLADNLPDGVP	259		26 (35.6)	ISLADNLPD	
12	LQISLADNLPDGVPK	260		5 (6.8)	ISLADNLPD	
13	QISLADNLPDGVPKE	261		1 (1.4)	ISLADNLPD	
14	ILQGRGLVAAPVGQK	293	27.5 (37.7)	18 (24.7)	LVAAPVGQK	2
15	LQGRGLVAAPVGQKL	294		32 (43.8)	LVAAPVGQK	
16	QGRGLVAAPVGQKLQ	295		41 (56.2)	LVAAPVGQK	
17	**GRGLVAAPVGQKLQA**	**296**		**42 (57.5)**	**LVAAPVGQK**	
18	RGLVAAPVGQKLQAC	297		27 (37.0)	LVAAPVGQK	
19	GLVAAPVGQKLQACA	298		5 (6.8)	LVAAPVGQK	
20	QGPAESYVEFVNRLQ	247	14.1 (19.4)	5 (6.8)	YVEFVNRLQ	3
21	GPAESYVEFVNRLQI	248		17 (23.3)	YVEFVNRLQ	
22	**PAESYVEFVNRLQIS**	**249**		**38 (52.1)**	**VEFVNRLQI**	
23	AESYVEFVNRLQISL	250		20 (27.4)	VEFVNRLQI	
24	ESYVEFVNRLQISLA	251		14 (19.2)	VEFVNRLQI	
25	SYVEFVNRLQISLAD	252		4 (5.5)	VEFVNRLQI	
26	YVEFVNRLQISLADN	253		1 (1.4)	FVNRLQISL	
27	TDLKNYIHWFHKTQK	38	23.3 (31.8)	7 (9.6)	IHWFHKTQK	4A
28	DLKNYIHWFHKTQKK	39		24 (32.9)	IHWFHKTQK	
29	**LKNYIHWFHKTQKKP**	**40**		**32 (43.8)**	**IHWFHKTQK**	
30	KNYIHWFHKTQKKPW	41		30 (41.1)	IHWFHKTQK	
31	LEQLCQYIASPVDQT	163	19.2 (26.3)	3 (4.1)	YIASPVDQT	4B
32	EQLCQYIASPVDQTA	164		25 (34.2)	YIASPVDQT	
33	**QLCQYIASPVDQTAH**	**165**		**32 (43.8)**	**YIASPVDQT**	
34	LCQYIASPVDQTAHM	166		28 (38.4)	YIASPVDQT	
35	CQYIASPVDQTAHMT	167		20 (27.4)	YIASPVDQT	
36	QYIASPVDQTAHMTS	168		7 (9.6)	YIASPVDQT	
37	VWIQTLRLAILQADP	144	16.9 (23.1)	5 (6.8)	LRLAILQAD	4C
38	WIQTLRLAILQADPT	145		8 (11.0)	LRLAILQAD	
39	IQTLRLAILQADPTP	146		24 (32.9)	LAILQADPT	
40	**QTLRLAILQADPTPA**	**147**		**32 (43.8)**	**LAILQADPT**	
41	**TLRLAILQADPTPAD**	**148**		**32 (43.8)**	**LAILQADPT**	
42	LRLAILQADPTPADL	149		15 (20.5)	ILQADPTPA	
43	RLAILQADPTPADLE	150		2 (2.7)	ILQADPTPA	
44	KFGRVPLVLATLNEV	69	15.3 (21.0)	3 (4.1)	LVLATLNEV	5
45	FGRVPLVLATLNEVL	70		6 (8.2)	LVLATLNEV	
46	GRVPLVLATLNEVLS	71		20 (27.4)	LVLATLNEV	
47	**RVPLVLATLNEVLSN**	**72**		**27 (37.0)**	**LVLATLNEV**	
48	VPLVLATLNEVLSND	73		25 (34.2)	LVLATLNEV	
49	PLVLATLNEVLSNDE	74		11 (15.1)	LATLNEVLS	
50	**NYIHWFHKTQKKPWT**	**42**	12.0 (16.4)	**24 (32.9)**	**FHKTQKKPW**	6
51	YIHWFHKTQKKPWTF	43		15 (20.5)	FHKTQKKPW	
52	IHWFHKTQKKPWTFT	44		5 (6.8)	FHKTQKKPW	
53	HWFHKTQKKPWTFTS	45		4 (5.5)	FHKTQKKPW	
54	YQNLWLQAWKNLPTR	222	15.7 (21.5)	5 (6.8)	LQAWKNLPT	7
55	QNLWLQAWKNLPTRP	223		11 (15.1)	LQAWKNLPT	
56	NLWLQAWKNLPTRPS	224		21 (28.8)	LQAWKNLPT	
57	**LWLQAWKNLPTRPSV**	**225**		**23 (31.5)**	**WKNLPTRPS**	
58	WLQAWKNLPTRPSVQ	226		17 (23.3)	WKNLPTRPS	
59	LQAWKNLPTRPSVQP	227		19 (26.0)	WKNLPTRPS	
60	QAWKNLPTRPSVQPW	228		14 (19.2)	WKNLPTRPS	
61	PPGPCYRCLKEGHWA	344	8.0 (11.0)	4 (5.5)	YRCLKEGHW	8
62	PGPCYRCLKEGHWAR	345		10 (13.7)	YRCLKEGHW	
63	**GPCYRCLKEGHWARD**	**346**		**16 (21.9)**	**YRCLKEGHW**	
64	PCYRCLKEGHWARDC	347		2 (2.7)	YRCLKEGHW	
65	SLTAAIAAAEAANTL	182	7.7 (10.5)	1 (1.4)	IAAAEAANT	9
66	LTAAIAAAEAANTLQ	183		9 (12.3)	IAAAEAANT	
67	**TAAIAAAEAANTLQG**	**184**		**13 (17.8)**	**IAAAEAANT**	
68	LRELQDIKKEIENKA	125	7.7 (10.5)	1 (1.4)	LQDIKKEIE	10
69	ELQDIKKEIENKAPG	127		10 (13.7)	IKKEIENKA	
70	**LQDIKKEIENKAPGS**	**128**		**12 (16.4)**	**IKKEIENKA**	
71	TQKKPWTFTSGGPAS	50	5.0 (6.8)	5 (6.8)	WTFTSGGPA	11A
72	QKKPWTFTSGGPASC	51		3 (4.1)	WTFTSGGPA	
73	**KKPWTFTSGGPASCP**	**52**		**7 (9.6)**	**WTFTSGGPA**	
74	PPYDPPAVLPIISEG	101	4.6 (6.3)	2 (2.7)	YDPPAVLPI	11B
75	PYDPPAVLPIISEGN	102		5 (6.8)	PAVLPIISE	
76	**YDPPAVLPIISEGNR**	**103**		**7 (9.6)**	**PAVLPIISE**	
77	DPPAVLPIISEGNRN	104		6 (8.2)	VLPIISEGN	
78	PPAVLPIISEGNRNR	105		3 (4.1)	VLPIISEGN	
79	PTRPSVQPWSTIVQG	234	4.7 (6.4)	2 (2.7)	VQPWSTIVQ	12A
80	**TRPSVQPWSTIVQGP**	**235**		**6 (8.2)**	**VQPWSTIVQ**	
81	**RPSVQPWSTIVQGPA**	**236**		**6 (8.2)**	**VQPWSTIVQ**	
82	DQTAHMTSLTAAIAA	175	3.0 (4.1)	1 (1.4)	MTSLTAAIA	12B
83	QTAHMTSLTAAIAAA	176		4 (5.5)	MTSLTAAIA	
84	**TAHMTSLTAAIAAAE**	**177**		**6 (8.2)**	**MTSLTAAIA**	
85	AHMTSLTAAIAAAEA	178		1 (1.4)	LTAAIAAAE	
86	**GSQVWIQTLRLAILQ**	**141**	2.3 (3.2)	**3 (4.1)**	**WIQTLRLAI**	13A
87	SQVWIQTLRLAILQA	142		2 (2.7)	IQTLRLAIL	
88	QVWIQTLRLAILQAD	143		2 (2.7)	IQTLRLAIL	
89	PSVQPWSTIVQGPAE	237	2.3 (3.1)	2 (2.7)	WSTIVQGPA	13B
90	**SVQPWSTIVQGPAES**	**238**		**3 (4.1)**	**WSTIVQGPA**	
91	**VQPWSTIVQGPAESY**	**239**		**3 (4.1)**	**WSTIVQGPA**	
92	PWSTIVQGPAESYVE	241		1 (1.4)	IVQGPAESY	
93	**NRNRHRAWALRELQD**	**116**	1.5 (2.1)	**2 (2.7)**	**HRAWALREL**	14
94	RNRHRAWALRELQDI	117		1 (1.4)	HRAWALREL	
95	**PSDWLNLLQSAQRLN**	**15**	1.0 (1.4)	**1 (1.4)**	**LNLLQSAQR**	15A
96	**SDWLNLLQSAQRLNP**	**16**		**1 (1.4)**	**LNLLQSAQR**	
97	**MTSLTAAIAAAEAAN**	**180**	1.0 (1.4)	**1 (1.4)**	**LTAAIAAAE**	15B

In bold type are marked the peptide sequences showing the most frequent affinity for BoLA-DRB3 alleles in particular Gag regions.

The eighth peptide frequently bound by BoLA-DRB3 alleles, 27 out of 73 (37.0%), was sequence 72#RVPLVLATLNEVLSN plus 5 adjacent peptides with common core sequence LVLATLNEV. Completed sequence 69-KFGRVPLVLATLNEVLSNDE-88 for the group of peptides was designated epitope 5. The ninth peptide 42#NYIHWFHKTQKKPWT, for which 24 of 73 (32.9%) different alleles showed binding affinity together with 3 overlapping peptides with common core sequence FHKTQKKPW, created sequence 42-NYIHWFHKTQKKPWTFTS-59, which was designated epitope 6. The tenth peptide 225#LWLQAWKNLPTRPSV, to which 23 of 73 (31.5%) BoLA-DRB3 alleles had binding affinity together with the other 6 peptides with common core sequence WKNLPTRPS, represented the combined sequence 222-YQNLWLQAWKNLPTRPSVQPW-242 designated as epitope 7. The eleventh peptide 346#GPCYRCLKEGHWARD showing binding affinity for 16 of 73 alleles (21.9%), together with three adjacent peptides with YRCLKEGHW core sequence, presented summarized sequence 344-PPGPCYRCLKEGHWARDC-361 and was designated epitope 8 (Table 2, Fig. 1). The remaining eleven peptides (184#TAAIAAAEAANTLQG, 128#LQDIKKEIENKAPGS, 52#KKPWTFTSGGPASCP, 103#YDPPAVLPIISEGNR, 235/236#TRPSVQPWSTIVQGPA, 177#TAHMTSLTAAIAAAE, 141#GSQVWIQTLRLAILQ, 238/239#SVQPWSTIVQGPAESY, 116#NRNRHRAWALRELQD, 15/16#PSDWLNLLQSAQRLNP and 180#MTSLTAAIAAAEAAN) formed epitopes respectively named 9-10 (183-SLTAAIAAAEAANTLQG-199, 126-LRELQDIKKEIENKAPGS-143), 11A-11B (50-T QKKPWTFTSGGPASCP-66, 102-PPYDPPAVLPIISEGNRNR-120), 12A-12B (235-PTRPSVQPWSTIVQGPA-251, 176-DQTAHMTSLTAAIAAAE-192), 13A-13B (142-GSQVW IQTLRLAILQAD-158, 238-PSVQPWSTIVQGPAESYVE-256), 14 (117-NRNRHRAWALRELQDI-132) and 15A-15B (15-PSDWLNLLQSAQRLNP-30, 181-MTSLTAAIAAAEAAN-195) were subdominant. These epitopes characterized a high BoLA-DRB3 binding specificity and were consequently dedicated to a small group of alleles (not exceeding 18% of all alleles in this study) (Table 2, Supplementary Fig. S3).

**Figure 2:**
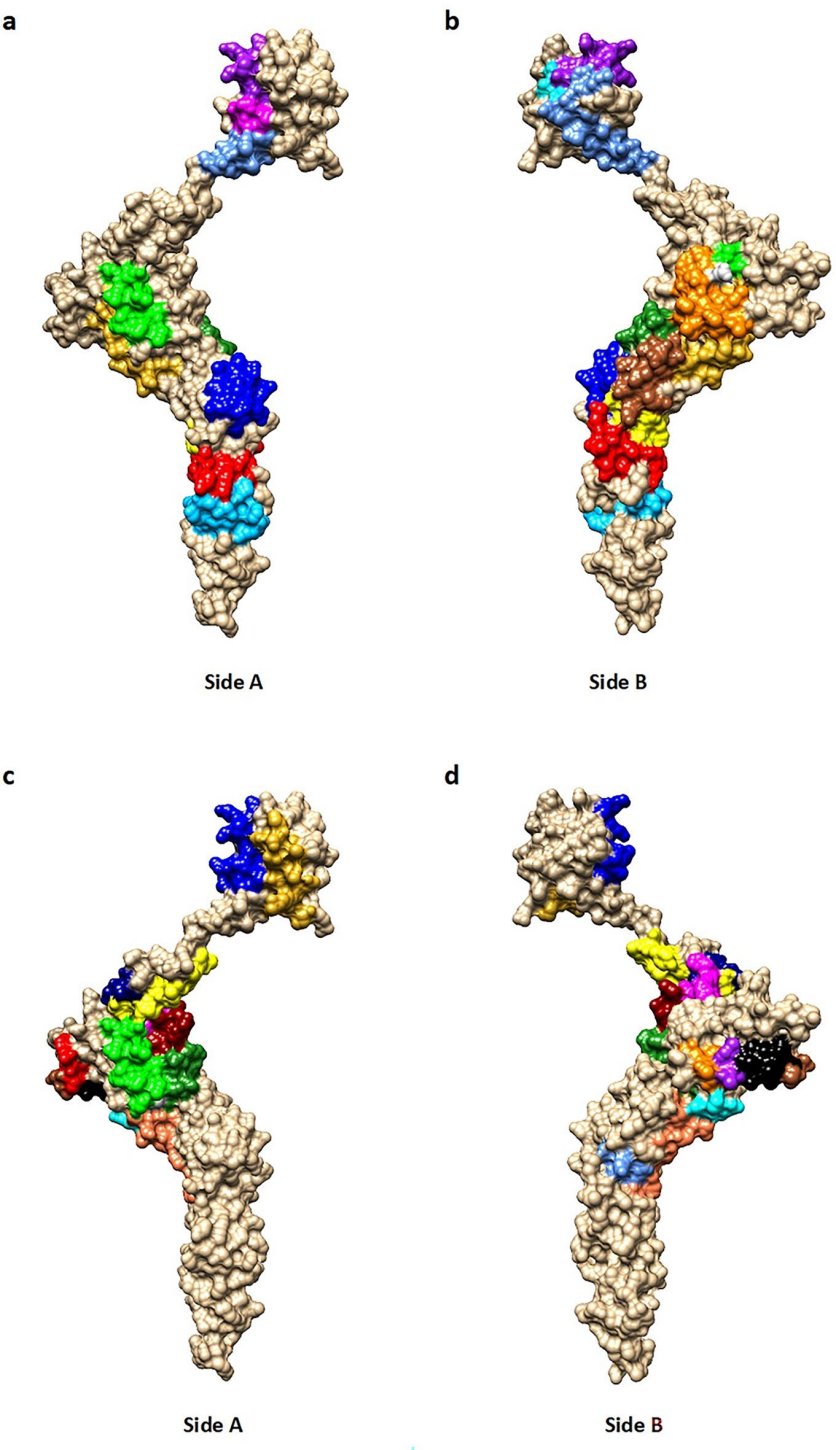
(**a**–**d**) Representation of the dominant and subdominant CD4+ T cell epitopes on 3D protein structure model of BLV Gag. The model is shown as space-filled images of opposite sides arbitrarily named side A and side B. (A–B) Gag structure contain 11 dominant epitopes: 1A (red), 1B (forest green), 2 (blue), 3 (yellow), 4A (cyan), 4B (orange), 4C (green), 5 (cornflower blue), 6 (magenta), 7 (goldenrod), 8 (sky blue), 1B + 3 [255–267] (brown), 4A + 6 [42–55] (purple), 4B + 4C (163–164): silver. (C–D) Gag structure contain 11 subdominant epitopes: 9 (red), 10 (forest green), 11A (blue), 11B (yellow), 12A (cyan), 12B (orange), 13A (green), 13B (cornflower blue), 14 (magenta), 15A (goldenrod), 15B (all residues overlap with one or more other sequences), 9 + 15B [192–194] (brown), 12B + 15B [180–181] (purple), 10 + 13A [141–142] (silver), 11B + 14 [116–119] (navy blue), 12A + 13B [237–250] (coral), 10 + 14 [125–131] (dark red), 9 + 12B + 15B [182–191] (black).

A total of 22 CD4+ T-cell epitopes were identified. Out of 22, five epitopes (15A, 4A, 6, 11A and 5) were located in the matrix; one epitope (11B) within the matrix-capsid; 14 epitopes (14, 10, 13C, 4C, 4B, 12B, 15B, 9, 7, 12A, 13B, 3, 1B and 2) in the capsid; one epitope (1A) at the capsid-nucleocapsid; and one epitope in the nucleocapsid domain (8) (Supplementary Fig. S2). The majority of these epitopes could be considered vaccine candidate antigens as they are exposed on the surface (a topological approach was presented in Fig. 2A–D).

### Relation between the incidence of BoLA-DRB3 alleles and number of CD4+ T-cell epitopes

Predictions of BoLA-DRB3 peptide’s binding affinity were performed for 73 alleles determined in BLV-infected cattle tested in this study. The results shown in Supplementary Table S4 indicate that the examined alleles had significant binding specificity to the epitope peptides. BoLA-DRB3 molecules were found to interact with binding core sequences of 3–11 CD4+ T-cell epitopes of Gag protein. Notably, the first ten BoLA-BRB3 alleles distinguishable by binding the lowest number of epitopes were distributed as follows: *01:01, *11:01, *11:02, *20:01:01 and *112:02 had affinity to three epitopes; *116:01, *134:01, *31:01, *114:01 and *14:04 had affinity to four epitopes (Supplementary Table S4). The combined incidence of these alleles was 72 out of 237 possible pairs (30.4%). Conversely, the first ten alleles distinguishable by binding affinity for the highest number of Gag epitopes were *006:01 (eleven epitopes); *03:01:01 (ten epitopes); *24:32, *24:33, *57:02, *005:08 and *80:01 (nine epitopes); and *09:01, *02:01 and *24:03 (eight epitopes) (Supplementary Table S4). In total, the incidence of these alleles was 21 out of 237 (8.9%). These results indicate that it needs to be determined if there is a relation with the number of binding sites versus susceptibility for BLV or progression to clinical disease.

**Figure 3:**
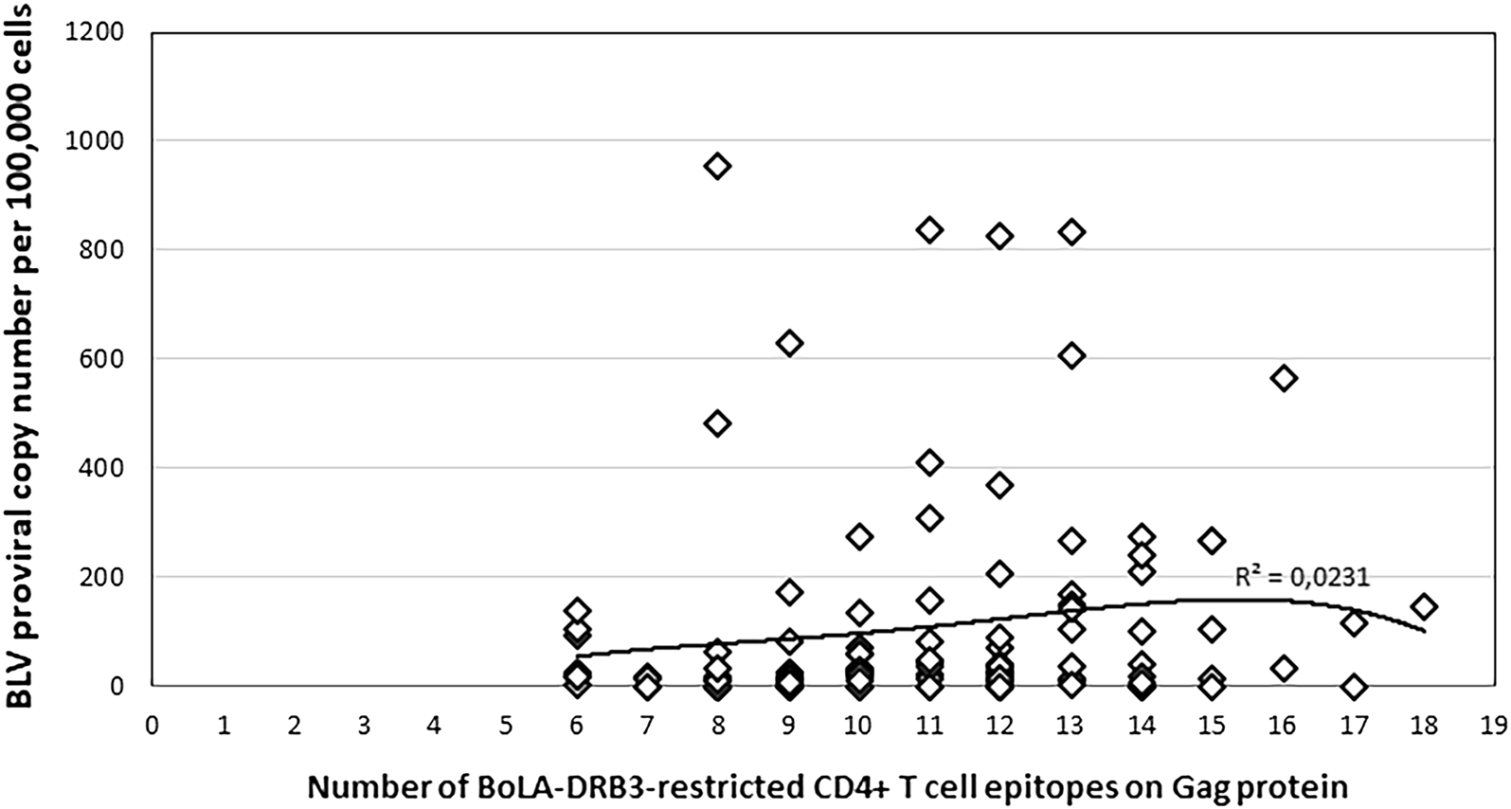
Association between the number of BoLA-DRB3-restricted CD4 + T-cells epitopes on the Gag and BLV proviral load. A polynomial trend line of the fourth degree is plotted in the graph.

Next, the numbers of epitopes were analyzed with respect to proviral copy numbers in BLV-infected cattle. Figure 3 depicts results of such analysis and demonstrates that the number of CD4+ T-cell epitopes on Gag protein identified for different BoLA-DRB3 alleles is not significantly correlated with BLV proviral load (R^2^ = 0.0231, P value = 0.0793, n = 113).

### Association between BoLA-DRB3 alleles, BoLA-DRB3-restricted CD4+ T-cell epitopes and BLV proviral load

We tested 73 alleles for their affinity to particular epitopes and BLV proviral load. Supplementary Fig. S4 shows the distribution of all detected epitopes within Gag protein and their binding affinity for 73 BoLA-DRB3 alleles identified in the current study. Based on literature data, BoLA-DRB3*015:01 and *012:01 alleles are known susceptibility-associated markers related to high PVL in blood cells (> 10,000 per 10^5^), and cattle with susceptible alleles may be at a high risk of BLV transmission via direct contact with healthy cows. In contrast, BoLA-DRB3*09:02, *02:01 and *014:01:01 alleles comprise resistant markers associated with the development of low PVL in blood cells (< 10,000 per 10^5^), and cattle with resistant alleles may be low-risk spreaders for BLV transmission^29–33^. To determine possible associations of BoLA-DRB3 alleles related to susceptibility or resistance to BLV proviral load with BoLA-DRB3-restricted CD4 + T-Cell epitopes, the five (5/73) BoLA-DRB3 alleles like *012:01, *012:03, *015:01, *015:05, *016:01 were placed in a single group (Group A) and marked in red. Group A contained those BoLA-DRB3 alleles associated with a high BLV copy number, as previously described. The five BoLA-DRB3 alleles like *09:02, *09:01, *09:04, *02:01 and *14:01:01 were compiled as a second group (Group B) marked in gray and consisted of the alleles previously correlated in BLV-infected cattle with low proviral load.

**Figure 4:**
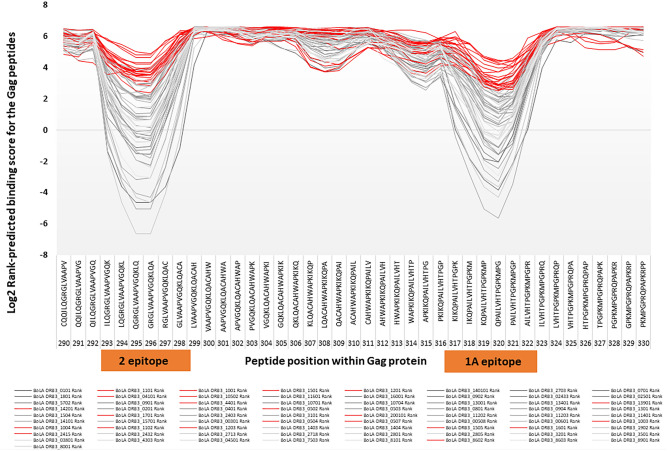
Association between 73 BoLA-DRB3 alleles and two BoLA-DRB3- restricted CD4 + T cell epitopes (1A and 2). Log2 Rank predicted binding score for Gag peptides observed for the BoLA-DRB3 alleles distinguished in the two groups: Group A (n = 22 alleles) marked in red line on the graph; Group B (n = 51 alleles) marked in grey line.

**Figure 5:**
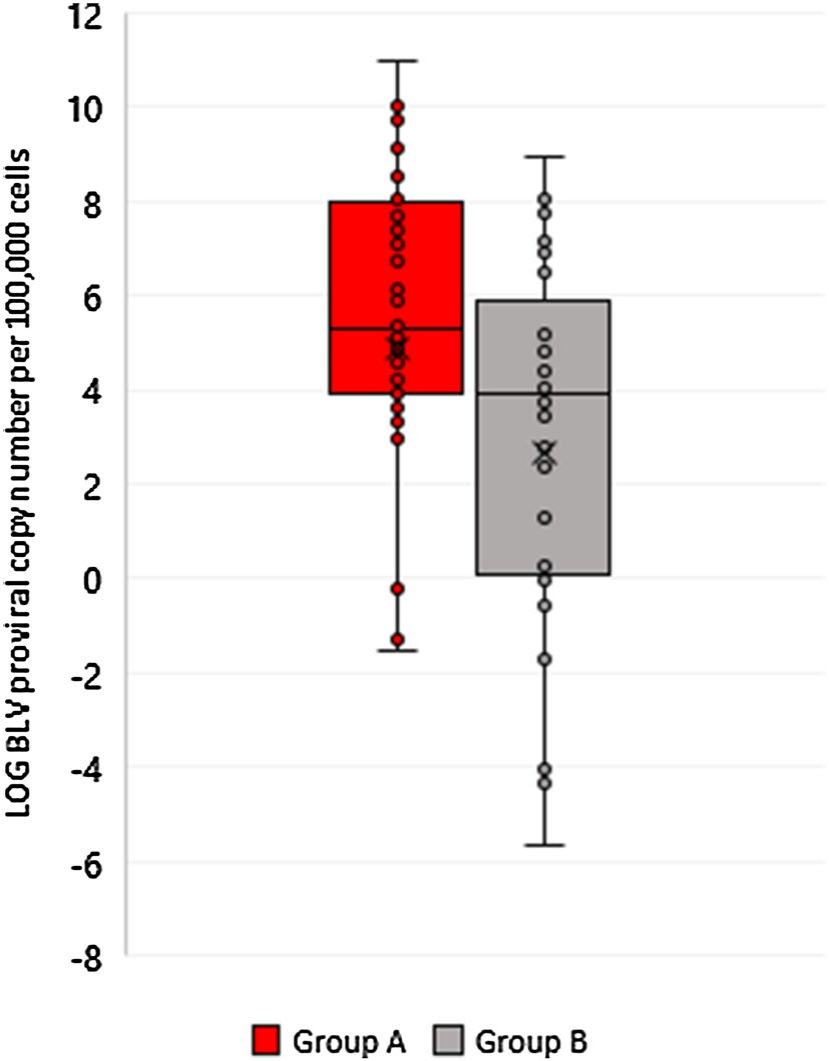
Comparison of BLV copy number between cattle carrying BoLA-DRB3 alleles with no affinity to the Gag protein CD4+ T-cells 1A and 2 epitopes on and alleles with strong affinity to the epitopes using the student t-test for 2 independent means.

As a result of the analysis, none of Group A alleles had affinity for epitopes 1A and 2 (Fig. 4), in contrast to group B alleles’ significant affinity for epitopes 1A and 2. For other epitopes, allele binding from the two groups did not differ and no other patterns were observed between the two groups as far as binding affinity of alleles to these epitopes (Supplementary Fig. S4). Subsequently, the affinity or lack of affinity for epitopes 1A and 2 was determined for the remaining 63 alleles (63/73). As a result, 17 alleles (17/63) were assigned to Group A, (*005:02, *005:04, *005:07, *010:01, *010:03, *010:04, *011:01, *011:02, *017:01, *020:01:01, *024:15, *041:01, *044:01, *086:03, *105:02, *139:01 and *142:01), and remaining 46 alleles (46/63) were classified to group B (*001:01, *003:01, *004:01, *005:03, *005:08,*006:01, *007:01, *008:01, *13:01, *14:03, *14:04, *15:04, *18:01, *19:02, *24:03, *24:32, *24:33, *25:01, *27:03, *27:13, *27:18, *28:01, *28:05, *31:01, *32:01, *35:01, *38:01, *43:03, *45:01, *57:02, *75:03, *80:01, *81:01, *86:02, *89:01, *107:01, *107:04, *112:02, *112:03, *114:01, *116:01, *130:01, *134:01, *141:01, *157:01 and *160:01. Thus, one might infer that Group A alleles’ lack of affinity for epitopes 1A and 2 is related to the number of BLV proviral copies in BLV-infected cattle. Therefore, we conducted a BLV copy number comparison of cattle carrying at least one Group A allele and those with only Group B alleles. Statistical analysis using the student t-test showed that cattle carrying one or both Group A alleles (with no affinity for 1A and 2 epitopes) had a significantly increased number of BLV proviral copies per 1000 cells, as opposed to Group B alleles (*t*-value = 2.06255, P value = 0.040269) (Fig. 5 and Supplementary Table S5).

### Changes in amino acid sequence of Gag vary BoLA-DRB3-peptide binding affinity

**Figure 6:**
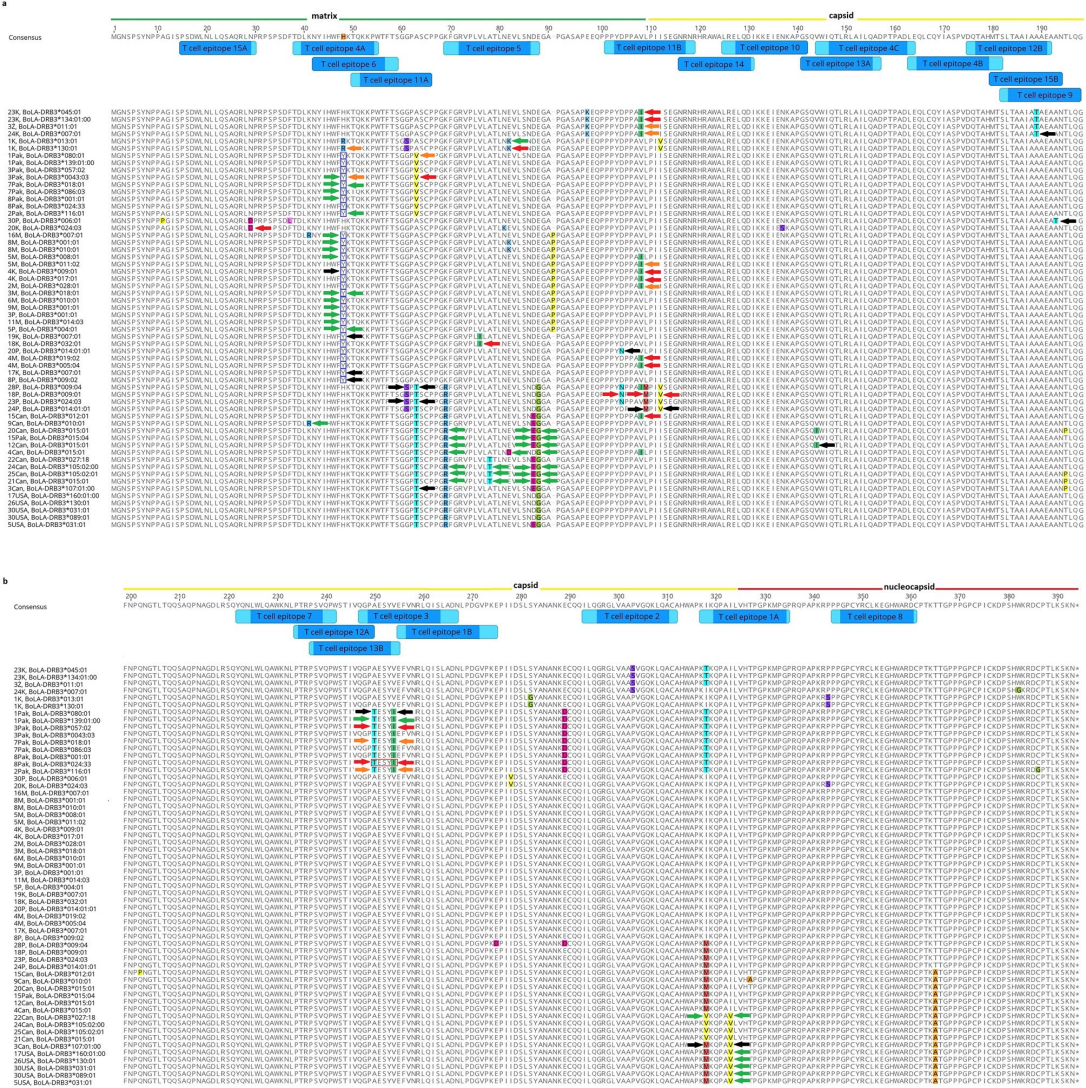
Gag protein sequence alignment for selected BLV isolates, containing amino acid changes within CD4+ T cell epitopes that change the degree of binding affinity of BoLA-DRB3. The names of the isolates and their corresponding BoLA-DRB3 are listed on the left side of the alignment. Amino acid changes, which generate new BoLA-DRB3 binding affinity site are marked with red arrows; amino acid changes that impair BoLA-DRB3 binding affinity sites are marked with green arrows; changes that enhance BoLA-DRB3 affinity are marked with orange arrows, the changes that generate lack of peptides interactions with BoLA-DRB3 are marked with grey arrows. BoLA-DRB3-restricted CD4 + T-cell epitopes along the Gag polyprotein are labeled in the upper part of the figure as blue bars.

The 13 codons, which were identified as the major sites with a process of positive selection were evaluated for epitope affiliation. Out of these 13, 11 codons (84.6%) were located in the following epitopes: codon 48 in epitopes 4A and 6; codons 61 and 63 in epitope 11A; codons 69, 87 and 88 in epitope 5; codons 108, 109 and 112 in epitope 11B; and codons 318 and 323 in epitope 1A. Therefore, to assess the observed mutations’ impact on the binding affinity of BoLA-DRB3, the individual sequences of 125 isolates with corresponding BoLA-DRB3 genotypes were submitted for analysis using NetBoLAIIpan. As result, 58 out of the 125 BLV strains had different amino acid changes (n = 25) in the epitope sequences that was predicted to alter the binding affinity of BoLA-DRB3 to the epitopes. Out of 25, 13 changes were located in positive selection sites (H48R, H48Y, G61S, A63T, A63V, K69R, D87E, E88G, V108I, L109M, I112V, I318M, I323V), and an additional 9 other changes (V76I , A78T, E82D, E82K, D104N, A189T, A193T, A250T, V254I) have predicted a significant effect on the binding affinity of BoLA-DRB3 in combination with different BoLA-DRB3 alleles (Supplementary Table S6). Detailed descriptions of the changes on epitope peptides, and the BoLA-DRB3 epitope binding level are shown in Table 3 and Fig. 6.

**Table 3:**
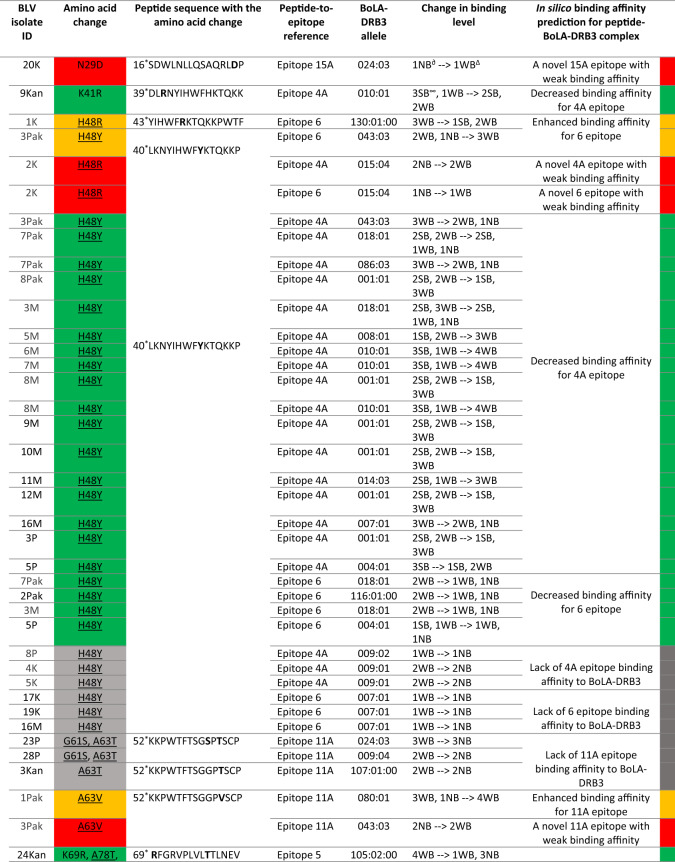

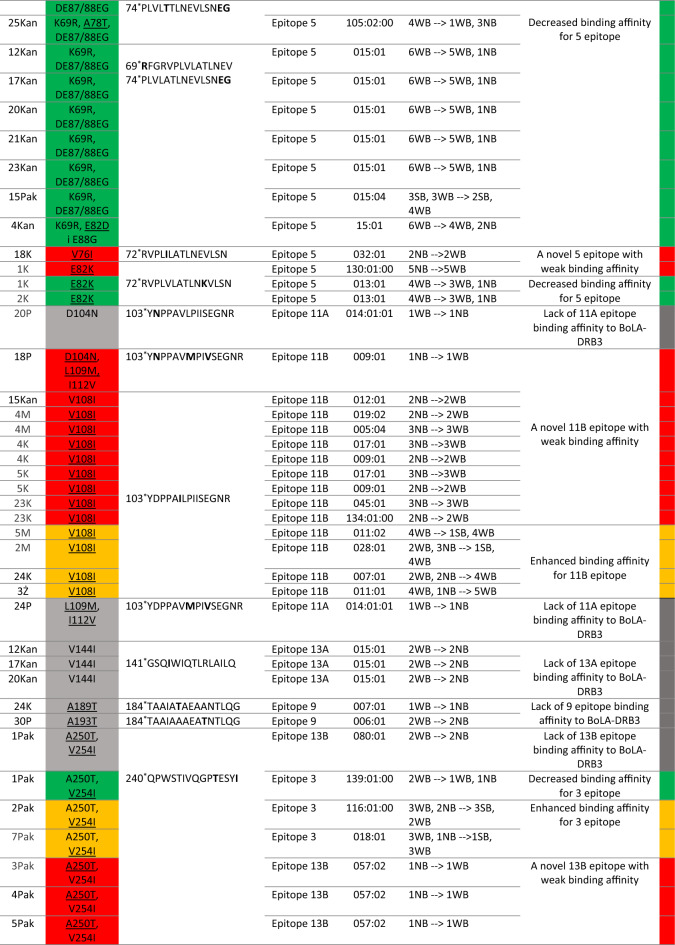

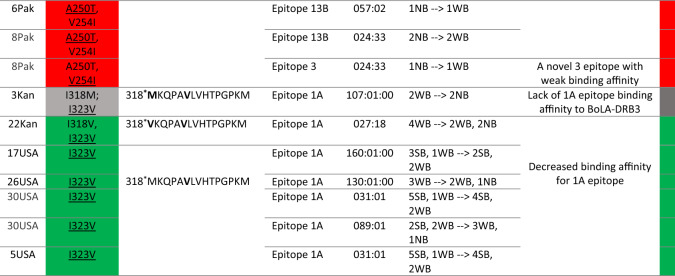
Changes in the amino acid sequence of the Gag peptides that alter the binding affinity of the BoLA-DRB3 alleles.

To specify the level of binding affinity prediction for peptide- BoLA-DRB3 complexes the following colors were used: weaken binding affinity is marked in green; higher binding affinity is marked in orange; new binding affinity is marked in red; amino acid changes that remove epitope binding affinity are marked in grey.

*—peptide position out of 379 isolated peptides along Gag; ^∂^NB—Non-binders (%Rank) > 5%; ^∞^SB—strong binding peptides (%Rank) < 1.0%; ^∆^WB—Weak binding peptides (%Rank) < 5%.

### Analysis of sequence conservation in the binding core of the CD4 + T-cell epitopes

The binding core is the anchoring region of the epitope and is defined as the central nine amino acid (aa) sequence of the predicted 15-mer epitope that is flanked by three aa residues on the N- and C-terminal ends. Therefore, the conservation of the 22 identified putative CD4+ T-cell epitopes was evaluated based on the number of changed amino acid residues in their binding-core sequences (Table 4). The most conserved epitope cores were present in 1B (CA), 4B (CA), 4C (CA), 7 (CA), 8 (NC), 12A (CA), 13A (CA), 14 (CA) and 15A (MA) epitopes, with complete sequence conservation in 125 sequences (100%). Core sequences of epitopes 15B (CA), 2 (CA), 10 (CA) and 12B (CA) showed the second highest level of conservation, in the range of 96.8% (in 121 of 125 sequences) to 99.2% (in 124 of 125 sequences), respectively. Importantly, the mutations found in these core sequences were predicted to no affect the epitope binding affinity to the BoLA-DRB3 molecules (Table 3). Epitopes 1A (CA/NC), 5 (MA), 9 (CA), 3 (CA) and 13B (CA) showed the conservation in the range of 87.2% (in 109 of 125 sequences) to 92.8% (in 116 of 125 sequences), respectively, and contained the mutations I323V, V254I, A78T, E82D, V76I, A193T, and A25T, which may affect epitope binding affinity to certain BoLA-DRB3 molecules. The variable epitopes were 11A (in 59 of 125 MA sequences, 47.2%), 4A and 6 (in 94 of 125 MA sequences, 75.2%) and 11B (in 98 of 125 MA/CA) sequences, 78.4%), containing amino acid changes H48R/Y, G61S, A63V/T, L109M, I112V and V108I, which were predicted to affect binding to BoLA-DRB3.

**Table 4:** The degree of evolutionary conservation of an amino acids in a core sequences of predicted 22 potential CD4 + T-cell epitopes on Gag protein interacting with different BoLA class II alleles.

Epitope	Core sequence	Core sequence conservancy score (%)	Amino acid change in core sequence	Amino acid change in N- and/or C-terminal ends of the epitope
1A	ILVHTPGPK	109/125 (87.2)	I323V	I318M/V
1B	LQISLADNL	125/125 (100.0)	–	–
2	LVAAPVGQK	122/125 (97.6)	–	–
3	VEFVNRLQI	116/125 (92.8)	V254I	–
4A	IHWFHKTQK	94/125 (75.2)	H48R/Y	K41R
4B	YIASPVDQT	125/125 (100.0)	–	–
4C	LAILQADPT	125/125 (100.0)	–	–
5	LVLATLNEV	112/125 (89.6)	V76I, A78T, E82D/K	K69R, DE87/88EG
6	FHKTQKKPW	94/125 (75.2)	H48R/Y	–
7	WKNLPTRPS	125/125 (100.0)	–	–
8	YRCLKEGHW	125/125 (100.0)	–	–
9	IAAAEAANT	114/125 (91.2)	A193T	A189T
10	IKKEIENKA	123/125 (98.4)	–	–
11A	WTFTSGGPA	59/125 (47.2)	G61S, A63V/T	–
11B	PAVLPIISE	98/125 (78.4)	V108I, L109M, I112V	D104N
12A	VQPWSTIVQ	125/125 (100.0)	–	–
12B	MTSLTAAIA	124/125 (99.2)	–	–
13A	WIQTLRLAI	125/125 (100.0)	–	V144I
13B	WSTIVQGPA	116/125 (92.8)	A250T	V254I
14	HRAWALREL	125/125 (100.0)	–	–
15A	LNLLQSAQR	125/125 (100.0)	–	N29D
15B	LTAAIAAAE	121/125 (96.8)	–	–

The table shows the amino acids in the core sequences and peptide flanking regions, which affect peptide–MHC binding and, thereby ultimately also influence the peptide immunogenicity.

## Discussion

The utilisation of bioinformatics to identify T-cell responses to retroviral infections has increased over the last few years^34–38^. The in silico prediction methods NetMHCIIpan and NetBoLAIIpan developed to predict HLA and BoLA class II restricted peptide binding, respectively, have proven to be among the best methods currently available^35,39,40^. Here, we used NetBoLAIIpan prediction method to determine BoLA-DRB3- restricted BLV peptides on p15, p24 and p12 of the Gag polyprotein, with broad BoLA allelic coverage. Of all tested epitope candidates, 11 top-scoring epitopes (1A, 1B, 2, 3, 4A, 4B, 4C, 5 -8) were selected to identify broadly reactive BLV-specific CD4+ T-cell responses, by up to 60% of the analysed BoLA-DRB3 alleles. Another 11 subdominant epitopes (9, 10, 11A, 11B, 12A, 12B, 13A, 13B, 14, 15A, 15B) were predicted to be more restricted to one or more of the donor’s BoLA class II alleles, up to 17.8% of all examined BoLA-DRB3 alleles.

These identified epitopes often overlapped, thus creating long regions within the Gag protein that had BoLA-DRB3 binding affinity. In MA, dominant epitopes occupied 40%, in CA 57% and in NC 47% of the protein sequences. Importantly, the Gag peptide-binding motifs were detected for all bovine BoLA-DRB3 proteins (n = 73) recognized for the 125 cows used in the current studies. Each of the alleles had at least three of the identified target epitopes. The presented data demonstrates a high promiscuity of Gag protein to BoLA-DRB3. BoLA-DRB3-restricted CD4+ T-Cell epitopes 1A and 1B were considered the most promiscuous binders as they contained binding cores of epitopes predicted for 44 different BoLA-DRB3 types. They were located within a conserved part of the p24 region, C-terminal domain, which is required for capsid dimerization, Gag oligomerization and viral formation^41–43^. Moreover, the binding core sequences of the other 12 epitopes (60% of all identified epitopes) had a high conservancy—from 99.2 to 100%. These results show that the majority of the detected epitopes provide a solid guide for vaccine development.

The two previously defined regions of p24 protein, being recognized by specific T-lymphocytes, represented by amino acids 31-PGSQVWIQTLRLAILQADPTPADLE-55 and 141-AESYVEFVNRLQISLADNLPDGVPK-165^13^ were compared with the newly identified epitopes in this study. The first epitope (residues 31–55) region corresponded to T-cell epitopes 4C (144-VWIQTLRLAILQADPTPADLE-164), 13A (142-GSQVWIQTLRLAILQAD-158) and 10 (126-LRELQDIKKEIENKAPGS-143) (specific residues are underlined). The second epitope (residues 141–165) region corresponded to T-cell epitopes 1B (255-EFVNRLQISLADNLPDGVPKE-275), 3 (247-QGPAESYVEFVNRLQISLADN-267), and part of 13B (238-PSVQPWSTIVQGPAESYVE-256). We noted that the previously defined epitopes were nested within newly identified epitopes. Therefore, we strongly suggest that these regions may be alternatively extended by the amino acids contained in the epitopes defined by BoLA-DRB3 II peptide-binding prediction method. Interestingly, in contrast to the second epitope (residues 141–165) region, epitopes 1B, 3 and 13B cover the full length of the major homology region (MHR) 244-IVQGPAESYVEFVNRLQISL-263, which was found to be essential for the stability and folding of the monomer, and hence for viral assembly, maturation and infectivity. This region is conserved throughout the whole retrovirus group and thus offers a novel and stable target for viral vaccines.

In the current study, we detected a greater number of BoLA-DRB3-restricted epitopes than Mager and coworkers^13^. This is likely related to the fact that the previously defined epitope regions are based on PBMCs from only four animals, which represented a particular allele of the BoLA-DRB3 genes interacting with the two specific p24 regions. The type of BoLA-DRB3 alleles in the tested PBMCs in prior published experiments are not defined. Nevertheless, based on our data, it appears that there indeed exist BoLA-DRB3 alleles which have affinity only for one or the other identified epitope regions; there are inter alia: BoLA-DRB3 *007:01, *009:04, *020:01:01 for residues 31–55 and BoLA-DRB3 *002:01, *004:01, *008:01, *010:03, *015:01, *018:01, *025:01:01, *043:03, *081:01, *116:01 and *160:01 for residues 141–165.

We observed that a higher number of Gag protein epitopes recognized by certain BoLA-DRB3 alleles accompanied the alleles associated with BLV resistance in cattle. Interestingly, relatively few of them were observed in the population of BLV-infected animals. Likewise, fewer epitopes recognized by particular BoLA-DRB3 alleles were associated with the BLV susceptible alleles. Noteworthy, the percentage of these alleles in the examined population of virus-infected cows was relatively high. Indeed, the affinity for the interaction of certain BoLA–DRB3 alleles with a longer region of the Gag protein (where epitopes overlap) or more Gag regions may elicit a stronger cellular response. Thus, our results confirm the hypothesis that disease-susceptible cattle may have fewer epitopes than resistant cattle, resulting in weaker immune responses. Moreover, these results indicate a significant role of bovine MHC II polymorphisms in the mapping of BLV epitopes recognized by CD4+ T-cells on viral proteins.

Bai and coworkers studied gp51, gp30 and Tax protein epitopes related to the BoLA–DRB3 genotype and found that fewer CD4+ T-cell epitopes were observed in susceptible cattle than in resistant cattle^19^. Takeshima and colleagues suggested that the BoLA-DRB3 gene may regulate both antigen epitope recognition and the magnitude of the antigen-specific T-cell response that is processed after exposure to infection^23^. Accordingly, our studies confirm that BLV antigens are restricted according to BoLA-DRB3, and that genotyping of cattle is important for determining antigenic epitopes recognized by the bovine immune system.

In this work, we also analyzed whether the number of BoLA-DRB3-restricted epitopes in Gag protein is related to the number of BLV proviral copies in PBMCs in the analyzed DNA samples; however, we did not find a significant correlation. Additionally, Bai and colleagues observed that the number of CD4+ T-cell epitopes was positively related to proviral load, which depended on the BoLA class II genotype^19^. This discrepancy may be due to the fact that the current study used a 25-fold larger group of cattle for the analysis. It is well known that the BLV proviral load varies greatly as it is the result of many different factors such us the time of exposure to the virus, biochemical and hematological factors of the cow, or the age of the cow, to name a few, which can generate erroneous results when experiments are conducted on a small number of animals^44–46^. In comparison, another retrovirus, HIV, specifically targets the HIV Gag peptides by CD4+ T-cells has been associated with lower viremia in both adults and children^47–49^. Ranasinghe and coworkers demonstrated an inverse correlation between viral load and the number of Gag peptides targeted by CD4+ T-cells^50^. Buggert and coworkers confirmed this finding, suggesting that broadly reactive Gag-specific CD4+ T-cell responses could have an impact on HIV disease progression^51^. However, whether the frequent targeting of Gag peptides is the cause or the consequence of the reduced viremia remains to be clarified.

Nevertheless, an association polymorphisms of the BoLA-DRB3 gene with BLV PVL is described in the literature^23,31,45,52,53^. Published data indicates some BoLA-DRB3 alleles such as *15:01, *12:01 and *16:01 are associated with high PVL in BLV-infected cattle but BoLA-DRB3 alleles like *09:02, *02:01 and *14:01:01 are associated with low PVL^29–32^. Of the 22 epitopes, we found two epitopes—1A (317-KIKQPAILVHTPGPKMPGPR-336) and 2 (293-ILQGRGLVAAPVGQKLQACA-312) that were significantly related to cattle resistant to developing high BLV proviral load. These epitopes were located in CTD-CA and between CA/NC proteins, respectively, highly conserved regions for retroviruses (Supplementary Fig. S6). In addition, these epitopes were broadly recognized for most of the BoLA-DRB3 alleles (70%). Interestingly, epitopes 1A and 2 were not recognizable by the types of BoLA-DRB3 alleles, which were previously reported in the literature as being associated with the development of subclinical infection and high BLV PVL^29–31,54,55^. Some of these have never been investigated for PVL dependence therefore, additional functional studies are required to further confirm these findings. Nevertheless, in the case of HIV, there are certain epitopes that determine resistance to infection^56^. Our results suggest that the 1A and 2 epitopes may have a key and powerful effect in inducing a strong cellular response and fighting BLV within the host. It is noteworthy that epitopes 1A and 2 were the strongest epitopes to which the most alleles bound. Therefore, they seem to be an indispensable element that would be instructive in the design of synthetic peptide vaccine.

Antigenic variation within T-cell epitopes has been demonstrated for HIV-1, and this 'antigenic escape' may be responsible for viral persistence. Generally, although external proteins are highly immunogenic, antigenic shift limits their capacity to provide cross-protective immunity to novel viral strains. In contrast, the internal proteins are more conserved and may better mediate cross-protective T-cell responses^57,58^.

BLV exhibits less genetic variation among strains as compared with most other retroviruses, and the genomes of viruses isolated from multiple countries around the world share approximately between 94.5 and 99.5% of their nucleotide sequences. However, variation within the sequences encoding the Gag protein is poorly characterized. In our study the pairwise identity for 125 gag nucleotide sequences was 97.3%. Despite the internal proteins MA, CA and NC that exhibit higher levels of conservation relative to SU (gp51), sequence variation was still present, in which most sequence variation can be attributed to a single mutation. Based on the resulting proviral mutation profile, we revealed that the mutations are driven by immune selection pressure, suggesting mechanisms of positive selection and mutation hotspots.

Diversified positions were preferentially located within bovine CD4+ T-cell epitopes. Of 13 hotspots, 7 were located in the 9-mer core epitopes and had predicted a significant effect on the binding affinity of BoLA-DRB3 molecules. This is consistent with what is known about the peptide-binding core of epitopes that primarily interact with the BoLA-DR antigen binding groove. The peptide–BoLA-DR binding affinity is primarily determined by the amino acid sequence of the peptide binding core^59^. However, it has been shown that peptide flanking regions (PFRs) on either side of the binding core affect peptide–BoLA-DR binding and thereby ultimately also influence the peptide immunogenicity^40^. Indeed, 4 hotspots were located in PFRs and have input on binding affinity. These mutations may upend the presentation of virus-derived peptides via BoLA-DR. Based on the obtained results, certain mutations reduced while other mutations increased the affinity to bovine MHCII. Additionally, some mutations exhibited a neutral affinity. On the basis of our analysis of mutations, we selected 12 mutant peptides with predicted decreased BoLA-DR-binding strength 19 and mutant peptides with predicted increased BoLA-DR-binding strength for further biophysical and functional analyses. Our study provides evidence that single nonsynonymous mutations in BLV can subvert the immune response to CD4+ T-cell epitopes.

Our hypothesis was that the substitution of single amino acids in CD4+ T-cell epitope may influence the BoLA-DRB3 binding affinity and that nsSNP might be associated with variations in individual immune responses to antigens and susceptibility or resistance to disease. There are therefore many factors that make it difficult to predict peptide binding affinities to BoLA-DRB3 molecules, including the polymorphic sites of Gag epitopes. However, without functional analysis, the impact of single anchor residue substitutions on the response of CD4+ T-cells is still unclear. This study does not allow direct conclusions to be drawn concerning potential selection pressures, which shape the mutational landscape of CD4+ T-cell epitopes. This would invariably involve accounting for the BoLA-DRB3 genotype of all individuals from whom BLV genomes were sequenced. Moreover, how T-cell escape mutations within BLV are maintained during virus transmission between individuals with differing BoLA types and how viruses carrying epitope mutations affect disease severity requires further investigation.

Many CD4+ T-cell epitopes for BLV have been described in this study. The CD4+ T-cell response against BLV was associated with broad epitope recognition of, on average, 6 CD4+ T-cell epitopes per antigen per BoLA-DRB3 allele, which raises the question whether and how mutations in single epitopes affect virus control^60^. This may be of particular importance for BLV subunit vaccines to induce responses against an unlimited number of CD4 epitopes. These results highlight the capacity of BLV to evade cellular immune responses through sporadically emerging mutations in BoLA-BRB3 epitopes.

Taking into account the very conservative and wide range of identified epitopes and, on the other hand, the lack of progress in obtaining an effective vaccine, the new discovery has a high chance of success. The new vaccine could be an important element in protecting herds against BLV infections, especially in dairy cattle, where this category of cattle is especially susceptible to BLV infection^61^. Moreover, preventive vaccinations based on selected peptide immunogens could become an integral part of BLV eradication programs^62,63^. Finally, the importance of immunopeptidomics should be emphasized in subsequent studies taking into account other exotic and local cattle breeds as well as the circulation of endemic BLV variants.

## Conclusions

In the present study, BLV Gag protein was characterized by immunoinformatic techniques to identify potential T-cell epitopes. Twenty-two BoLA-DRB3 class II epitopes were available across the entire BLV Gag polyprotein, however the p24 protein was identified as the main target for recognition by antigen-specific CD4+ T-lymphocytes. The thirteen broadly conserved BoLA-DRB3-restricted CD4+ T-cell epitopes shared between BLV isolates from different countries and 9 epitopes with changes in the binding core were identified. Among them two promiscuous conserved pBoLA-(gag)peptides, 1A and 2, related to hosts that mounted a successful host–pathogen immune response (animals with low proviral load) were discovered. We believe the newly-identified pBoLA-(gag) peptides, together with additional peptides that have been shown within gp51, gp30 and Tax proteins, will be important for inclusion in a multivalent antigen peptide vaccine for BLV that can provide protection against BLV infection caused by geographically distant viral strains in cattle that express different BoLA class II DRB3 haplotypes.

## Methods

### Ethics declaration

The study was approved by the Veterinary Sciences Animal Care Committee No. AC21-0210, Canada; the Institutional Animal Care and Use Committee No. PROTO202000096 from 4/13/2020 to 4/14/2023, Michigan State University, United States; the Ethics Review Board, COMSATS Institute of Information Technology, Islamabad, Pakistan, no. CIIT/Bio/ERB/17/26 and the Bioethics Commission No. 06-18 on 30 January 2018, Almaty, Kazakhstan. Blood samples from Polish and Moldovan cattle, naturally infected with BLV, were selected from collections at local diagnostic laboratories as part of the Enzootic bovine leukosis (EBL) monitoring program between 2012 and 2018 and sent to the National Veterinary Research Institute (NVRI) in Pulawy for confirmation study. The approval for collection of these samples from ethics committee was not required according to Polish regulation (“Act on the Protection of Animals Used for Scientific or Educational Purposes”, Journal of Laws of 2015).

### Sample collection and preparation

A total of 125 DNA samples obtained from blood of naturally BLV-infected cattle from Canada, United States, Poland, Moldova, Pakistan and Kazakhstan were used for this study. Seventy-six of them were archival DNA samples obtained between 2013 and 2018 as described in our previous studies on samples from Poland (n = 22)^64,65^, Moldova (n = 14)^66^, Pakistan (n = 20)^67^ and Kazakhstan (n = 21)^68^. Between 2020 and 2021 48 peripheral blood and serum samples from naturally BLV-infected cattle were obtained from three dairy farms of Alberta, Canada and two dairy farms of Michigan, US (see Table 1). All cattle were positive for anti-BLV antibodies, as determined by commercially available ELISA kit (IDEXX Leukosis Serum X2 Ab Test, IDEXX). Genomic DNA were isolated using a Quick DNA Miniprep Plus kit (Zymo Research) and a DNeasy Blood & Tissue Kit (Qiagen) for Canadian (n = 24) and US (n = 24) whole blood samples, respectively, following the manufacturer’s protocol.

### PCR amplification of BoLA-DRB3 exon 2 and sequencing

A 247 bp fragment of BoLA-DRB3 exon 2 containing the hypervariable domain was amplified on all DNA samples (n = 125) by a PCR as described in a previous study^69^. Reactions were carried out in 30 µl final volume containing 1 × Pol Buffer B, 2.5 mM MgCl_2_, 115 µM of each dNTP, 0.3 μM of each primer, 0.4 U of OptiTaq DNA Polymerase and 12 ng genomic DNA. The thermal cycling protocol was initial denaturation at 94°C for 3 min; 35 cycles at 94°C for 20 s, 60°C for 20 s, and at 72°C for 1 min, followed by final extension at 72°C for 5 min. Reactions were carried out in a TAdvanced Twin PCR Thermal Cycler (Biometra). PCR products were clean-ed up using ExoSAP-IT (Applied Biosystems) by incubation at 37°C for 15 min, followed by ExoSAP-IT inactivation by heating to 80°C for 15 min. The DNA was directly sequenced (Genomed SA Company) with BoLA-DRB3 exon 2 forward and reverse primers HL030 (5′-AGATCTATCCTCTCTCTGCAGCACATTTCC-3′) and HL031 (5′-CGCGCTCACCTCGCCGCT-3′) respectively^69^.

### Sequence-based typing of BoLA-DRB3 alleles

The raw sequences of the 247 bp fragment of BoLA-DRB3 gene were visualized and aligned in Geneious Prime software. Consensus sequences were generated, in which the heterozygous positions were assigned ambiguity codes according to the IUPAC coding system (Supplementary file 1). The consensus sequences were initially compared to the 389 DRB3 allele sequences deposited in the IPD-MHC database (available via web https://www.ebi.ac.uk/ipd/) using Haplofinder script (http://bioinformatics.roslin.ed.ac.uk/haplofinder/haplofinder.py) and Python 2.7.18 software (https://www.python.org)^70^. To confirm that the assigned alleles did indeed match the pairwise sequence combination, a custom BLAST database was created in Geneious Prime and the data from IPD-MHC were implemented and the query centric alignment was generated using Megablast configuration for highly similar sequences and Low Complexity Filter with Scoring: 1–2 and max E-value of < 0.05.

### Amplification and sequencing of *gag* gene

The full-length 1353 bp BLV *gag* gene was amplified from DNA samples (n = 125) by nested PCR using oligonucleotide primers as previously reported^71^ (Supplementary Fig. S5). Both rounds of amplification was performed using PrimeSTAR GXL DNA Polymerase (Takara Bio). Thermal cycling parameters were as follows: initial denaturation at 98 °C for 2 min followed by 38 cycles (36 cycles for the second round) of denaturation at 98 °C for 15 s, annealing at 60 °C for 15 s, extension at 68 °C for 1 min 50 s (1 min 30 s for the second round) and final extension at 72 °C for 5 min. PCR products were separated by electrophoresis on 1.5% agarose gel containing SimplySafe (EURx) and purified using a NucleoSpin Extract II Kit (Marcherey Nagel GmbH & Co KG). Sequencing was performed by Genomed SA Company (Warsaw, Poland). Each sequencing reaction was carried out using: 3 µl BigDye™ Terminator v3.1 Ready Reaction Mix, 1 µl BigDye™ Terminator v1.1 & v3.1 5× Sequencing Buffer, 5 pmol of the primer and 150 ng of DNA were mixed in a final 10 µl volume.

Cycle sequencing was performed in 100 µl PCR tubes as follows: incubation at 96 °C for 1 min as the initial denaturation step followed by 25 cycles of 96 °C for 10 s, 54 °C for 5 s, and 60 °C for 4 min according to standard protocol routinely used by the Genomed SA Company. Prior to purification, the reaction mix was incubated for 10 min at 4 °C. Purified reaction products were separated by electrophoresis on the 3730 xl DNA Analyzer (Thermo Fisher) according to the manufacturer’s instructions. The following pairs of primers were used to direct sequencing as shown in Supplementary Fig. S5.

### Analysis of genetic variation among the *gag* gene sequences

The raw sequence reads in both directions were proofread and analyzed in Geneious Prime 2021.0.1 (Biomatters Ltd). The consensus sequences were determined and deposited in the GenBank database under accession numbers OP146492-OP146601 (Table 1 and Supplementary Fig. S2). The amino acid sequences were translated according to the IUPAC amino acid code and aligned using the Clustal Omega 1.2.2 algorithm. The substitution analysis and pairwise genetic distance assessment were performed in Geneious Prime. The Shannon’s entropy (a quantitative measure of diversity in the alignment, where H = 0 indicates complete conservation) was estimated using BioEdit v. 7.2.5 software^72^ for each position in the amino-acid alignment, and the entropy values were summed for each Gag domain. The DnaSP v. 6.12.03 program was used to quantify strength of selection by comparing synonymous substitution rates (dS) with nonsynonymous substitution rates (dN)^73^. Ratio of dN/dS < 1 was interpreted as negative or purifying selection and > 1 was interpreted as positive selection pressure.

### Prediction of BoLA-DRB3-presented epitopes

A BoLA-DRB- peptide binding affinity prediction method, NetBoLAIIpan—1.0 was used to predict the Gag protein peptides presented by the BoLA-DRB3 molecule. The consensus Gag protein sequence calculated for the 125 sequences alignment was submitted to the server in *FASTA* format. For this analysis, a comprehensive list of BoLA molecules available in the server for prediction was updated with new additional sequences deposited in the IPD-MHC database. The prediction values represented by likelihood for BoLA antigen presentation and %Rank score were calculated. In detail, the percentile rank for each Gag peptide was generated by comparing its score against the scores of 100,000 random natural peptides^74^. For example, if a peptide was assigned a rank of 1%, it meant that its predicted affinity was among the top 1% scores for the specified molecule. The %Rank score of < 1.0, ≥ 1 to < 5.0 and ≥ 5.0 were interpreted as strong binders, weak binders, and non-binders, respectively. All %Rank score predictions ≤ 5.0 were considered as epitope peptides.

### Proviral load quantification

The qPCR assays for the BLV *pol* gene and H3F3A gene were performed according to previously published methods^75,76^. Briefly, genomic DNA was amplified using primers and probes for *pol* gene and *H3F3A* gene, and QuantiTect Multiplex PCR NoROX master mix (Qiagen AG GmbH) according to the protocol: 95 °C for 15 min, followed by 45 cycles each of 94 °C for 60 s and 60 °C for 60 s. Ten-fold dilutions of the pBLV1 and pH3F3A plasmids from 1 × 10^6^ copies/μl to 100 copies/μl were used as the standard to estimate BLV copy numbers. The BLV proviral load (copies/1000 cells) was calculated as [copies of BLV *pol* gene/(copies of H3F3A gene/2)] × 1000 cells^75^.

### Statistical analysis

The correlation between number of CD4 + T-cell epitopes and proviral load in BLV-infected cattle was calculated using the Spearman non-parametric test (with P value < 0.05). The difference in proviral DNA copy number between two groups of samples with different BoLA-DRB3 genotypes was calculated using the Student’s t-test, where a P value of < 0.05 was considered to be significant. The statistical analysis was performed using STATISTICA ver. 10 (StatSoft).

### Modeling of peptides

A PDB file of a putative BLV Gag structure^77^ was used to identify the location of the epitopes. The structure was opened in UCSF Chimera^78^ and the surface representation was used to display epitopes by color.

### Supplementary Information

Supplementary Figure S3.

Supplementary Figure S4.

Supplementary Information 3.

Supplementary Information 4.

Supplementary Table S1.

Supplementary Table S3.

Supplementary Table S4.

Supplementary Table S6.

## Data Availability

The *gag* gene sequences generated and analyzed during the current study are available in the GenBank repository under accession numbers OP146492-OP146601. The raw sequences of the fragment of BoLA-DRB3 gene with assigned ambiguity codes, according to the IUPAC coding system, in the heterozygous positions generated and analyzed in this study are available in Supplementary file 1.
