# Supplementary figures and images for: An immunoinformatics study reveals a new BoLA-DR-restricted CD4+ T cell epitopes on the Gag protein of bovine leukemia virus
Source: Sci Rep. 2023 Dec 15;13:22356. doi: 10.1038/s41598-023-48899-4 (PMC10724172; doi:10.1038/s41598-023-48899-4)

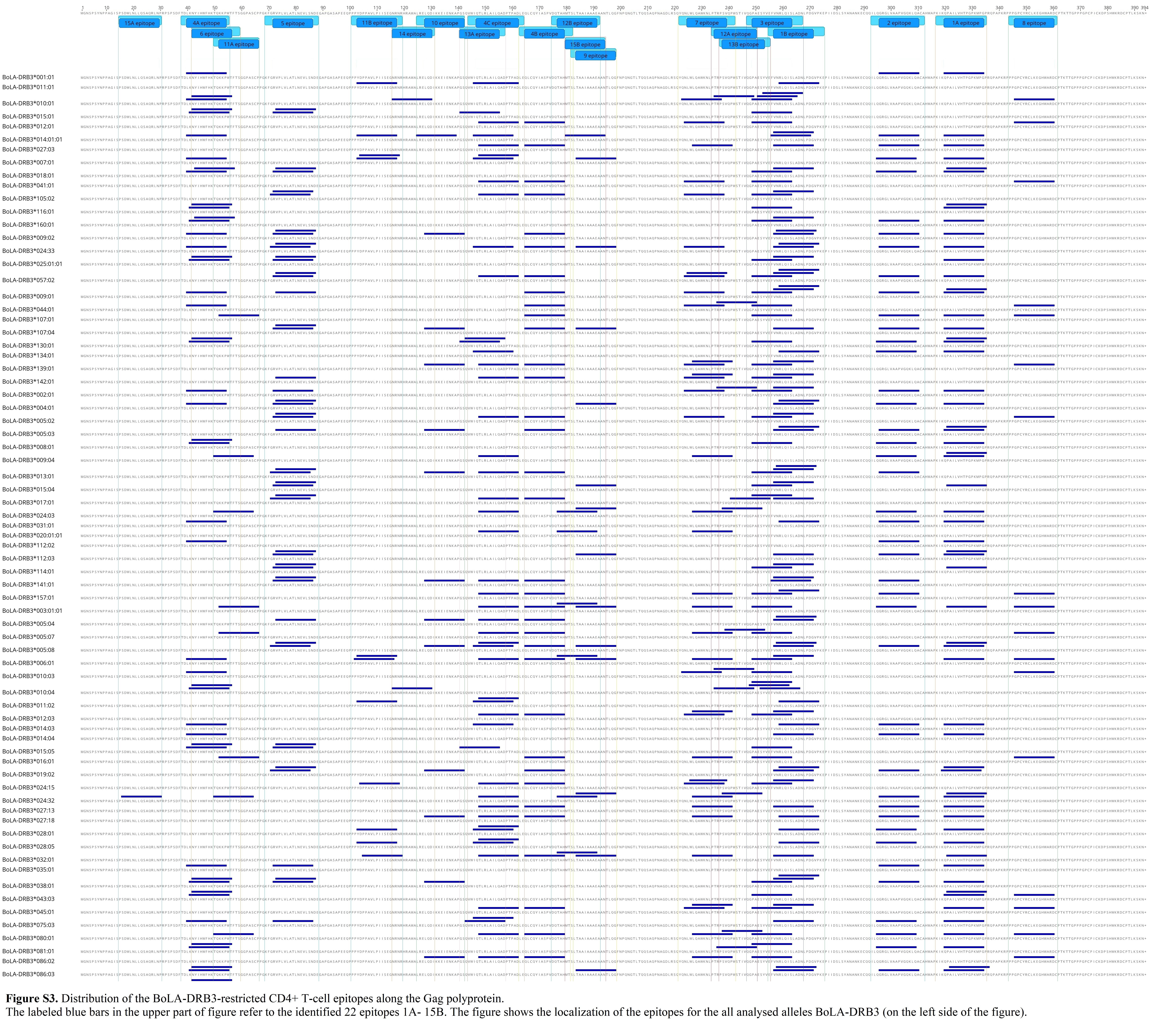

Supplement: Supplementary file 1 — Supplementary Figure S3. [file 41598_2023_48899_MOESM1_ESM.jpg]
