# Supplementary material for: An immunoinformatics study reveals a new BoLA-DR-restricted CD4+ T cell epitopes on the Gag protein of bovine leukemia virus
Source: Sci Rep. 2023 Dec 15;13:22356. doi: 10.1038/s41598-023-48899-4 (PMC10724172; doi:10.1038/s41598-023-48899-4)
Supplement: Supplementary file 4 — Supplementary Information 4. [file 41598_2023_48899_MOESM4_ESM.pdf]

Supplementary information

An immunoinformatics study reveals a new BoLA-DR-restricted CD4+ T cell epitopes on the Gag protein of Bovine Leukemia Virus

Aneta Pluta[1*], Tasia Kendrick[2], Frank van der Meer[3], Sulav Shrestha[3], Dominic Qualley[4], Paul Coussens[2], Marzena Rola-Łuszczak[1], Anna Ryło[1], Ali Sakhawat[5,6,7], Saltanat Mamanova[8], Jacek Kuźmak[1]

[1]Department of Biochemistry, National Veterinary Research Institute, 24-100 Puławy, Poland;

*Address correspondence to aneta.pluta@piwet.pulawy.pl

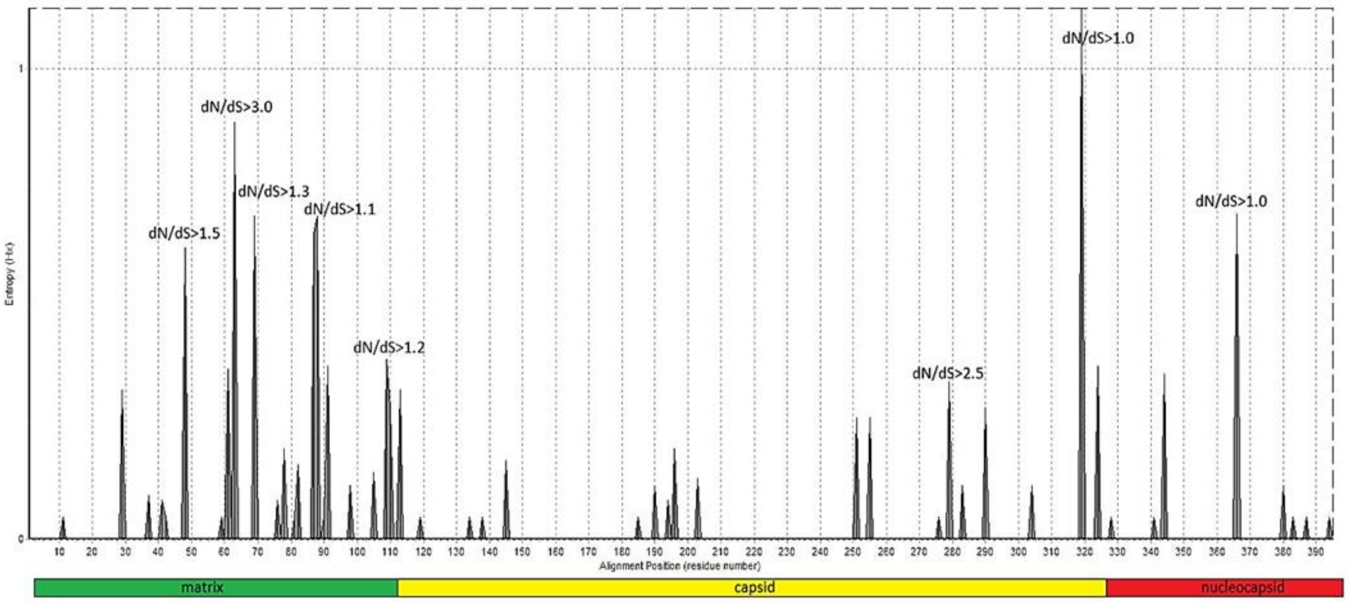

Figure S1. Shannon Entropy (Hx) plot of the Gag sequences (n=125) show diversity along the whole sequence. Alignment positions with positive selection (dN/dS>1) are marked above the entropy bars. The location of the MA (green), CA (yellow) and NC (red) domains is on the bar along the horizontal axis.

```
          ----P1----------------------------------------------------Matrix-------------------------------P2-----------Capsid-NTD------
                   10        20        30        40        50        60        70        80        90       100       110       120       130
          ----:----|----:----|----:----|----:----|----:----|----:----|----:----|----:----|----:----|----:----|----:----|----:----|----:----|--
Consensus MGNSPSYNPPAGISPSDWLNLLQSAQRLNPRPSPSDFTDLKNYIHWFHKTQKKPWTFTSGGPASCPPGKFGRVPLVLATLNEVLSNDEGA-PGASAPEEQPPPYDPPAVLPIISEGNRNRHRAWALRELQDI 131
1P        ............................................................S.T.....R..................G.T-.................IM..V................... 131
28P       ............................................................S.T.....R..................G..-.............N...IM..V................... 131
2P        ............................................................S.T.....R..................G..-..................M...................... 131
21P       ............................................................S.T.....R..................G..-.............N....M..V................... 131
18P       ............................................................S.T.....R..................G..-.............N....M..V................... 131
23P       ............................................................S.T.....R..................G..-.............N....M..V................... 131
16P       ............................................................S.T.....R..................G..-..................M..V................... 131
19P       ............................................................S.T.....R..................G..-..................M..V................... 131
24P       ............................................................S.T.....R..................G..-..................M..V................... 131
6P        ............................................................S.T.....R..................G..-..................M..V................... 131
11Kan     ..............................................................T.....R.................EG..-.................I....................... 131
15Kan     ..............................................................T.....R.................EG..-.................I....................... 131
10Pak     ..............................................................T.....R..................G..-...........................S............. 131
17USA     ..............................................................T.....R..................G..-......................................... 131
26USA     ..............................................................T.....R..................G..-......................................... 131
27USA     ..............................................................T.....R..................G..-......................................... 131
30USA     ..............................................................T.....R..................G..-......................................... 131
24USA     ..............................................................T.....R..................G..-......................................... 131
4Kan      ..............................................................T.....R............D.....G..-.................I....................... 131
16USA     ..............................................................T.....R..................G..-.................I....................... 131
18Kan     ..............................................................T.....R..................G..-......................................... 131
13Kan     ....................................Y.........................T.....R..................G..-......................................... 131
18USA     ..............................................................T.....R..................G..-......................................... 131
20USA     ..............................................................T.....R..................G..-......................................... 131
28USA     ..............................................................T.....R..................G..-......................................... 131
31USA     ..............................................................T.....R..................G..-......................................... 131
29USA     ..............................................................T.....R..................G..-......................................... 131
19Kan     ..............................................................T.....R........T........EG..-......................................... 131
23Kan     ..............................................................T.....R........T........EG..-......................................... 131
25Kan     ..............................................................T.....R........T........EG..-......................................... 131
21Kan     ..............................................................T.....R........T........EG..-......................................... 131
22Kan     ..............................................................T.....R........T........EG..-......................................... 131
24Kan     ..............................................................T.....R........T........EG..-......................................... 131
9Kan      ........................................R.....................T.....R.................EG..-......................................... 131
20Kan     ..............................................................T.....R.................EG..-......................................... 131
12Kan     ..............................................................T.....R.................EG..-......................................... 131
17Kan     ..............................................................T.....R.................EG..-......................................... 131
1Kan      ..............................................................T.....R.................EG..-......................................... 131
3Kan      ..............................................................T.....R.................EG..-......................................... 131
5USA      ..............................................................T.....R.................EG..-......................................... 131
21Pak     ..............................................................T.....R.................EG..-......................................... 131
11Pak     ..............................................................T.....R.................EG..-......................................... 131
19Pak     ..............................................................T.....R.................EG..-......................................... 131
12Pak     ..............................................................T.....R.................EG..-......................................... 131
5Kan      ..............................................................T.....R.................EG..-......................................... 131
6USA      ..............................................................T.....R.................EG..-......................................... 131
7USA      ..............................................................T.....R.................EG..-......................................... 131
4USA      ..............................................................T.....R.................EG..-......................................... 131
10USA     ..............................................................T.....R.................EG..-......................................... 131
15USA     ..............................................................T.....R.................EG..-......................................... 131
3USA      ..............................................................T.....R.................EG..-......................................... 131
8USA      ..............................................................T.....R.................EG..-......................................... 131
12USA     ..............................................................T.....R.................EG..-......................................... 131
13USA     ..............................................................T.....R.................EG..-......................................... 131
1USA      ..............................................................T.....R.................EG..-......................................... 131
2USA      ..............................................................T.....R.................EG..-......................................... 131
14Pak     ..............................................................T.....R.................EG..-......................................... 131
15Pak     ..............................................................T.....R.................EG..-......................................... 131
17Pak     ..............................................................T.....R.................EG..-......................................... 131
18Pak     ..............................................................T.....R.................EG..-......................................... 131
22Pak     ..............................................................T.....R.................EG..-......................................... 131
23Pak     ..............................................................T.....R.................EG..-......................................... 131
10Kan     ..............................................................T.....R.................EG..-......................................... 131
7Kan      ..............................................................T.....R.................EG..-......................................... 131
```

```
          -----P1---------------------------------------------------------------Matrix-----------------------P2--------------Capsid-NTD-------
                  10        20        30        40        50        60        70        80        90       100       110       120       130
          ----:----|----:----|----:----|----:----|----:----|----:----|----:----|----:----|----:----|----:----|----:----|----:----|----:----|--
8Kan      ..............................................................T.....R.................EG..-.........................................  131
33USA     ..............................................................T.....R.................EG..-.........................................  131
14Kan     ..............................................................T.....R.................EG..-.........................................  131
16Kan     ..............................................................T.....R.................EG..-.........................................  131
2Kan      ..............................................................T.....R.................EG..-.........................................  131
30P       ..........P.................D.......L.....................................................-.........................................  131
4Ż        ..........................................................T...............................-.....................V...................  131
7K        ............................D.............................................................-.........................................  131
20K       ............................D...................................................K.........-.........................................  131
1M        ............................D.............................................................-.........................................  131
15K       ............................D.............................................................-.........................................  131
11Ukr     ............................D.............................................................-.........................................  131
14M       ............................D.............................................................-.........................................  131
14K       ............................D.............................................................-.........................................  131
9K        ............................D.............................................................-.........................................  131
10K       ............................D.............................................................-.........................................  131
11K       ............................D.............................................................-.........................................  131
12K       ............................D.............................................................-.........................................  131
2Pak      ...............................................Y..............V...........................-.........................................  131
1Pak      ...............................................Y..............V...........................-.........................................  131
3Pak      ...............................................Y..............V...........................-.........................................  131
4Pak      ...............................................Y..............V...........................-.........................................  131
5Pak      ...............................................Y..............V...........................-.........................................  131
6Pak      ...............................................Y..............V...........................-.........................................  131
7Pak      ...............................................Y..............V...........................-.........................................  131
8Pak      ...............................................Y..............V...........................-.........................................  131
9Pak      ...............................................Y..............V...........................-.........................................  131
1K        ...............................................R............S....................K........-.....................V...................  131
2K        ...............................................R............S....................K........-.....................V...................  131
24K       ..........................................................................................-......K..........I.......................  131
23K       ..........................................................................................-......K..........I.......................  131
3Ż        ..........................................................................................-......K..........I.......................  131
8M        ...............................................Y.................................K........P.........................................  132
2M        ...............................................Y..........................................P.................I.......................  132
5M        ...............................................Y..........................................P.................I.......................  132
4K        ...............................................Y..........................................P.................I.......................  132
5K        ...............................................Y..........................................P.................I.......................  132
16M       ........................................R......Y..........................................P.........................................  132
10M       ...............................................Y..........................................P.........................................  132
11M       ...............................................Y..........................................P.........................................  132
12M       ...............................................Y..........................................P.........................................  132
9M        ...............................................Y..........................................P.........................................  132
6M        ...............................................Y..........................................P.........................................  132
7M        ...............................................Y..........................................P.........................................  132
3P        ...............................................Y..........................................P.........................................  132
3M        ...............................................Y..........................................P.........................................  132
5P        ...............................................Y..........................................P.........................................  132
7P        ..........................................................................................-.................I.......................  131
4P        ..........................................................................................-.........................................  131
17P       ............................................................D.............................-.................I.......................  131
22K       .........................................S................................................-.........................................  131
12P       ..........................................................................................-.........................................  131
4M        ...............................................Y..........................................-.................I.......................  131
18K       ...............................................Y...........................I..............-.........................................  131
19K       ...............................................Y...........................I..............-.........................................  131
14P       ..........................................................................................-.........................................  131
15P       ..........................................................................................-.........................................  131
27P       ..........................................................................................-.........................................  131
8P        ...............................................Y..........................................-.........................................  131
17K       ...............................................Y..........................................-.........................................  131
```

```
          ---------------------------------------------------------------------------Capsid-NTD----------------------P3-------------------------
                 140       150       160       170       180       190       200       210       220       230       240       250       260
          --:----|----:----|----:----|----:----|----:----|----:----|----:----|----:----|----:----|----:----|----:----|----:----|----:----|----
Consensus KKEIENKAPGSQVWIQTLRLAILQADPTPADLEQLCQYIASPVDQTAHMTSLTAAIAAAEAANTLQGFNPQNGTLTQQSAQPNAGDLRSQYQNLWLQAWKNLPTRPSVQPWSTIVQGPAESYVEFVNRLQIS 263
1P        ....................................................A............................................................................... 263
28P       .................................................................................................................................... 263
2P        ............I....................................................................................................................... 263
21P       .................................................................................................................................... 263
18P       .................................................................................................................................... 263
23P       .................................................................................................................................... 263
16P       .................................................................................................................................... 263
19P       .................................................................................................................................... 263
24P       .................................................................................................................................... 263
6P        .................................................................................................................................... 263
11Kan     ......................................................................P............................................................. 263
15Kan     ......................................................................P............................................................. 263
10Pak     .................................................................................................................................... 263
17USA     .................................................................................................................................... 263
26USA     .................................................................................................................................... 263
27USA     .................................................................................................................................... 263
30USA     .................................................................................................................................... 263
24USA     .................................................................................................................................... 263
4Kan      .................................................................................................................................... 263
16USA     .................................................................................................................................... 263
18Kan     .................................................................................................................................... 263
13Kan     .................................................................................................................................... 263
18USA     .................................................................................................................................... 263
20USA     .................................................................................................................................... 263
28USA     .................................................................................................................................... 263
31USA     .................................................................................................................................... 263
29USA     .................................................................................................................................... 263
19Kan     .R.................................................................................................................................. 263
23Kan     ...............................................................P.................................................................... 263
25Kan     ...............................................................P.................................................................... 263
21Kan     ...............................................................P.................................................................... 263
22Kan     .................................................................................................................................... 263
24Kan     .................................................................................................................................... 263
9Kan      .................................................................................................................................... 263
20Kan     ............I..................................................P.................................................................... 263
12Kan     ............I....................................................................................................................... 263
17Kan     ............I....................................................................................................................... 263
1Kan      ...............................................................P.................................................................... 263
3Kan      ...............................................................P.................................................................... 263
5USA      .................................................................................................................................... 263
21Pak     .................................................................................................................................... 263
11Pak     ......................................................................K............................................................. 263
19Pak     .................................................................................................................................... 263
12Pak     .............................................................T...................................................................... 263
5Kan      .................................................................................................................................... 263
6USA      .................................................................................................................................... 263
7USA      .................................................................................................................................... 263
4USA      .................................................................................................................................... 263
10USA     .................................................................................................................................... 263
15USA     .................................................................................................................................... 263
3USA      .................................................................................................................................... 263
8USA      .................................................................................................................................... 263
12USA     .................................................................................................................................... 263
13USA     .................................................................................................................................... 263
1USA      .................................................................................................................................... 263
2USA      .................................................................................................................................... 263
14Pak     .................................................................................................................................... 263
15Pak     .................................................................................................................................... 263
17Pak     .................................................................................................................................... 263
18Pak     .................................................................................................................................... 263
22Pak     .................................................................................................................................... 263
23Pak     .................................................................................................................................... 263
10Kan     .................................................................................................................................... 263
7Kan      .................................................................................................................................... 263
```

```
           ------------------------------------------------------------------------Capsid-NTD----------------------P3--------------------------
                 140       150       160       170       180       190       200       210       220       230       240       250       260
           --:----|----:----|----:----|----:----|----:----|----:----|----:----|----:----|----:----|----:----|----:----|----:----|----:----|----
8Kan       ....................................................................................................................................   263
33USA      ....................................................................................................................................   263
14Kan      ....................................................................................................................................   263
16Kan      ....................................................................................................................................   263
2Kan       ....................................................................................................................................   263
30P        .............................................................T......................................................................   263
4Ž         ....................................................................................................................................   263
7K         ............I.......................................................................................................................   263
20K        .....S..............................................................................................................................   263
1M         ....................................................................................................................................   263
15K        ....................................................................................................................................   263
11Ukr      ....................................................................................................................................   263
14M        ....................................................................................................................................   263
14K        ....................................................................................................................................   263
9K         ....................................................................................................................................   263
10K        ....................................................................................................................................   263
11K        ....................................................................................................................................   263
12K        ....................................................................................................................................   263
2Pak       ......................................................................................................................T...I.........   263
1Pak       ......................................................................................................................T...I.........   263
3Pak       ......................................................................................................................T...I.........   263
4Pak       ......................................................................................................................T...I.........   263
5Pak       ......................................................................................................................T...I.........   263
6Pak       ......................................................................................................................T...I.........   263
7Pak       ......................................................................................................................T...I.........   263
8Pak       ......................................................................................................................T...I.........   263
9Pak       ......................................................................................................................T...I.........   263
1K         ....................................................................................................................................   263
2K         ....................................................................................................................................   263
24K        .........................................................T..........................................................................   263
23K        .........................................................T..........................................................................   263
3Ž         .........................................................T..........................................................................   263
8M         ....................................................................................................................................   264
2M         ....................................................................................................................................   264
5M         ....................................................................................................................................   264
4K         ....................................................................................................................................   264
5K         ....................................................................................................................................   264
16M        ....................................................................................................................................   264
10M        ....................................................................................................................................   264
11M        ....................................................................................................................................   264
12M        ....................................................................................................................................   264
9M         ....................................................................................................................................   264
6M         ....................................................................................................................................   264
7M         ....................................................................................................................................   264
3P         ....................................................................................................................................   264
3M         ....................................................................................................................................   264
5P         ....................................................................................................................................   264
7P         ....................................................................................................................................   263
4P         ....................................................................................................................................   263
17P        ....................................................................................................................................   263
22K        ....................................................................................................................................   263
12P        ....................................................................................................................................   263
4M         ....................................................................................................................................   263
18K        ....................................................................................................................................   263
19K        ....................................................................................................................................   263
14P        ....................................................................................................................................   263
15P        ....................................................................................................................................   263
27P        ....................................................................................................................................   263
8P         ....................................................................................................................................   263
17K        ....................................................................................................................................   263
```

```
          Capsid-CTD                                      P4                  Nucleocapsid                      P5
          270       280       290       300       310       320       330       340       350       360       370       380       390
Consensus LADNLPDGVPKEPIIDSLSYANANKECQQILQGRGLVAAPVGQKLQACAHWAPKIKQPAILVHTPGPKMPGPRQPAPKRPPPGPCYRCLKEGHWARDCPTKTTGPPPGPCPICKDPSHWKRDCPTLKSKN*  394
1P        ......................................................M............................................................................  394
28P       ...........D.............D............................M............................................................................  394
2P        ......................................................M............................................................................  394
21P       ......................................................M............................................................................  394
18P       ......................................................M............................................................................  394
23P       ......................................................M............................................................................  394
16P       ......................................................M............................................................................  394
19P       ......................................................M............................................................................  394
24P       ......................................................M............................................................................  394
6P        ......................................................M............................................................................  394
11Kan     ......................................................M..............................................A.............................  394
15Kan     ......................................................M..............................................A.............................  394
10Pak     ......................................................M....V.........................................A.............................  394
17USA     ......................................................M....V.........................................A.............................  394
26USA     ......................................................M....V.........................................A.............................  394
27USA     ......................................................M....V.........................................A.............................  394
30USA     ......................................................M....V.........................................A.............................  394
24USA     ......................................................M....V.........................................A.............................  394
4Kan      ......................................................M..............................................A.............................  394
16USA     ......................................................M..............................................A.............................  394
18Kan     ......................................................M.....................S........................A.............................  394
13Kan     ......................................................M..............................................A.............................  394
18USA     ......................................................M..............................................A.............................  394
20USA     ......................................................M..............................................A.............................  394
28USA     ......................................................M..............................................A.............................  394
31USA     ......................................................M..............................................A.............................  394
29USA     ......................................................M..............................................A.............................  394
19Kan     ......................................................V....V.........................................A.............................  394
23Kan     ......................................................V....V.........................................A.............................  394
25Kan     ......................................................V....V.........................................A.............................  394
21Kan     ......................................................V....V.........................................A.............................  394
22Kan     ......................................................V....V.........................................A.............................  394
24Kan     ......................................................V....V.........................................A.............................  394
9Kan      ......................................................M........A.....................................A.............................  394
20Kan     ......................................................M..............................................A.............................  394
12Kan     ......................................................M..............................................A.............................  394
17Kan     ......................................................M..............................................A.............................  394
1Kan      ......................................................M....V.........................................A.............................  394
3Kan      ......................................................M....V.........................................A.............................  394
5USA      ......................................................M....V.........................................A.............................  394
21Pak     .....................................................................................................A.............................  394
11Pak     ......................................................M..............................................A.............................  394
19Pak     ..................G...................................M..............................................A.............................  394
12Pak     ......................................................M..............................................A.............................  394
5Kan      ......................................................M..............................................A.............................  394
6USA      ......................................................M..............................................A.............................  394
7USA      ......................................................M..............................................A.............................  394
4USA      ......................................................M..............................................A.............................  394
10USA     ......................................................M..............................................A.............................  394
15USA     ......................................................M..............................................A.............................  394
3USA      ......................................................M..............................................A.............................  394
8USA      ......................................................M..............................................A.............................  394
12USA     ......................................................M..............................................A.............................  394
13USA     ......................................................M..............................................A.............................  394
1USA      ......................................................M..............................................A.............................  394
2USA      ......................................................M..............................................A.............................  394
14Pak     ......................................................M..............................................A.............................  394
15Pak     ......................................................M..............................................A.............................  394
17Pak     ......................................................M..............................................A.............................  394
18Pak     ......................................................M..............................................A.............................  394
22Pak     ......................................................M..............................................A.............................  394
23Pak     ......................................................M..............................................A.............................  394
```

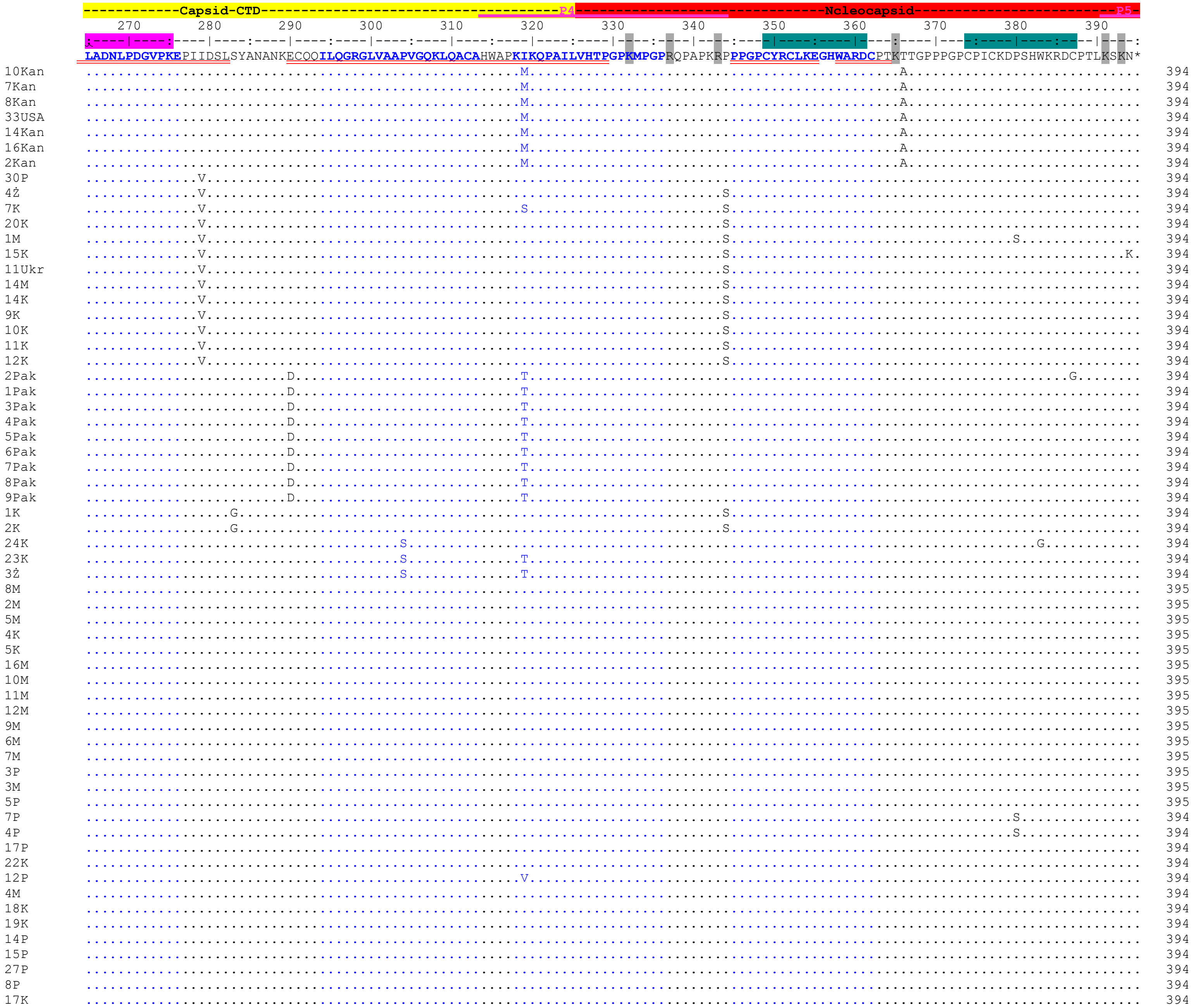
------------Capsid-CTD---P4-----------------------------------Ncleocapsid---------------------------P5-
270 280 290 300 310 320 330 340 350 360 370 380 390
LADNLPDGVPKEPIIDSLSYANANKECQQILQGRGLVAAPVGQKLQACAHWAPKIKQPAILVHTPGPKMPGPRQPAPKRPPPGPCYRCLKEGHWARDCPTKTTGPPPGPCPICKDPSHWKRDCPTLKSKN*
10Kan 394
7Kan 394
8Kan 394
33USA 394
14Kan 394
16Kan 394
2Kan 394
30P 394
4Ż 394
7K 394
20K 394
1M 394
15K 394
11Ukr 394
14M 394
14K 394
9K 394
10K 394
11K 394
12K 394
2Pak 394
1Pak 394
3Pak 394
4Pak 394
5Pak 394
6Pak 394
7Pak 394
8Pak 394
9Pak 394
1K 394
2K 394
24K 394
23K 394
3Ż 394
8M 395
2M 395
5M 395
4K 395
5K 395
16M 395
10M 395
11M 395
12M 395
9M 395
6M 395
7M 395
3P 395
3M 395
5P 395
7P 394
4P 394
17P 394
22K 394
12P 394
4M 394
18K 394
19K 394
14P 394
15P 394
27P 394
8P 394
17K 394

Figure S2. Protein sequence alignment of BLV Gag proteins performed with Clustal Omega. The consensus sequence is at the bottom. Above the consensus the sequence of the matrix, MA (shaded green), the capsid, CA (shaded yellow), the nucleocapsid, NC (shaded red), the sequence of the CD4+ T cell epitopes determined by Mager and coworkers: J Gen Virol 75 (Pt 9), 2223-2231 (1994) (shaded magenta), Zinc finger domains (shaded turquoise) and NC basic residues (shaded grey) are indicated. Below the sequence double red line mark antigenic sites, wavy black line mark major homology region (MHR). Gag consensus residues (and by dots) in blue indicate CD4+ T cell epitopes predicted in this study.

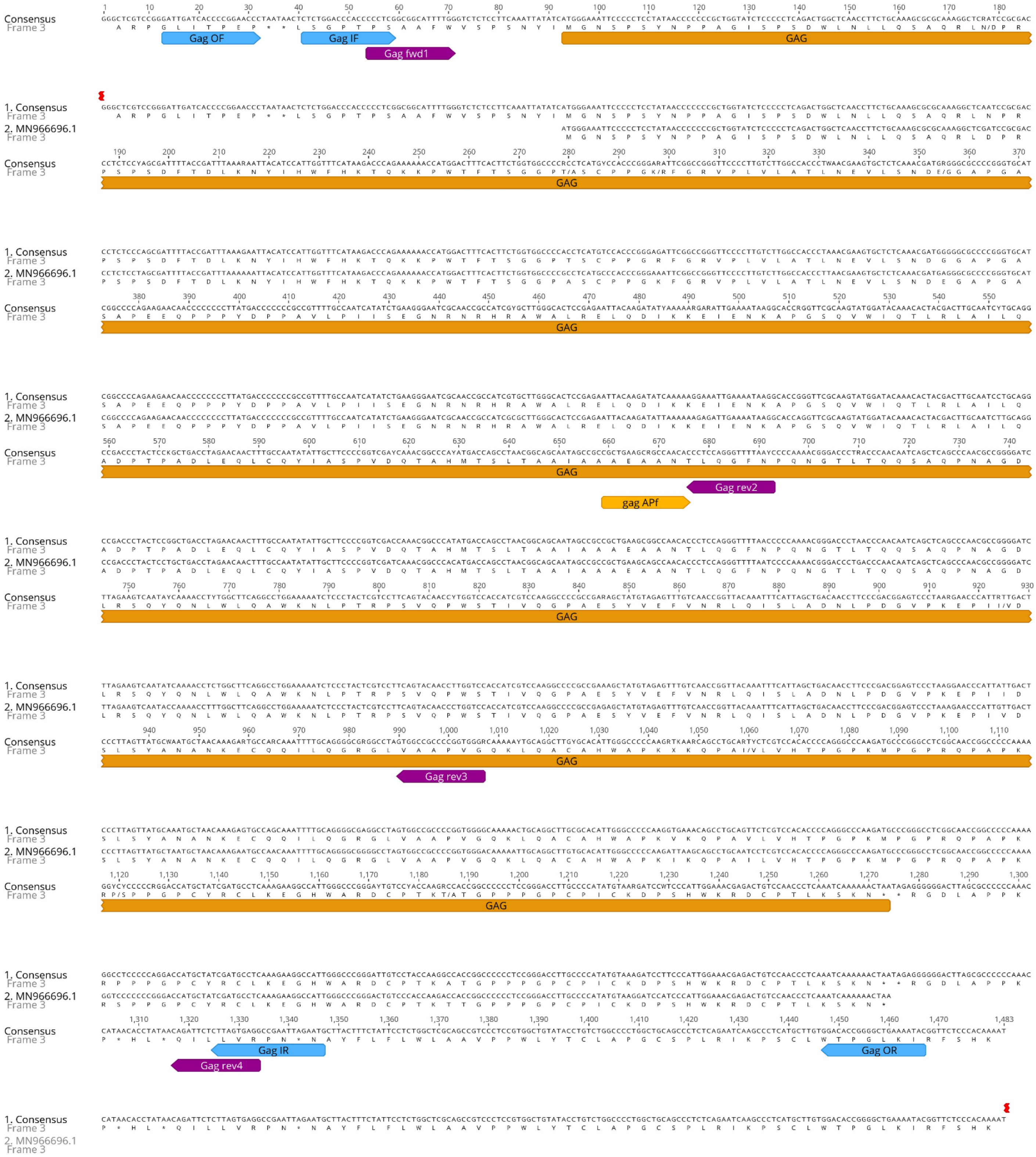

Figure S5. Map of primers within BLV provirus sequence for PCR amplification of gag gene region and sequencing of the PCR products obtained. The primers used for nested PCR are indicated by blue arrows, and the primers used for sequencing are indicated by purple and orange arrows.

e **f**

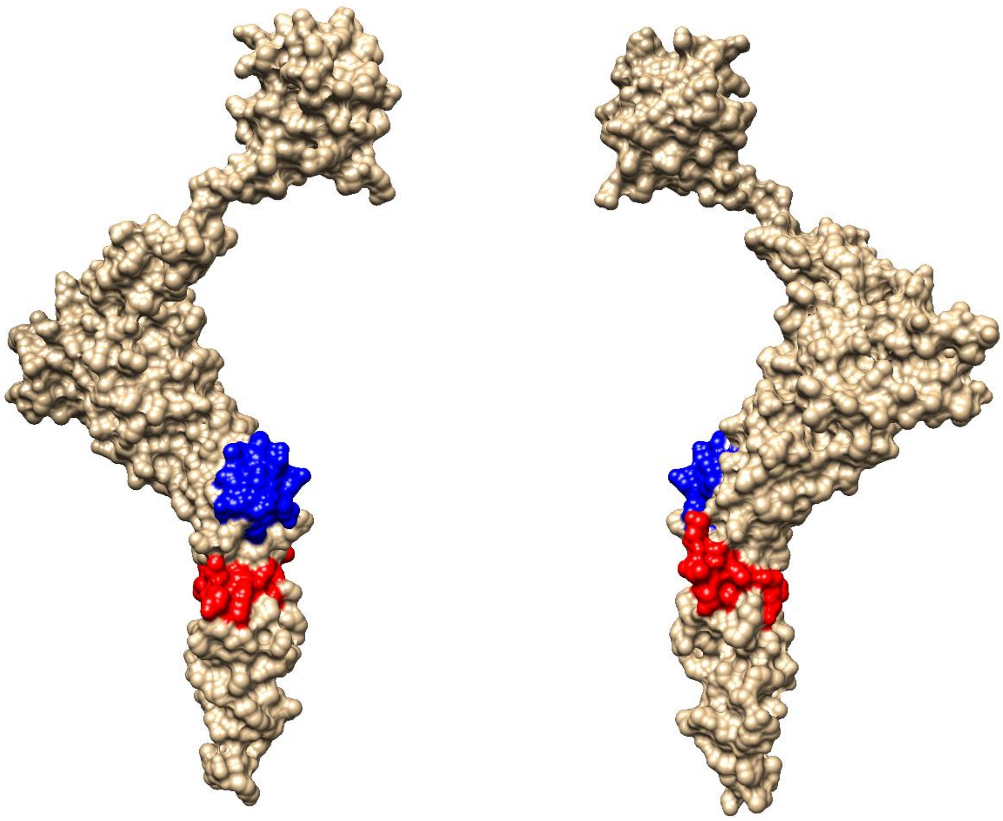

Side A **Side B**

Figure S6. Representation of the CD4+ T cell epitopes on 3D protein structure model of BLV Gag, which correlate in BLV-infected cattle with low proviral load. The model is shown as space-filled images of opposite sides arbitrarily named side A and side B. On the Gag structure epitope 1A (red) and epitope 2 (blue) are presented.

Table S2. Analysis for evidence of selection in BLV gag sequences. Sliding window analysis based on the rate of DNA polymorphism and divergence in synonymous sites and non-synonymous sites. The rate of non-synonymous substitutions (NoSyn) to the rate of synonymous substitutions (Syn) at each nucleotide position calculated using the following sliding window options: window length: 9, step size: 3 from the whole coding region.

Region (nt)	Pi (NoSyn)	Pi (Syn)	Pi (NoSyn)/Pi (Syn)	Sites of positive selection
**76-93**	0,0196	0,0415	0,4724	–
**133-147**	0,0441	0,0291	1,5153	48: H, Y, R
**175-195**	0,0778	0,0255	3,0478	61: G, S, D
				63: V, T, A
**199-216**	0,0546	0,0417	1,3109	69: R, K
**250-279**	0,080395	0,0713	1,1278	87: D, E
				88: G, E
**316-342**	0,0316	0,0261	1,212	109: V, I
				110: L, M
				113: I, V
**829-843**	0,0203	0,0060	3,3833	279: I, V
**862-879**	0,01574	0,33290	0,0473	–
**952-972**	0,0796	0,0775	1,0271	319: T, V, M, I, S
				324: I, V
**1018-1038**	0,0224	0,0834	0,2686	–
**1087-1107**	0,0573	0,054	1,0556	366: T, A

Table S5. BLV detection results examined using qPCR.

BLV isolate ID	BoLA-DRB3 genotype		Group A or B	BLV proviral copy number per 100,000 cells
**1Kan**	010:01	011:01	A	34,57
**2Kan**	010:01	011:01	A	25,71
**3Kan**	001:01	107:01:00	B	6,88
**4Kan**	015:01	001:01	A	16,68
**5Kan**	015:01	011:01	A	15,86
**7Kan**	015:01	044:01	A	23,35
**8Kan**	015:01	005:03	A	15,14
**9Kan**	027:03	010:01	A	103,71
**10Kan**	005:08	024:33	B	117,38
**11Kan**	015:01	010:01	A	26,02
**12Kan**	015:01	015:01	A	70,83
**13Kan**	015:01	015:01	A	136,68
**14Kan**	105:02:00	001:01	A	26,67
**15Kan**	012:01	114:01:00	A	82,56
**16Kan**	010:01	011:01	A	59,12
**17Kan**	015:01	010:01	A	71,87
**18Kan**	010:01	15:01	A	41,35
**19Kan**	001:01	112:02:00	B	93,64
**20Kan**	015:01	007:01	A	45,74
**21Kan**	015:01	011:01	A	61,66
**22Kan**	024:15	027:18	A	169,24
**23Kan**	015:01	012:01	A	59,01

**24Kan**	105:02:00	001:01	A	14,17
**25Kan**	105:02:00	001:01	A	174,19
**1Pak**	080:01	139:01:00	A	565,44
**2Pak**	116:01:00	015:01	A	631,5
**3Pak**	057:02	043:03	B	266,17
**4Pak**	057:02	005:02	A	103,59
**5Pak**	057:02	107:01:00	B	40,76
**6Pak**	057:02	005:02	A	267,93
**7Pak**	018:01	086:03	B	149,21
**8Pak**	001:01	024:33	B	88,86
**9Pak**	009:02	107:04:00	B	210,03
**10Pak**	012:01	010:01	A	826,02
**11Pak**	010:01	081:01	A	832,73
**12Pak**	008:01	011:01	A	953,91
**14Pak**	010:01	010:01	A	1,046,14
**15Pak**	011:01	015:04	A	11,31
**17Pak**	025:01:01	044:01	A	2,011,91
**18Pak**	002:01	020:01:01	A	838,08
**19Pak**	086:02	044:01	A	205,9
**21Pak**	025:01:01	018:01	B	144,01
**22Pak**	001:01	nd	NA	881,88
**23Pak**	001:01	024:33	B	38,73
**1M**	012:01	nd	A	7,91
**2M**	020:01:01	028:01	A	0,07
**3M**	018:01	105:02:00	A	12,38
**4M**	005:04	019:02	A	410,39

**5M**	008:01	011:02	A	0,92
**6M**	002:01	010:01	A	15,67
**7M**	012:01	010:01	A	31,33
**8M**	001:01	010:01	A	29,07
**9M**	001:01	015:05	A	0,01
**10M**	014:01:01	001:01	B	5,57
**11M**	014:03	075:03	B	0,06
**12M**	001:01	nd	NA	0,03
**14M**	010:01	nd	A	0,001
**16M**	007:01	024:32	B	0,02
**1P**	011:01	001:01	A	103,6
**2P**	011:01	018:01	A	0,06
**3P**	001:01	024:33	B	0,05
**4P**	011:01	009:04	A	0,02
**5P**	028:05	004:01	B	157,45
**6P**	018:01	025:01:01	B	14,84
**7P**	001:01	001:01	B	2,42
**8P**	009:02	009:02	B	0,05
**12P**	010:01	035:01	A	36,62
**14P**	011:01	001:01	A	137,5
**15P**	027:03	027:03	B	20,894
**16P**	038:01	nd	NA	142,06
**17P**	015:01	025:01:01	A	81,33
**18P**	009:01	012:01	A	38,29
**19P**	012:03	142:01:00	A	308,69
**20P**	014:01:01	014:01:01	B	144,93

**23P**	024:03	nd	NA	0,03
**24P**	014:01:01	014:04	B	1,2
**27P**	016:01	010:01	A	0,34
**28P**	011:01	009:04	A	0,02
**30P**	006:01	141:01:00	B	0,97
**1K**	013:01	130:01:00	B	18,13
**2K**	013:01	015:04	B	35,33
**4K**	017:01	009:01	A	273,2
**5K**	017:01	009:01	A	241,93
**7K**	027:03	139:01:00	A	606,84
**9K**	009:02	107:04:00	B	102,45
**10K**	041:01	157:01:00	A	367,7
**11K**	041:01	nd	A	284,45
**12K**	041:01	041:01	A	273,16
**14K**	001:01	nd	NA	475,26
**15K**	041:01	nd	A	364,7
**17K**	007:01	011:01	A	0,88
**18K**	032:01	005:07	A	267,71
**19K**	027:13	007:01	B	10,95
**20K**	027:03	024:03	B	5,2
**22K**	003:01:01	004:01	B	33,14
**23K**	045:01	134:01:00	B	0,002
**24K**	001:01	007:01	B	0,002
**3Ż**	011:01	116:01:00	A	0,41
**4Ż/2**	001:01	112:03:00	B	481,84
**11UKR**	005:03	134:01:00	B	0,06

**10USA**	001:01	010:01	A	16,89
**12USA**	011:01	010:03	A	9,84
**13USA**	010:01	010:04	A	12,74
**15USA**	011:01	116:01:00	A	18,16
**16USA**	001:01	160:01:00	B	19,43
**17USA**	001:01	160:01:00	B	15,09
**18USA**	001:01	014:01:01	B	15,08
**1USA**	011:01	116:01:00	A	12,96
**20USA**	011:01	015:01	A	32,61
**24USA**	015:01	015:01	A	25,84
**26USA**	001:01	130:01:00	B	28,64
**27USA**	001:01	001:01	B	16,62
**28USA**	001:01	011:01	A	25,87
**29USA**	001:01	011:01	A	16,55
**2USA**	001:01	160:01:00	B	0,31
**30USA**	031:01	089:01	B	21,87
**31USA**	001:01	160:01:00	B	13,49
**33USA**	012:01	012:01	A	11,55
**3USA**	001:01	014:01:01	B	0,77
**4USA**	012:01	142:01:00	A	46,92
**5USA**	001:01	031:01	B	0,67
**6USA**	001:01	nd	NA	16,46
**7USA**	001:01	027:03	B	6,73
**8USA**	001:01	nd	NA	15,19

Abbreviations: nd- not determined; NA- not assigned
