# Supplementary material for: An immunoinformatics study reveals a new BoLA-DR-restricted CD4+ T cell epitopes on the Gag protein of bovine leukemia virus
Source: Sci Rep. 2023 Dec 15;13:22356. doi: 10.1038/s41598-023-48899-4 (PMC10724172; doi:10.1038/s41598-023-48899-4)
Supplement: Supplementary file 8 — Supplementary Table S6. [file 41598_2023_48899_MOESM8_ESM.docx]

| **Supplementary Table S6.**  Changes in the amino acid sequence of the Gag peptides that alter the binding affinity of the BoLA-DRB3 alleles.  Amino acid changes, which generate new BoLA-DRB3 binding affinity site are shown in red; changes that impair BoLA-DRB3 binding affinity sites are colored green; changes that enhance BoLA-DRB3 affinity are marked in orange, mutations that generate lack of peptides interactions with BoLA-DRB3 are marked in grey. | | | | | |
| --- | --- | --- | --- | --- | --- |
| **Amino acid change** | **Isolate/** | **BoLA-DRB3*/Isolate** | **Epitope/** | **Effect of the amino** | **The peptide interaction** |
| **/Peptide position** | **the peptide sequence** |  | **Core sequence** | **acid substitution/Rank %** | **with BoLA-DRB3** |
| N29D | 20K | 024:03 | Epitope 15A | 1NB --> 1WB | The presence of a new binding site for BoLA-DRB3 |
| Peptide position | Peptide | Gag consensus/Isolate | Core | Rank % | Binding affinity predictor |
| 15 | PSDWLNLLQSAQRL**N** | Consensus | LNLLQSAQR | 7.33 | 0 |
| 16 | SDWLNLLQSAQRL**N**P | Consensus | LNLLQSAQR | 5.62 | NB |
|  |  |  |  |  |  |
| 15 | PSDWLNLLQSAQRL**D** | 20K | LNLLQSAQR | 5.73 | 0 |
| 16 | SDWLNLLQSAQRL**D**P | 20K | LNLLQSAQR | 4.38 | WB |
|  |  |  |  |  |  |
| K41R | 9Kan | 010:01 | Epitope 4A | 3SB, 1WB --> 2SB, 2WB | Weakened binding affinity of Epitope to BoLA-DRB3 |
| Position | Peptide | Gag consensus/Isolate | Core | Rank % | Binding affinity predictor |
| 38 | TDL**K**NYIHWFHKTQK | Consensus | YIHWFHKTQ | 1.77 | WB |
| 39 | DL**K**NYIHWFHKTQKK | Consensus | YIHWFHKTQ | 0.95 | SB |
| 40 | L**K**NYIHWFHKTQKKP | Consensus | YIHWFHKTQ | 0.66 | SB |
| 41 | **K**NYIHWFHKTQKKPW | Consensus | YIHWFHKTQ | 0.83 | SB |
|  |  |  |  |  |  |
| 38 | TDL**R**NYIHWFHKTQK | 9Kan | YIHWFHKTQ | 1.99 | WB |
| 39 | DL**R**NYIHWFHKTQKK | 9Kan | YIHWFHKTQ | 1.09 | WB |
| 40 | L**R**NYIHWFHKTQKKP | 9Kan | YIHWFHKTQ | 0.76 | SB |
| 41 | **R**NYIHWFHKTQKKPW | 9Kan | IHWFHKTQK | 0.93 | SB |
|  |  |  |  |  |  |
| H48R | 1K | 130:01:00 | Epitope 6 | 3WB --> 1SB, 2WB | Strengthened binding affinity of Epitope to BoLA-DRB3 |
| Position | Peptide | Gag consensus/Isolate | Core | Rank % | Binding affinity predictor |
| 42 | NYIHWF**H**KTQKKPWT | Consensus | FHKTQKKPW | 1.18 | WB |
| 43 | YIHWF**H**KTQKKPWTF | Consensus | FHKTQKKPW | 1.77 | WB |
| 44 | IHWF**H**KTQKKPWTFT | Consensus | FHKTQKKPW | 2.86 | WB |
| 45 | HWF**H**KTQKKPWTFTS | Consensus | FHKTQKKPW | 7.08 | 0 |
|  |  |  |  |  |  |
| 42 | NYIHWF**R**KTQKKPWT | 1K | FRKTQKKPW | 0.86 | SB |
| 43 | YIHWF**R**KTQKKPWTF | 1K | FRKTQKKPW | 1.21 | WB |
| 44 | IHWF**R**KTQKKPWTFT | 1K | FRKTQKKPW | 1.76 | WB |
| 45 | HWF**R**KTQKKPWTFTS | 1K | FRKTQKKPW | 5.49 | 0 |
|  |  |  |  |  |  |
| H48Y | 3Pak | 043:03 | Epitope 4A | 3WB --> 2WB, 1NB | Weakened binding affinity of Epitope to BoLA-DRB3 |
| Position | Peptide | Gag consensus/Isolate | Core | Rank % | Binding affinity predictor |
| 38 | TDLKNYIHWF**H**KTQK | Consensus | YIHWFHKTQ | 6.62 | 0 |
| 39 | DLKNYIHWF**H**KTQKK | Consensus | IHWFHKTQK | 3.18 | WB |
| 40 | LKNYIHWF**H**KTQKKP | Consensus | IHWFHKTQK | 1.79 | WB |
| 41 | KNYIHWF**H**KTQKKPW | Consensus | IHWFHKTQK | 1.54 | WB |
|  |  |  |  |  |  |
|  |  |  |  |  |  |
| 38 | TDLKNYIHWF**Y**KTQK | 3Pak | IHWFYKTQK | 14.47 | 0 |
| 39 | DLKNYIHWF**Y**KTQKK | 3Pak | IHWFYKTQK | 7.71 | NB |
| 40 | LKNYIHWF**Y**KTQKKP | 3Pak | IHWFYKTQK | 4.60 | WB |
| 41 | KNYIHWF**Y**KTQKKPW | 3Pak | IHWFYKTQK | 2.94 | WB |
|  |  |  |  |  |  |
| H48R | 2K | 015:04 | Epitope 4A | 2NB --> 2WB | The presence of a new binding site for the BoLA-DRB3 |
| Position | Peptide | Gag consensus/Isolate | Core | Rank % | Binding affinity predictor |
| 38 | TDLKNYIHWF**H**KTQK | Consensus | IHWFHKTQK | 13.77 | 0 |
| 39 | DLKNYIHWF**H**KTQKK | Consensus | IHWFHKTQK | 10.18 | 0 |
| 40 | LKNYIHWF**H**KTQKKP | Consensus | IHWFHKTQK | 6.90 | NB |
| 41 | KNYIHWF**H**KTQKKPW | Consensus | IHWFHKTQK | 6.46 | NB |
|  |  |  |  |  |  |
| 38 | TDLKNYIHWF**R**KTQK | 2K | IHWFRKTQK | 13.77 | 0 |
| 39 | DLKNYIHWF**R**KTQKK | 2K | IHWFRKTQK | 6.47 | 0 |
| 40 | LKNYIHWF**R**KTQKKP | 2K | IHWFRKTQK | 4.15 | WB |
| 41 | KNYIHWF**R**KTQKKPW | 2K | IHWFRKTQK | 4.41 | WB |
|  |  |  |  |  |  |
|  |  |  |  |  |  |
| H48R | 2K | 015:04 | Epitope 6 | 1NB --> 1WB | The presence of a new binding site for the BoLA-DRB3 |
| Position | Peptide | Gag consensus/Isolate | Core | Rank % | Binding affinity predictor |
| 42 | NYIHWF**H**KTQKKPWT | Consensus | FHKTQKKPW | 6.80 | NB |
| 43 | YIHWF**H**KTQKKPWTF | Consensus | FHKTQKKPW | 9.02 | 0 |
| 44 | IHWF**H**KTQKKPWTFT | Consensus | FHKTQKKPW | 10.65 | 0 |
| 45 | HWF**H**KTQKKPWTFTS | Consensus | FHKTQKKPW | 15.96 | 0 |
|  |  |  |  |  |  |
| 42 | NYIHWF**R**KTQKKPWT | 2K | FRKTQKKPW | 4.77 | WB |
| 43 | YIHWF**R**KTQKKPWTF | 2K | FRKTQKKPW | 7.80 | 0 |
| 44 | IHWF**R**KTQKKPWTFT | 2K | FRKTQKKPW | 9.11 | 0 |
| 45 | HWF**R**KTQKKPWTFTS | 2K | FRKTQKKPW | 16.54 | 0 |
|  |  |  |  |  |  |
| H48Y | 3Pak | 043:03 | Epitope 6 | 2WB, 1NB --> 3WB | Strengthened binding affinity of Epitope to BoLA-DRB3 |
| Position | Peptide | Gag consensus/Isolate | Core | Rank % | Binding affinity predictor |
| 42 | NYIHWF**H**KTQKKPWT | Consensus | FHKTQKKPW | 2.05 | WB |
| 43 | YIHWF**H**KTQKKPWTF | Consensus | FHKTQKKPW | 3.79 | WB |
| 44 | IHWF**H**KTQKKPWTFT | Consensus | FHKTQKKPW | 5.01 | NB |
| 45 | HWF**H**KTQKKPWTFTS | Consensus | FHKTQKKPW | 9.72 | 0 |
|  |  |  |  |  |  |
| 42 | NYIHWF**Y**KTQKKPWT | 3Pak | FYKTQKKPW | 3.11 | WB |
| 43 | YIHWF**Y**KTQKKPWTF | 3Pak | FYKTQKKPW | 4.06 | WB |
| 44 | IHWF**Y**KTQKKPWTFT | 3Pak | FYKTQKKPW | 4.93 | WB |
| 45 | HWF**Y**KTQKKPWTFTS | 3Pak | FYKTQKKPW | 9.07 | 0 |
|  |  |  |  |  |  |
| H48Y | 7Pak | 018:01 | Epitope 4A | 2SB, 2WB --> 2SB, 1WB, 1NB | Weakened binding affinity of Epitope to BoLA-DRB3 |
| Position | Peptide | Gag consensus/Isolate | Core | Rank % | Binding affinity predictor |
| 38 | TDLKNYIHWF**H**KTQK | Consensus | IHWFHKTQK | 3.49 | WB |
| 39 | DLKNYIHWF**H**KTQKK | Consensus | IHWFHKTQK | 1.07 | WB |
| 40 | LKNYIHWF**H**KTQKKP | Consensus | IHWFHKTQK | 0.64 | SB |
| 41 | KNYIHWF**H**KTQKKPW | Consensus | IHWFHKTQK | 0.69 | SB |
|  |  |  |  |  |  |
|  |  |  |  |  |  |
| 38 | TDLKNYIHWF**Y**KTQK | 7Pak | IHWFYKTQK | 5.22 | NB |
| 39 | DLKNYIHWF**Y**KTQKK | 7Pak | IHWFYKTQK | 1.18 | WB |
| 40 | LKNYIHWF**Y**KTQKKP | 7Pak | IHWFYKTQK | 0.49 | SB |
| 41 | KNYIHWF**Y**KTQKKPW | 7Pak | IHWFYKTQK | 0.65 | SB |
|  |  |  |  |  |  |
|  |  |  |  |  |  |
| H48Y | 7Pak | 018:01 | Epitope 6 | 2WB --> 1WB, 1NB | Weakened binding affinity of Epitope to BoLA-DRB3 |
| Position | Peptide | Gag consensus/Isolate | Core | Rank % | Binding affinity predictor |
| 42 | NYIHWF**H**KTQKKPWT | Consensus | IHWFHKTQK | 1.11 | WB |
| 43 | YIHWF**H**KTQKKPWTF | Consensus | FHKTQKKPW | 3.58 | WB |
| 44 | IHWF**H**KTQKKPWTFT | Consensus | FHKTQKKPW | 10.60 | 0 |
| 45 | HWF**H**KTQKKPWTFTS | Consensus | FHKTQKKPW | 21.94 | 0 |
|  |  |  |  |  |  |
| 42 | NYIHWF**Y**KTQKKPWT | 7Pak | IHWFYKTQK | 1.53 | WB |
| 43 | YIHWF**Y**KTQKKPWTF | 7Pak | FYKTQKKPW | 5.40 | NB |
| 44 | IHWF**Y**KTQKKPWTFT | 7Pak | FYKTQKKPW | 12.81 | 0 |
| 45 | HWF**Y**KTQKKPWTFTS | 7Pak | FYKTQKKPW | 24.92 | 0 |
|  |  |  |  |  |  |
| H48Y | 8Pak | 001:01 | Epitope 4A | 2SB, 2WB --> 1SB, 3WB | Weakened binding affinity of Epitope to BoLA-DRB3 |
| Position | Peptide | Gag consensus/Isolate | Core | Rank % | Binding affinity predictor |
| 38 | TDLKNYIHWF**H**KTQK | Consensus | IHWFHKTQK | 3.43 | WB |
| 39 | DLKNYIHWF**H**KTQKK | Consensus | IHWFHKTQK | 1.13 | WB |
| 40 | LKNYIHWF**H**KTQKKP | Consensus | IHWFHKTQK | 0.67 | SB |
| 41 | KNYIHWF**H**KTQKKPW | Consensus | IHWFHKTQK | 0.72 | SB |
|  |  |  |  |  |  |
| 38 | TDLKNYIHWF**Y**KTQK | 8Pak | IHWFYKTQK | 4.25 | WB |
| 39 | DLKNYIHWF**Y**KTQKK | 8Pak | IHWFYKTQK | 1.41 | WB |
| 40 | LKNYIHWF**Y**KTQKKP | 8Pak | IHWFYKTQK | 0.88 | SB |
| 41 | KNYIHWF**Y**KTQKKPW | 8Pak | IHWFYKTQK | 1.01 | WB |
|  |  |  |  |  |  |
| H48Y | 3M | 018:01 | Epitope 4A | 2SB, 3WB --> 2SB, 1WB, 1NB | Weakened binding affinity of Epitope to BoLA-DRB3 |
| Position | Peptide | Gag consensus/Isolate | Core | Rank % | Binding affinity predictor |
| 38 | TDLKNYIHWF**H**KTQK | Consensus | IHWFHKTQK | 3.49 | WB |
| 39 | DLKNYIHWF**H**KTQKK | Consensus | IHWFHKTQK | 1.07 | WB |
| 40 | LKNYIHWF**H**KTQKKP | Consensus | IHWFHKTQK | 0.64 | SB |
| 41 | KNYIHWF**H**KTQKKPW | Consensus | IHWFHKTQK | 0.69 | SB |
|  |  |  |  |  |  |
| 38 | TDLKNYIHWF**Y**KTQK | 3M | IHWFYKTQK | 5.22 | NB |
| 39 | DLKNYIHWF**Y**KTQKK | 3M | IHWFYKTQK | 1.18 | WB |
| 40 | LKNYIHWF**Y**KTQKKP | 3M | IHWFYKTQK | 0.49 | SB |
| 41 | KNYIHWF**Y**KTQKKPW | 3M | IHWFYKTQK | 0.65 | SB |
|  |  |  |  |  |  |
| H48Y | 5M | 008:01 | Epitope 4A | 1SB, 2WB --> 3WB | Weakened binding affinity of Epitope to BoLA-DRB3 |
| Position | Peptide | Gag consensus/Isolate | Core | Rank % | Binding affinity predictor |
| 38 | TDLKNYIHWF**H**KTQK | Consensus | IHWFHKTQK | 10.07 | 0 |
| 39 | DLKNYIHWF**H**KTQKK | Consensus | IHWFHKTQK | 2.61 | WB |
| 40 | LKNYIHWF**H**KTQKKP | Consensus | IHWFHKTQK | 1.08 | WB |
| 41 | KNYIHWF**H**KTQKKPW | Consensus | IHWFHKTQK | 0.90 | SB |
|  |  |  |  |  |  |
| 38 | TDLKNYIHWF**Y**KTQK | 5M | IHWFYKTQK | 10.83 | 0 |
| 39 | DLKNYIHWF**Y**KTQKK | 5M | IHWFYKTQK | 3.47 | WB |
| 40 | LKNYIHWF**Y**KTQKKP | 5M | IHWFYKTQK | 1.33 | WB |
| 41 | KNYIHWF**Y**KTQKKPW | 5M | IHWFYKTQK | 1.21 | WB |
|  |  |  |  |  |  |
| H48Y | 6M | 010:01 | Epitope 4A | 3SB, 1WB --> 4WB | Weakened binding affinity of Epitope to BoLA-DRB3 |
| H48Y | 7M | 010:01 | Epitope 4A | 3SB, 1WB --> 4WB | Weakened binding affinity of Epitope to BoLA-DRB3 |
| H48Y | 8M | 010:01 | Epitope 4A | 3SB, 1WB --> 4WB | Weakened binding affinity of Epitope to BoLA-DRB3 |
| Position | Peptide | Gag consensus/Isolate | Core | Rank % | Binding affinity predictor |
| 38 | TDLKNYIHWF**H**KTQK | Consensus | YIHWFHKTQ | 1.77 | WB |
| 39 | DLKNYIHWF**H**KTQKK | Consensus | YIHWFHKTQ | 0.95 | SB |
| 40 | LKNYIHWF**H**KTQKKP | Consensus | YIHWFHKTQ | 0.66 | SB |
| 41 | KNYIHWF**H**KTQKKPW | Consensus | YIHWFHKTQ | 0.83 | SB |
|  |  |  |  |  |  |
|  |  |  |  |  |  |
| 38 | TDLKNYIHWF**Y**KTQK | 6M | IHWFYKTQK | 4.56 | WB |
| 39 | DLKNYIHWF**Y**KTQKK | 6M | IHWFYKTQK | 2.30 | WB |
| 40 | LKNYIHWF**Y**KTQKKP | 6M | IHWFYKTQK | 1.57 | WB |
| 41 | KNYIHWF**Y**KTQKKPW | 6M | IHWFYKTQK | 1.40 | WB |
|  |  |  |  |  |  |
|  |  |  |  |  |  |
| H48Y | 8M | 001:01 | Epitope 4A | 2SB, 2WB --> 1SB, 3WB | Weakened binding affinity of Epitope to BoLA-DRB3 |
| H48Y | 9M | 001:01 | Epitope 4A | 2SB, 2WB --> 1SB, 3WB | Weakened binding affinity of Epitope to BoLA-DRB3 |
| H48Y | 10M | 001:01 | Epitope 4A | 2SB, 2WB --> 1SB, 3WB | Weakened binding affinity of Epitope to BoLA-DRB3 |
| H48Y | 12M | 001:01 | Epitope 4A | 2SB, 2WB --> 1SB, 3WB | Weakened binding affinity of Epitope to BoLA-DRB3 |
| H48Y | 3P | 001:01 | Epitope 4A | 2SB, 2WB --> 1SB, 3WB | Weakened binding affinity of Epitope to BoLA-DRB3 |
| Position | Peptide | Gag consensus/Isolate | Core | Rank % | Binding affinity predictor |
| 38 | TDLKNYIHWF**H**KTQK | Consensus | IHWFHKTQK | 3.43 | WB |
| 39 | DLKNYIHWF**H**KTQKK | Consensus | IHWFHKTQK | 1.13 | WB |
| 40 | LKNYIHWF**H**KTQKKP | Consensus | IHWFHKTQK | 0.67 | SB |
| 41 | KNYIHWF**H**KTQKKPW | Consensus | IHWFHKTQK | 0.72 | SB |
|  |  |  |  |  |  |
| 38 | TDLKNYIHWF**Y**KTQK | 8M | IHWFYKTQK | 4.25 | WB |
| 39 | DLKNYIHWF**Y**KTQKK | 8M | IHWFYKTQK | 1.41 | WB |
| 40 | LKNYIHWF**Y**KTQKKP | 8M | IHWFYKTQK | 0.88 | SB |
| 41 | KNYIHWF**Y**KTQKKPW | 8M | IHWFYKTQK | 1.01 | WB |
|  |  |  |  |  |  |
| H48Y | 11M | 014:03 | Epitope 4A | 2SB, 1WB --> 3WB | Weakened binding affinity of Epitope to BoLA-DRB3 |
| Position | Peptide | Gag consensus/Isolate | Core | Rank % | Binding affinity predictor |
| 38 | TDLKNYIHWF**H**KTQK | Consensus | IHWFHKTQK | 5.53 | 0 |
| 39 | DLKNYIHWF**H**KTQKK | Consensus | IHWFHKTQK | 1.80 | WB |
| 40 | LKNYIHWF**H**KTQKKP | Consensus | IHWFHKTQK | 0.65 | SB |
| 41 | KNYIHWF**H**KTQKKPW | Consensus | IHWFHKTQK | 0.87 | SB |
|  |  |  |  |  |  |
| 38 | TDLKNYIHWF**Y**KTQK | 11M | IHWFYKTQK | 9.76 | 0 |
| 39 | DLKNYIHWF**Y**KTQKK | 11M | IHWFYKTQK | 2.87 | WB |
| 40 | LKNYIHWF**Y**KTQKKP | 11M | IHWFYKTQK | 1.11 | WB |
| 41 | KNYIHWF**Y**KTQKKPW | 11M | IHWFYKTQK | 1.71 | WB |
|  |  |  |  |  |  |
|  |  |  |  |  |  |
| H48Y | 16M | 007:01 | Epitope 4A | 3WB --> 2WB, 1NB | Weakened binding affinity of Epitope to BoLA-DRB3 |
| Position | Peptide | Gag consensus/Isolate | Core | Rank % | Binding affinity predictor |
| 38 | TDLKNYIHWF**H**KTQK | Consensus | IHWFHKTQK | 10.22 | 0 |
| 39 | DLKNYIHWF**H**KTQKK | Consensus | IHWFHKTQK | 3.90 | WB |
| 40 | LKNYIHWF**H**KTQKKP | Consensus | IHWFHKTQK | 1.77 | WB |
| 41 | KNYIHWF**H**KTQKKPW | Consensus | IHWFHKTQK | 2.28 | WB |
|  |  |  |  |  |  |
| 38 | TDLRNYIHWF**Y**KTQK | 16M | IHWFYKTQK | 16.31 | 0 |
| 39 | DLRNYIHWF**Y**KTQKK | 16M | IHWFYKTQK | 5.47 | NB |
| 40 | LRNYIHWF**Y**KTQKKP | 16M | IHWFYKTQK | 3.52 | WB |
| 41 | RNYIHWF**Y**KTQKKPW | 16M | IHWFYKTQK | 3.97 | WB |
|  |  |  |  |  |  |
| H48Y | 5P | 004:01 | Epitope 4A | 3SB --> 1SB, 2WB | Weakened binding affinity of Epitope to BoLA-DRB3 |
| Position | Peptide | Gag consensus/Isolate | Core | Rank % | Binding affinity predictor |
| 38 | TDLKNYIHWF**H**KTQK | Consensus | IHWFHKTQK | 5.37 | 0 |
| 39 | DLKNYIHWF**H**KTQKK | Consensus | IHWFHKTQK | 0.75 | SB |
| 40 | LKNYIHWF**H**KTQKKP | Consensus | IHWFHKTQK | 0.19 | SB |
| 41 | KNYIHWF**H**KTQKKPW | Consensus | IHWFHKTQK | 0.19 | SB |
|  |  |  |  |  |  |
| 38 | TDLKNYIHWF**Y**KTQK | 5P | IHWFYKTQK | 6.67 | 0 |
| 39 | DLKNYIHWF**Y**KTQKK | 5P | IHWFYKTQK | 1.57 | WB |
| 40 | LKNYIHWF**Y**KTQKKP | 5P | IHWFYKTQK | 0.52 | SB |
| 41 | KNYIHWF**Y**KTQKKPW | 5P | IHWFYKTQK | 0.65 | SB |
|  |  |  |  |  |  |
| H48Y | 8P | 009:02 | Epitope 4A | 1WB --> 1NB | Lack of peptide interaction with BoLA-DRB3 |
| Position | Peptide | Gag consensus/Isolate | Core | Rank % | Binding affinity predictor |
| 38 | TDLKNYIHWF**H**KTQK | Consensus | IHWFHKTQK | 16.53 | 0 |
| 39 | DLKNYIHWF**H**KTQKK | Consensus | IHWFHKTQK | 7.63 | 0 |
| 40 | LKNYIHWF**H**KTQKKP | Consensus | IHWFHKTQK | 4.43 | WB |
| 41 | KNYIHWF**H**KTQKKPW | Consensus | IHWFHKTQK | 5.37 | 0 |
|  |  |  |  |  |  |
| 38 | TDLKNYIHWF**Y**KTQK | 8P | IHWFYKTQK | 25.42 | 0 |
| 39 | DLKNYIHWF**Y**KTQKK | 8P | IHWFYKTQK | 13.86 | 0 |
| 40 | LKNYIHWF**Y**KTQKKP | 8P | IHWFYKTQK | 8.38 | NB |
| 41 | KNYIHWF**Y**KTQKKPW | 8P | IHWFYKTQK | 8.97 | 0 |
|  |  |  |  |  |  |
|  |  |  |  |  |  |
| H48Y | 4K | 009:01 | Epitope 4A | 2WB --> 2NB | Lack of peptides interactions with BoLA-DRB3 |
| H48Y | 5K | 009:01 | Epitope 4A | 2WB --> 2NB | Lack of peptides interactions with BoLA-DRB3 |
| Position | Peptide | Gag consensus/Isolate | Core | Rank % | Binding affinity predictor |
| 38 | TDLKNYIHWF**H**KTQK | Consensus | IHWFHKTQK | 15.81 | 0 |
| 39 | DLKNYIHWF**H**KTQKK | Consensus | IHWFHKTQK | 5.87 | 0 |
| 40 | LKNYIHWF**H**KTQKKP | Consensus | IHWFHKTQK | 3.19 | WB |
| 41 | KNYIHWF**H**KTQKKPW | Consensus | IHWFHKTQK | 3.52 | WB |
|  |  |  |  |  |  |
| 38 | TDLKNYIHWF**Y**KTQK | 4K | IHWFYKTQK | 19.09 | 0 |
| 39 | DLKNYIHWF**Y**KTQKK | 4K | IHWFYKTQK | 10.55 | 0 |
| 40 | LKNYIHWF**Y**KTQKKP | 4K | IHWFYKTQK | 5.48 | NB |
| 41 | KNYIHWF**Y**KTQKKPW | 4K | IHWFYKTQK | 6.49 | NB |
|  |  |  |  |  |  |
| H48Y | 7Pak | 086:03 | Epitope 4A | 3WB --> 2WB, 1NB | Weakened binding affinity of Epitope to BoLA-DRB3 |
| Position | Peptide | Gag consensus/Isolate | Core | Rank % | Binding affinity predictor |
| 38 | TDLKNYIHWF**H**KTQK | Consensus | IHWFHKTQK | 16.58 | 0 |
| 39 | DLKNYIHWF**H**KTQKK | Consensus | IHWFHKTQK | 4.69 | WB |
| 40 | LKNYIHWF**H**KTQKKP | Consensus | IHWFHKTQK | 1.96 | WB |
| 41 | KNYIHWF**H**KTQKKPW | Consensus | IHWFHKTQK | 1.94 | WB |
|  |  |  |  |  |  |
| 38 | TDLKNYIHWF**Y**KTQK | 7Pak | IHWFYKTQK | 19.35 | 0 |
| 39 | DLKNYIHWF**Y**KTQKK | 7Pak | IHWFYKTQK | 5.73 | NB |
| 40 | LKNYIHWF**Y**KTQKKP | 7Pak | IHWFYKTQK | 2.41 | WB |
| 41 | KNYIHWF**Y**KTQKKPW | 7Pak | IHWFYKTQK | 2.22 | WB |
|  |  |  |  |  |  |
| H48Y | 2Pak | 116:01:00 | Epitope 6 | 2WB --> 1WB, 1NB | Weakened binding affinity of Epitope to BoLA-DRB3 |
| Position | Peptide | Gag consensus/Isolate | Core | Rank % | Binding affinity predictor |
| 42 | NYIHWF**H**KTQKKPWT | Consensus | FHKTQKKPW | 3.51 | WB |
| 43 | YIHWF**H**KTQKKPWTF | Consensus | FHKTQKKPW | 4.80 | WB |
| 44 | IHWF**H**KTQKKPWTFT | Consensus | FHKTQKKPW | 5.70 | 0 |
| 45 | HWF**H**KTQKKPWTFTS | Consensus | FHKTQKKPW | 11.65 | 0 |
|  |  |  |  |  |  |
| 42 | NYIHWF**Y**KTQKKPWT | 2Pak | FYKTQKKPW | 3.72 | WB |
| 43 | YIHWF**Y**KTQKKPWTF | 2Pak | FYKTQKKPW | 5.67 | NB |
| 44 | IHWF**Y**KTQKKPWTFT | 2Pak | FYKTQKKPW | 7.26 | 0 |
| 45 | HWF**Y**KTQKKPWTFTS | 2Pak | FYKTQKKPW | 14.27 | 0 |
|  |  |  |  |  |  |
| H48Y | 3M | 018:01 | Epitope 6 | 2WB --> 1WB, 1NB | Weakened binding affinity of Epitope to BoLA-DRB3 |
| Position | Peptide | Gag consensus/Isolate | Core | Rank % | Binding affinity predictor |
| 42 | NYIHWF**H**KTQKKPWT | Consensus | IHWFHKTQK | 1.11 | WB |
| 43 | YIHWF**H**KTQKKPWTF | Consensus | FHKTQKKPW | 3.58 | WB |
| 44 | IHWF**H**KTQKKPWTFT | Consensus | FHKTQKKPW | 10.60 | 0 |
| 45 | HWF**H**KTQKKPWTFTS | Consensus | FHKTQKKPW | 21.94 | 0 |
|  |  |  |  |  |  |
| 42 | NYIHWF**Y**KTQKKPWT | 3M | IHWFYKTQK | 1.53 | WB |
| 43 | YIHWF**Y**KTQKKPWTF | 3M | FYKTQKKPW | 5.40 | NB |
| 44 | IHWF**Y**KTQKKPWTFT | 3M | FYKTQKKPW | 12.81 | 0 |
| 45 | HWF**Y**KTQKKPWTFTS | 3M | FYKTQKKPW | 24.92 | 0 |
|  |  |  |  |  |  |
| H48Y | 16M | 007:01 | Epitope 6 | 1WB --> 1NB | Lack of peptide interaction with BoLA-DRB3 |
| H48Y | 17K | 007:01 | Epitope 6 | 1WB --> 1NB | Lack of peptide interaction with BoLA-DRB3 |
| H48Y | 19K | 007:01 | Epitope 6 | 1WB --> 1NB | Lack of peptide interaction with BoLA-DRB3 |
| Position | Peptide | Gag consensus/Isolate | Core | Rank % | Binding affinity predictor |
| 42 | NYIHWF**H**KTQKKPWT | Consensus | IHWFHKTQK | 3.46 | WB |
| 43 | YIHWF**H**KTQKKPWTF | Consensus | FHKTQKKPW | 9.11 | 0 |
| 44 | IHWF**H**KTQKKPWTFT | Consensus | FHKTQKKPW | 18.90 | 0 |
| 45 | HWF**H**KTQKKPWTFTS | Consensus | FHKTQKKPW | 35.80 | 0 |
|  |  |  |  |  |  |
| 42 | NYIHWF**Y**KTQKKPWT | 16M | IHWFYKTQK | 5.63 | NB |
| 43 | YIHWF**Y**KTQKKPWTF | 16M | FYKTQKKPW | 15.34 | 0 |
| 44 | IHWF**Y**KTQKKPWTFT | 16M | FYKTQKKPW | 26.07 | 0 |
| 45 | HWF**Y**KTQKKPWTFTS | 16M | FYKTQKKPW | 39.31 | 0 |
|  |  |  |  |  |  |
| H48Y | 5P | 004:01 | Epitope 6 | 1SB, 1WB --> 1WB, 1NB | Weakened binding affinity of Epitope to BoLA-DRB3 |
| Position | Peptide | Gag consensus/Isolate | Core | Rank % | Binding affinity predictor |
| 42 | NYIHWF**H**KTQKKPWT | Consensus | IHWFHKTQK | 0.70 | SB |
| 43 | YIHWF**H**KTQKKPWTF | Consensus | IHWFHKTQK | 4.14 | WB |
| 44 | IHWF**H**KTQKKPWTFT | Consensus | FHKTQKKPW | 15.04 | 0 |
| 45 | HWF**H**KTQKKPWTFTS | Consensus | FHKTQKKPW | 28.79 | 0 |
|  |  |  |  |  |  |
| 42 | NYIHWF**Y**KTQKKPWT | 5P | IHWFYKTQK | 1.67 | WB |
| 43 | YIHWF**Y**KTQKKPWTF | 5P | IHWFYKTQK | 6.80 | NB |
| 44 | IHWF**Y**KTQKKPWTFT | 5P | FYKTQKKPW | 17.94 | 0 |
| 45 | HWF**Y**KTQKKPWTFTS | 5P | FYKTQKKPW | 30.40 | 0 |
|  |  |  |  |  |  |
| G61S, A63T | 23P | 024:03 | Epitope 11A | 3WB --> 3NB | Lack of peptides interactions with BoLA-DRB3 |
| Position | Peptide | Gag consensus/Isolate | Core | Rank % | Binding affinity predictor |
| 50 | TQKKPWTFTSG**G**P**A**S | Consensus | WTFTSGGPA | 2.17 | WB |
| 51 | QKKPWTFTSG**G**P**A**SC | Consensus | WTFTSGGPA | 3.47 | WB |
| 52 | KKPWTFTSG**G**P**A**SCP | Consensus | WTFTSGGPA | 2.18 | WB |
|  |  |  |  |  |  |
| 50 | TQKKPWTFTSG**S**P**T**S | 23P | WTFTSGSPT | 11.64 | NB |
| 51 | QKKPWTFTSG**S**P**T**SC | 23P | WTFTSGSPT | 15.80 | NB |
| 52 | KKPWTFTSG**S**P**T**SCP | 23P | WTFTSGSPT | 11.64 | NB |
|  |  |  |  |  |  |
|  |  |  |  |  |  |
| G61S, A63T | 28P | 009:04 | Epitope 11A | 2WB --> 2NB | Lack of peptides interactions with BoLA-DRB3 |
| Position | Peptide | Gag consensus/Isolate | Core | Rank % | Binding affinity predictor |
| 50 | TQKKPWTFTSG**G**P**A**S | Consensus | WTFTSGGPA | 3.24 | WB |
| 51 | QKKPWTFTSG**G**P**A**SC | Consensus | WTFTSGGPA | 5.27 | 0 |
| 52 | KKPWTFTSG**G**P**A**SCP | Consensus | WTFTSGGPA | 3.47 | WB |
|  |  |  |  |  |  |
| 50 | TQKKPWTFTSG**S**P**T**S | 28P | WTFTSGSPT | 13.44 | NB |
| 51 | QKKPWTFTSG**S**P**T**SC | 28P | WTFTSGSPT | 16.40 | 0 |
| 52 | KKPWTFTSG**S**P**T**SCP | 28P | WTFTSGSPT | 12.36 | NB |
|  |  |  |  |  |  |
| A63T | 3Kan | 107:01:00 | Epitope 11A | 2WB --> 2NB | Lack of peptides interactions with BoLA-DRB3 |
| Position | Peptide | Gag consensus/Isolate | Core | Rank % | Binding affinity predictor |
| 50 | TQKKPWTFTSGGP**A**S | Consensus | WTFTSGGPA | 4.20 | WB |
| 51 | QKKPWTFTSGGP**A**SC | Consensus | WTFTSGGPA | 5.05 | 0 |
| 52 | KKPWTFTSGGP**A**SCP | Consensus | WTFTSGGPA | 3.70 | WB |
|  |  |  |  |  |  |
| 50 | TQKKPWTFTSGGP**T**S | 3Kan | WTFTSGGPT | 5.71 | NB |
| 51 | QKKPWTFTSGGP**T**SC | 3Kan | WTFTSGGPT | 7.43 | 0 |
| 52 | KKPWTFTSGGP**T**SCP | 3Kan | WTFTSGGPT | 5.30 | NB |
|  |  |  |  |  |  |
|  |  |  |  |  |  |
| A63V | 1Pak | 080:01 | Epitope 11A | 3WB, 1NB --> 4WB | Strengthened binding affinity of Epitope to BoLA-DRB3 |
| Position | Peptide | Gag consensus/Isolate | Core | Rank % | Binding affinity predictor |
| 50 | TQKKPWTFTSGGP**A**S | Consensus | WTFTSGGPA | 2.52 | WB |
| 51 | QKKPWTFTSGGP**A**SC | Consensus | WTFTSGGPA | 3.84 | WB |
| 52 | KKPWTFTSGGP**A**SCP | Consensus | WTFTSGGPA | 2.71 | WB |
| 53 | KPWTFTSGGP**A**SCPP | Consensus | WTFTSGGPA | 9.10 | NB |
|  |  |  |  |  |  |
| 50 | TQKKPWTFTSGGP**V**S | 1Pak | WTFTSGGPV | 1.19 | WB |
| 51 | QKKPWTFTSGGP**V**SC | 1Pak | WTFTSGGPV | 1.82 | WB |
| 52 | KKPWTFTSGGP**V**SCP | 1Pak | WTFTSGGPV | 1.30 | WB |
| 53 | KPWTFTSGGP**V**SCPP | 1Pak | WTFTSGGPV | 4.60 | WB |
|  |  |  |  |  |  |
|  |  |  |  |  |  |
| A63V | 3Pak | 043:03 | Epitope 11A | 2NB --> 2WB | The presence of a new binding site for the BoLA-DRB3 |
| Position | Peptide | Gag consensus/Isolate | Core | Rank % | Binding affinity predictor |
| 50 | TQKKPWTFTSGGP**A**S | Consensus | WTFTSGGPA | 8.02 | NB |
| 51 | QKKPWTFTSGGP**A**SC | Consensus | WTFTSGGPA | 13.59 | 0 |
| 52 | KKPWTFTSGGP**A**SCP | Consensus | WTFTSGGPA | 10.02 | NB |
|  |  |  |  |  |  |
| 50 | TQKKPWTFTSGGP**V**S | 3Pak | WTFTSGGPV | 4.00 | WB |
| 51 | QKKPWTFTSGGP**V**SC | 3Pak | WTFTSGGPV | 6.21 | 0 |
| 52 | KKPWTFTSGGP**V**SCP | 3Pak | WTFTSGGPV | 4.65 | WB |
|  |  |  |  |  |  |
| K69R, A78T, DE87/88EG | 24Kan | 105:02:00 | Epitope 5 | 4WB --> 1WB, 3NB | Weakened binding affinity of Epitope to BoLA-DRB3 |
| K69R, A78T, DE87/88EG | 25Kan | 105:02:00 | Epitope 5 | 4WB --> 1WB, 3NB | Weakened binding affinity of Epitope to BoLA-DRB3 |
| Position | Peptide | Gag consensus/Isolate | Core | Rank % | Binding affinity predictor |
| 69 | **K**FGRVPLVL**A**TLNEV | Consensus | LVLATLNEV | 30.19 | 0 |
| 70 | FGRVPLVL**A**TLNEVL | Consensus | LVLATLNEV | 11.80 | 0 |
| 71 | GRVPLVL**A**TLNEVLS | Consensus | LVLATLNEV | 2.93 | WB |
| 72 | RVPLVL**A**TLNEVLSN | Consensus | VLATLNEVL | 1.67 | WB |
| 73 | VPLVL**A**TLNEVLSN**D** | Consensus | VLATLNEVL | 2.01 | WB |
| 74 | PLVL**A**TLNEVLSN**DE** | Consensus | VLATLNEVL | 4.81 | WB |
|  |  |  |  |  |  |
| 69 | **R**FGRVPLVL**T**TLNEV | 24Kan | LVLTTLNEV | 37.04 | 0 |
| 70 | FGRVPLVL**T**TLNEVL | 24Kan | LVLTTLNEV | 14.72 | 0 |
| 71 | GRVPLVL**T**TLNEVLS | 24Kan | LVLTTLNEV | 6.86 | NB |
| 72 | RVPLVL**T**TLNEVLSN | 24Kan | LVLTTLNEV | 4.23 | WB |
| 73 | VPLVL**T**TLNEVLSN**E** | 24Kan | VLTTLNEVL | 6.60 | NB |
| 74 | PLVL**T**TLNEVLSN**EG** | 24Kan | VLTTLNEVL | 13.86 | NB |
|  |  |  |  |  |  |
| K69R, DE87/88EG | 12Kan | 015:01 | Epitope 5 | 6WB --> 5WB, 1NB | Weakened binding affinity of Epitope to BoLA-DRB3 |
| K69R, DE87/88EG | 17Kan | 015:01 | Epitope 5 | 6WB --> 5WB, 1NB | Weakened binding affinity of Epitope to BoLA-DRB3 |
| K69R, DE87/88EG | 20Kan | 015:01 | Epitope 5 | 6WB --> 5WB, 1NB | Weakened binding affinity of Epitope to BoLA-DRB3 |
| K69R, DE87/88EG | 21Kan | 015:01 | Epitope 5 | 6WB --> 5WB, 1NB | Weakened binding affinity of Epitope to BoLA-DRB3 |
| K69R, DE87/88EG | 23Kan | 015:01 | Epitope 5 | 6WB --> 5WB, 1NB | Weakened binding affinity of Epitope to BoLA-DRB3 |
| Position | Peptide | Gag consensus/Isolate | Core | Rank % | Binding affinity predictor |
| 69 | **K**FGRVPLVLATLNEV | Consensus | LVLATLNEV | 4.22 | WB |
| 70 | FGRVPLVLATLNEVL | Consensus | LVLATLNEV | 2.14 | WB |
| 71 | GRVPLVLATLNEVLS | Consensus | LVLATLNEV | 1.40 | WB |
| 72 | RVPLVLATLNEVLSN | Consensus | LVLATLNEV | 1.12 | WB |
| 73 | VPLVLATLNEVLSN**D** | Consensus | VLATLNEVL | 1.57 | WB |
| 74 | PLVLATLNEVLSN**DE** | Consensus | VLATLNEVL | 4.41 | WB |
|  |  |  |  |  |  |
| 69 | **R**FGRVPLVLATLNEV | 12Kan | LVLATLNEV | 4.22 | WB |
| 70 | FGRVPLVLATLNEVL | 12Kan | LVLATLNEV | 2.14 | WB |
| 71 | GRVPLVLATLNEVLS | 12Kan | LVLATLNEV | 1.40 | WB |
| 72 | RVPLVLATLNEVLSN | 12Kan | LVLATLNEV | 1.12 | WB |
| 73 | VPLVLATLNEVLSN**E** | 12Kan | VLATLNEVL | 1.85 | WB |
| 74 | PLVLATLNEVLSN**EG** | 12Kan | VLATLNEVL | 5.29 | NB |
|  |  |  |  |  |  |
|  |  |  |  |  |  |
| K69R, DE87/88EG | 15Pak | 015:04 | Epitope 5 | 3SB, 3WB --> 2SB, 4WB | Weakened binding affinity of Epitope to BoLA-DRB3 |
| Position | Peptide | Gag consensus/Isolate | Core | Rank % | Binding affinity predictor |
| 69 | **K**FGRVPLVLATLNEV | Consensus | LVLATLNEV | 4.26 | WB |
| 70 | FGRVPLVLATLNEVL | Consensus | LVLATLNEV | 1.82 | WB |
| 71 | GRVPLVLATLNEVLS | Consensus | LVLATLNEV | 0.89 | SB |
| 72 | RVPLVLATLNEVLSN | Consensus | LVLATLNEV | 0.64 | SB |
| 73 | VPLVLATLNEVLSN**D** | Consensus | VLATLNEVL | 0.89 | SB |
| 74 | PLVLATLNEVLSN**DE** | Consensus | VLATLNEVL | 3.26 | WB |
|  |  |  |  |  |  |
| 69 | **R**FGRVPLVLATLNEV | 15Pak | LVLATLNEV | 4.28 | WB |
| 70 | FGRVPLVLATLNEVL | 15Pak | LVLATLNEV | 1.82 | WB |
| 71 | GRVPLVLATLNEVLS | 15Pak | LVLATLNEV | 0.89 | SB |
| 72 | RVPLVLATLNEVLSN | 15Pak | LVLATLNEV | 0.64 | SB |
| 73 | VPLVLATLNEVLSN**E** | 15Pak | VLATLNEVL | 1.08 | WB |
| 74 | PLVLATLNEVLSN**EG** | 15Pak | VLATLNEVL | 4.11 | WB |
|  |  |  |  |  |  |
|  |  |  |  |  |  |
| K69R, E82D i E88G | 4Kan | 15:01 | Epitope 5 | 6WB --> 4WB, 2NB | Weakened binding affinity of Epitope to BoLA-DRB3 |
| Position | Peptide | Gag consensus/Isolate | Core | Rank % | Binding affinity predictor |
| 69 | **K**FGRVPLVLATLN**E**V | Consensus | LVLATLNEV | 4.22 | WB |
| 70 | FGRVPLVLATLN**E**VL | Consensus | LVLATLNEV | 2.14 | WB |
| 71 | GRVPLVLATLN**E**VLS | Consensus | LVLATLNEV | 1.40 | WB |
| 72 | RVPLVLATLN**E**VLSN | Consensus | LVLATLNEV | 1.12 | WB |
| 73 | VPLVLATLN**E**VLSND | Consensus | VLATLNEVL | 1.57 | WB |
| 74 | PLVLATLN**E**VLSND**E** | Consensus | VLATLNEVL | 4.41 | WB |
|  |  |  |  |  |  |
| 69 | **R**FGRVPLVLATLN**D**V | 4Kan | LVLATLNDV | 5.54 | NB |
| 70 | FGRVPLVLATLN**D**VL | 4Kan | LVLATLNDV | 2.93 | WB |
| 71 | GRVPLVLATLN**D**VLS | 4Kan | LVLATLNDV | 2.02 | WB |
| 72 | RVPLVLATLN**D**VLSN | 4Kan | LVLATLNDV | 1.74 | WB |
| 73 | VPLVLATLN**D**VLSND | 4Kan | LVLATLNDV | 3.45 | WB |
| 74 | PLVLATLN**D**VLSND**G** | 4Kan | VLATLNDVL | 10.97 | NB |
|  |  |  |  |  |  |
| V76I | 18K | 032:01 | Epitope 5 | 2NB -->2WB | The presence of a new binding site for the BoLA-DRB3 |
| Position | Peptide | Gag consensus/Isolate | Core | Rank % | Binding affinity predictor |
| 69 | KFGRVPL**V**LATLNEV | Consensus | LVLATLNEV | 23.21 | 0 |
| 70 | FGRVPL**V**LATLNEVL | Consensus | LVLATLNEV | 13.95 | 0 |
| 71 | GRVPL**V**LATLNEVLS | Consensus | LVLATLNEV | 8.92 | 0 |
| 72 | RVPL**V**LATLNEVLSN | Consensus | LVLATLNEV | 6.16 | NB |
| 73 | VPL**V**LATLNEVLSND | Consensus | VLATLNEVL | 7.14 | NB |
| 74 | PL**V**LATLNEVLSNDE | Consensus | LATLNEVLS | 13.83 | 0 |
|  |  |  |  |  |  |
| 69 | KFGRVPL**I**LATLNEV | 18K | LILATLNEV | 27.12 | 0 |
| 70 | FGRVPL**I**LATLNEVL | 18K | LILATLNEV | 14.29 | 0 |
| 71 | GRVPL**I**LATLNEVLS | 18K | ILATLNEVL | 5.79 | 0 |
| 72 | RVPL**I**LATLNEVLSN | 18K | ILATLNEVL | 3.38 | WB |
| 73 | VPL**I**LATLNEVLSND | 18K | ILATLNEVL | 2.76 | WB |
| 74 | PL**I**LATLNEVLSNDE | 18K | ILATLNEVL | 6.42 | 0 |
|  |  |  |  |  |  |
| E82K | 1K | 013:01 | Epitope 5 | 4WB --> 3WB, 1NB | Weakened binding affinity of Epitope to BoLA-DRB3 |
| E82K | 2K | 013:01 | Epitope 5 | 4WB --> 3WB, 1NB | Weakened binding affinity of Epitope to BoLA-DRB3 |
| Position | Peptide | Gag consensus/Isolate | Core | Rank % | Binding affinity predictor |
| 69 | KFGRVPLVLATLN**E**V | Consensus | LVLATLNEV | 38.62 | 0 |
| 70 | FGRVPLVLATLN**E**VL | Consensus | LVLATLNEV | 13.80 | 0 |
| 71 | GRVPLVLATLN**E**VLS | Consensus | LVLATLNEV | 4.05 | WB |
| 72 | RVPLVLATLN**E**VLSN | Consensus | VLATLNEVL | 1.77 | WB |
| 73 | VPLVLATLN**E**VLSND | Consensus | VLATLNEVL | 1.51 | WB |
| 74 | PLVLATLN**E**VLSNDE | Consensus | VLATLNEVL | 4.17 | WB |
|  |  |  |  |  |  |
| 69 | KFGRVPLVLATLN**K**V | 1K | LVLATLNKV | 22.42 | 0 |
| 70 | FGRVPLVLATLN**K**VL | 1K | LVLATLNKV | 6.36 | 0 |
| 71 | GRVPLVLATLN**K**VLS | 1K | LVLATLNKV | 2.83 | WB |
| 72 | RVPLVLATLN**K**VLSN | 1K | LVLATLNKV | 1.40 | WB |
| 73 | VPLVLATLN**K**VLSND | 1K | LVLATLNKV | 3.20 | WB |
| 74 | PLVLATLN**K**VLSNDE | 1K | VLATLNKVL | 9.10 | NB |
|  |  |  |  |  |  |
|  |  |  |  |  |  |
| E82K | 1K | 130:01:00 | Epitope 5 | 5NB -->5WB | Presence of a new binding site for the BoLA-DRB3 |
| Position | Peptide | Gag consensus/Isolate | Core | Rank % | Binding affinity predictor |
| 69 | KFGRVPLVLATLN**E**V | Consensus | LVLATLNEV | 10.83 | 0 |
| 70 | FGRVPLVLATLN**E**VL | Consensus | LVLATLNEV | 9.48 | NB |
| 71 | GRVPLVLATLN**E**VLS | Consensus | LVLATLNEV | 7.04 | NB |
| 72 | RVPLVLATLN**E**VLSN | Consensus | LVLATLNEV | 6.29 | NB |
| 73 | VPLVLATLN**E**VLSND | Consensus | LVLATLNEV | 20.31 | NB |
| 74 | PLVLATLN**E**VLSNDE | Consensus | VLATLNEVL | 36.74 | NB |
|  |  |  |  |  |  |
| 69 | KFGRVPLVLATLN**K**V | 1K | LVLATLNKV | 5.45 | 0 |
| 70 | FGRVPLVLATLN**K**VL | 1K | LVLATLNKV | 4.06 | WB |
| 71 | GRVPLVLATLN**K**VLS | 1K | LVLATLNKV | 2.67 | WB |
| 72 | RVPLVLATLN**K**VLSN | 1K | LVLATLNKV | 1.73 | WB |
| 73 | VPLVLATLN**K**VLSND | 1K | LATLNKVLS | 2.59 | WB |
| 74 | PLVLATLN**K**VLSNDE | 1K | LATLNKVLS | 4.42 | WB |
|  |  |  |  |  |  |
| D104N | 20P | 014:01:01 | Epitope 11B | 1WB --> 1NB | Lack of peptide interaction with BoLA-DRB3 |
| Position | Peptide | Gag consensus/Isolate | Core | Rank % | Binding affinity predictor |
| 101 | PPY**D**PPAVLPIISEG | Consensus | PAVLPIISE | 10.95 | 0 |
| 102 | PY**D**PPAVLPIISEGN | Consensus | PAVLPIISE | 8.87 | 0 |
| 103 | Y**D**PPAVLPIISEGNR | Consensus | PAVLPIISE | 4.89 | WB |
| 104 | **D**PPAVLPIISEGNRN | Consensus | VLPIISEGN | 6.62 | 0 |
| 105 | PPAVLPIISEGNRNR | Consensus | VLPIISEGN | 10.25 | 0 |
|  |  |  |  |  |  |
| 101 | PPY**N**PPAVLPIISEG | 20P | PAVLPIISE | 11.06 | 0 |
| 102 | PY**N**PPAVLPIISEGN | 20P | PAVLPIISE | 10.60 | 0 |
| 103 | Y**N**PPAVLPIISEGNR | 20P | PAVLPIISE | 5.74 | NB |
| 104 | **N**PPAVLPIISEGNRN | 20P | VLPIISEGN | 7.43 | 0 |
| 105 | PPAVLPIISEGNRNR | 20P | VLPIISEGN | 10.25 | 0 |
|  |  |  |  |  |  |
|  |  |  |  |  |  |
| D104N, L109M, I112V | 18P | 009:01 | Epitope 11B | 1NB --> 1WB | The presence of a new binding site for the BoLA-DRB3 |
| Position | Peptide | Gag consensus/Isolate | Core | Rank % | Binding affinity predictor |
| 100 | PPPY**D**PPAV**L**PI**I**SE | Consensus | YDPPAVLPI | 11.49 | NB |
| 101 | PPY**D**PPAV**L**PI**I**SEG | Consensus | YDPPAVLPI | 28.30 | 0 |
| 102 | PY**D**PPAV**L**PI**I**SEGN | Consensus | VLPIISEGN | 41.03 | 0 |
| 103 | Y**D**PPAV**L**PI**I**SEGNR | Consensus | VLPIISEGN | 19.25 | 0 |
| 104 | **D**PPAV**L**PI**I**SEGNRN | Consensus | VLPIISEGN | 11.31 | 0 |
| 105 | PPAV**L**PI**I**SEGNRNR | Consensus | VLPIISEGN | 8.15 | 0 |
|  |  |  |  |  |  |
| 100 | PPPY**N**PPAV**M**PI**V**SE | 18P | YNPPAVMPI | 3.72 | WB |
| 101 | PPY**N**PPAV**M**PI**V**SEG | 18P | YNPPAVMPI | 11.23 | 0 |
| 102 | PY**N**PPAV**M**PI**V**SEGN | 18P | YNPPAVMPI | 33.90 | 0 |
| 103 | Y**N**PPAV**M**PI**V**SEGNR | 18P | VMPIVSEGN | 23.93 | 0 |
| 104 | **N**PPAV**M**PI**V**SEGNRN | 18P | VMPIVSEGN | 16.72 | 0 |
| 105 | PPAV**M**PI**V**SEGNRNR | 18P | VMPIVSEGN | 13.70 | 0 |
|  |  |  |  |  |  |
| V108I | 15Kan | 012:01 | Epitope 11B | 2NB -->2WB | The presence of a new binding site for the BoLA-DRB3 |
| Position | Peptide | Gag consensus/Isolate | Core | Rank % | Binding affinity predictor |
| 101 | PPYDPPA**V**LPIISEG | Consensus | YDPPAVLPI | 26.73 | 0 |
| 102 | PYDPPA**V**LPIISEGN | Consensus | YDPPAVLPI | 41.98 | 0 |
| 103 | YDPPA**V**LPIISEGNR | Consensus | VLPIISEGN | 20.52 | 0 |
| 104 | DPPA**V**LPIISEGNRN | Consensus | VLPIISEGN | 12.75 | NB |
| 105 | PPA**V**LPIISEGNRNR | Consensus | VLPIISEGN | 10.30 | NB |
|  |  |  |  |  |  |
| 101 | PPYDPPA**I**LPIISEG | 15Kan | YDPPAILPI | 28.29 | 0 |
| 102 | PYDPPA**I**LPIISEGN | 15Kan | ILPIISEGN | 25.28 | 0 |
| 103 | YDPPA**I**LPIISEGNR | 15Kan | ILPIISEGN | 7.09 | 0 |
| 104 | DPPA**I**LPIISEGNRN | 15Kan | ILPIISEGN | 4.44 | WB |
| 105 | PPA**I**LPIISEGNRNR | 15Kan | ILPIISEGN | 3.69 | WB |
|  |  |  |  |  |  |
|  |  |  |  |  |  |
| V108I | 2M | 028:01 | Epitope 11B | 2WB, 3NB --> 1SB, 4WB | Strengthened binding affinity of Epitope to BoLA-DRB3 |
| Position | Peptide | Gag consensus/Isolate | Core | Rank % | Binding affinity predictor |
| 101 | PPYDPPA**V**LPIISEG | Consensus | PAVLPIISE | 7.88 | 0 |
| 102 | PYDPPA**V**LPIISEGN | Consensus | PAVLPIISE | 4.79 | WB |
| 103 | YDPPA**V**LPIISEGNR | Consensus | PAVLPIISE | 3.22 | WB |
| 104 | DPPA**V**LPIISEGNRN | Consensus | PAVLPIISE | 5.74 | NB |
| 105 | PPA**V**LPIISEGNRNR | Consensus | VLPIISEGN | 12.82 | NB |
| 106 | PA**V**LPIISEGNRNRH | Consensus | VLPIISEGN | 27.55 | NB |
|  |  |  |  |  |  |
| 101 | PPPYDPPA**I**LPIISE | 2M | YDPPAILPI | 16.47 | 0 |
| 102 | PPYDPPA**I**LPIISEG | 2M | PAILPIISE | 4.59 | WB |
| 103 | PYDPPA**I**LPIISEGN | 2M | PAILPIISE | 2.00 | WB |
| 104 | YDPPA**I**LPIISEGNR | 2M | PAILPIISE | 0.96 | SB |
| 105 | DPPA**I**LPIISEGNRN | 2M | ILPIISEGN | 1.63 | WB |
| 106 | PPA**I**LPIISEGNRNR | 2M | ILPIISEGN | 4.07 | WB |
|  |  |  |  |  |  |
|  |  |  |  |  |  |
| V108I | 4M | 019:02 | Epitope 11B | 2NB --> 2WB | The presence of a new binding site for the BoLA-DRB3 |
| Position | Peptide | Gag consensus/Isolate | Core | Rank % | Binding affinity predictor |
| 101 | PPYDPPA**V**LPIISEG | Consensus | YDPPAVLPI | 24.79 | 0 |
| 102 | PYDPPA**V**LPIISEGN | Consensus | VLPIISEGN | 33.44 | 0 |
| 103 | YDPPA**V**LPIISEGNR | Consensus | VLPIISEGN | 16.50 | 0 |
| 104 | DPPA**V**LPIISEGNRN | Consensus | VLPIISEGN | 11.61 | NB |
| 105 | PPA**V**LPIISEGNRNR | Consensus | VLPIISEGN | 9.85 | NB |
|  |  |  |  |  |  |
|  |  |  |  |  |  |
| 101 | PPYDPPA**I**LPIISEG | 4M | YDPPAILPI | 22.50 | 0 |
| 102 | PYDPPA**I**LPIISEGN | 4M | ILPIISEGN | 23.48 | 0 |
| 103 | YDPPA**I**LPIISEGNR | 4M | ILPIISEGN | 7.24 | 0 |
| 104 | DPPA**I**LPIISEGNRN | 4M | ILPIISEGN | 4.21 | WB |
| 105 | PPA**I**LPIISEGNRNR | 4M | ILPIISEGN | 3.38 | WB |
|  |  |  |  |  |  |
|  |  |  |  |  |  |
| V108I | 4M | 005:04 | Epitope 11B | 3NB --> 3WB | The presence of a new binding site for the BoLA-DRB3 |
| Position | Peptide | Gag consensus/Isolate | Core | Rank % | Binding affinity predictor |
| 101 | PPYDPPA**V**LPIISEG | Consensus | YDPPAVLPI | 27.71 | 0 |
| 102 | PYDPPA**V**LPIISEGN | Consensus | VLPIISEGN | 37.48 | 0 |
| 103 | YDPPA**V**LPIISEGNR | Consensus | VLPIISEGN | 11.59 | NB |
| 104 | DPPA**V**LPIISEGNRN | Consensus | VLPIISEGN | 7.08 | NB |
| 105 | PPA**V**LPIISEGNRNR | Consensus | VLPIISEGN | 6.66 | NB |
|  |  |  |  |  |  |
| 101 | PPYDPPA**I**LPIISEG | 4M | YDPPAILPI | 28.31 | 0 |
| 102 | PYDPPA**I**LPIISEGN | 4M | ILPIISEGN | 18.85 | 0 |
| 103 | YDPPA**I**LPIISEGNR | 4M | ILPIISEGN | 3.75 | WB |
| 104 | DPPA**I**LPIISEGNRN | 4M | ILPIISEGN | 1.96 | WB |
| 105 | PPA**I**LPIISEGNRNR | 4M | ILPIISEGN | 1.50 | WB |
|  |  |  |  |  |  |
|  |  |  |  |  |  |
| V108I | 5M | 011:02 | Epitope 11B | 4WB --> 1SB, 4WB | Strengthened binding affinity of Epitope to BoLA-DRB3 |
| Position | Peptide | Gag consensus/Isolate | Core | Rank % | Binding affinity predictor |
| 101 | PPYDPPA**V**LPIISEG | Consensus | PAVLPIISE | 3.46 | WB |
| 102 | PYDPPA**V**LPIISEGN | Consensus | PAVLPIISE | 2.12 | WB |
| 103 | YDPPA**V**LPIISEGNR | Consensus | PAVLPIISE | 1.59 | WB |
| 104 | DPPA**V**LPIISEGNRN | Consensus | PAVLPIISE | 2.59 | WB |
| 105 | PPA**V**LPIISEGNRNR | Consensus | VLPIISEGN | 5.47 | 0 |
| 106 | PA**V**LPIISEGNRNRH | Consensus | VLPIISEGN | 18.73 | 0 |
|  |  |  |  |  |  |
| 101 | PPPYDPPA**I**LPIISE | 5M | PAILPIISE | 6.45 | 0 |
| 102 | PPYDPPA**I**LPIISEG | 5M | PAILPIISE | 2.43 | WB |
| 103 | PYDPPA**I**LPIISEGN | 5M | PAILPIISE | 1.26 | WB |
| 104 | YDPPA**I**LPIISEGNR | 5M | PAILPIISE | 0.92 | SB |
| 105 | DPPA**I**LPIISEGNRN | 5M | PAILPIISE | 1.33 | WB |
| 106 | PPA**I**LPIISEGNRNR | 5M | ILPIISEGN | 2.44 | WB |
|  |  |  |  |  |  |
|  |  |  |  |  |  |
| V108I | 4K | 017:01 | Epitope 11B | 3NB -->3WB | The presence of a new binding site for the BoLA-DRB3 |
| V108I | 5K | 017:01 | Epitope 11B | 3NB -->3WB | The presence of a new binding site for the BoLA-DRB3 |
| Position | Peptide | Gag consensus/Isolate | Core | Rank % | Binding affinity predictor |
| 101 | PPYDPPA**V**LPIISEG | Consensus | YDPPAVLPI | 47.95 | 0 |
| 102 | PYDPPA**V**LPIISEGN | Consensus | VLPIISEGN | 30.71 | 0 |
| 103 | YDPPA**V**LPIISEGNR | Consensus | VLPIISEGN | 10.61 | 0 |
| 104 | DPPA**V**LPIISEGNRN | Consensus | VLPIISEGN | 6.74 | NB |
| 105 | PPA**V**LPIISEGNRNR | Consensus | VLPIISEGN | 6.04 | NB |
| 106 | PA**V**LPIISEGNRNRH | Consensus | VLPIISEGN | 15.14 | NB |
|  |  |  |  |  |  |
| 101 | PPPYDPPA**I**LPIISE | 4K | YDPPAILPI | 30.66 | 0 |
| 102 | PPYDPPA**I**LPIISEG | 4K | YDPPAILPI | 51.49 | 0 |
| 103 | PYDPPA**I**LPIISEGN | 4K | ILPIISEGN | 14.21 | 0 |
| 104 | YDPPA**I**LPIISEGNR | 4K | ILPIISEGN | 3.48 | WB |
| 105 | DPPA**I**LPIISEGNRN | 4K | ILPIISEGN | 1.89 | WB |
| 106 | PPA**I**LPIISEGNRNR | 4K | ILPIISEGN | 1.57 | WB |
|  |  |  |  |  |  |
|  |  |  |  |  |  |
| V108I | 4K | 009:01 | Epitope 11B | 2NB -->2WB | The presence of a new binding site for the BoLA-DRB3 |
| V108I | 5K | 009:01 | Epitope 11B | 2NB -->2WB | The presence of a new binding site for the BoLA-DRB3 |
| Position | Peptide | Gag consensus/Isolate | Core | Rank % | Binding affinity predictor |
| 101 | PPYDPPA**V**LPIISEG | Consensus | YDPPAVLPI | 28.30 | 0 |
| 102 | PYDPPA**V**LPIISEGN | Consensus | VLPIISEGN | 41.03 | 0 |
| 103 | YDPPA**V**LPIISEGNR | Consensus | VLPIISEGN | 19.25 | 0 |
| 104 | DPPA**V**LPIISEGNRN | Consensus | VLPIISEGN | 11.31 | 0 |
| 105 | PPA**V**LPIISEGNRNR | Consensus | VLPIISEGN | 8.15 | NB |
| 106 | PA**V**LPIISEGNRNRH | Consensus | VLPIISEGN | 16.89 | NB |
|  |  |  |  |  |  |
| 101 | PPPYDPPA**I**LPIISE | 4K | YDPPAILPI | 13.66 | 0 |
| 102 | PPYDPPA**I**LPIISEG | 4K | YDPPAILPI | 32.66 | 0 |
| 103 | PYDPPA**I**LPIISEGN | 4K | ILPIISEGN | 29.26 | 0 |
| 104 | YDPPA**I**LPIISEGNR | 4K | ILPIISEGN | 7.76 | 0 |
| 105 | DPPA**I**LPIISEGNRN | 4K | ILPIISEGN | 3.32 | WB |
| 106 | PPA**I**LPIISEGNRNR | 4K | ILPIISEGN | 2.34 | WB |
|  |  |  |  |  |  |
|  |  |  |  |  |  |
| V108I | 23K | 045:01 | Epitope 11B | 3NB --> 3WB | The presence of a new binding site for the BoLA-DRB3 |
| Position | Peptide | Gag consensus/Isolate | Core | Rank % | Binding affinity predictor |
| 101 | PPYDPPA**V**LPIISEG | Consensus | YDPPAVLPI | 21.39 | 0 |
| 102 | PYDPPA**V**LPIISEGN | Consensus | PAVLPIISE | 28.74 | 0 |
| 103 | YDPPA**V**LPIISEGNR | Consensus | VLPIISEGN | 13.27 | NB |
| 104 | DPPA**V**LPIISEGNRN | Consensus | VLPIISEGN | 10.16 | NB |
| 105 | PPA**V**LPIISEGNRNR | Consensus | VLPIISEGN | 9.58 | NB |
|  |  |  |  |  |  |
|  |  |  |  |  |  |
| 101 | PPYDPPA**I**LPIISEG | 23K | YDPPAILPI | 22.45 | 0 |
| 102 | PYDPPA**I**LPIISEGN | 23K | ILPIISEGN | 15.25 | 0 |
| 103 | YDPPA**I**LPIISEGNR | 23K | ILPIISEGN | 4.48 | WB |
| 104 | DPPA**I**LPIISEGNRN | 23K | ILPIISEGN | 2.74 | WB |
| 105 | PPA**I**LPIISEGNRNR | 23K | ILPIISEGN | 2.26 | WB |
|  |  |  |  |  |  |
|  |  |  |  |  |  |
| V108I | 23K | 134:01:00 | Epitope 11B | 2NB --> 2WB | The presence of a new binding site for the BoLA-DRB3 |
| Position | Peptide | Gag consensus/Isolate | Core | Rank % | Binding affinity predictor |
| 101 | PPYDPPA**V**LPIISEG | Consensus | PAVLPIISE | 21.13 | 0 |
| 102 | PYDPPA**V**LPIISEGN | Consensus | PAVLPIISE | 15.46 | 0 |
| 103 | YDPPA**V**LPIISEGNR | Consensus | PAVLPIISE | 9.95 | NB |
| 104 | DPPA**V**LPIISEGNRN | Consensus | VLPIISEGN | 13.24 | NB |
| 105 | PPA**V**LPIISEGNRNR | Consensus | VLPIISEGN | 19.52 | 0 |
|  |  |  |  |  |  |
| 101 | PPYDPPA**I**LPIISEG | 23K | PAILPIISE | 18.08 | 0 |
| 102 | PYDPPA**I**LPIISEGN | 23K | PAILPIISE | 9.63 | 0 |
| 103 | YDPPA**I**LPIISEGNR | 23K | ILPIISEGN | 4.29 | WB |
| 104 | DPPA**I**LPIISEGNRN | 23K | ILPIISEGN | 4.55 | WB |
| 105 | PPA**I**LPIISEGNRNR | 23K | ILPIISEGN | 6.48 | 0 |
|  |  |  |  |  |  |
|  |  |  |  |  |  |
| V108I | 24K | 007:01 | Epitope 11B | 2WB, 2NB --> 4WB | Strengthened binding affinity of Epitope to BoLA-DRB3 |
| Position | Peptide | Gag consensus/Isolate | Core | Rank % | Binding affinity predictor |
| 101 | PPYDPPA**V**LPIISEG | Consensus | PAVLPIISE | 13.49 | 0 |
| 102 | PYDPPA**V**LPIISEGN | Consensus | PAVLPIISE | 9.37 | NB |
| 103 | YDPPA**V**LPIISEGNR | Consensus | PAVLPIISE | 4.69 | WB |
| 104 | DPPA**V**LPIISEGNRN | Consensus | VLPIISEGN | 4.82 | WB |
| 105 | PPA**V**LPIISEGNRNR | Consensus | VLPIISEGN | 9.72 | NB |
|  |  |  |  |  |  |
| 101 | PPYDPPA**I**LPIISEG | 24K | PAILPIISE | 10.67 | 0 |
| 102 | PYDPPA**I**LPIISEGN | 24K | PAILPIISE | 4.34 | WB |
| 103 | YDPPA**I**LPIISEGNR | 24K | ILPIISEGN | 2.14 | WB |
| 104 | DPPA**I**LPIISEGNRN | 24K | ILPIISEGN | 2.07 | WB |
| 105 | PPA**I**LPIISEGNRNR | 24K | ILPIISEGN | 3.53 | WB |
|  |  |  |  |  |  |
|  |  |  |  |  |  |
| V108I | 3Ż | 011:01 | Epitope 11B | 4WB, 1NB --> 5WB | Strengthened binding affinity of Epitope to BoLA-DRB3 |
| Position | Peptide | Gag consensus/Isolate | Core | Rank % | Binding affinity predictor |
| 101 | PPYDPPA**V**LPIISEG | Consensus | PAVLPIISE | 3.08 | WB |
| 102 | PYDPPA**V**LPIISEGN | Consensus | PAVLPIISE | 2.32 | WB |
| 103 | YDPPA**V**LPIISEGNR | Consensus | PAVLPIISE | 1.96 | WB |
| 104 | DPPA**V**LPIISEGNRN | Consensus | PAVLPIISE | 2.63 | WB |
| 105 | PPA**V**LPIISEGNRNR | Consensus | VLPIISEGN | 5.80 | NB |
|  |  |  |  |  |  |
| 101 | PPYDPPA**I**LPIISEG | 3Z | PAILPIISE | 2.41 | WB |
| 102 | PYDPPA**I**LPIISEGN | 3Z | PAILPIISE | 1.57 | WB |
| 103 | YDPPA**I**LPIISEGNR | 3Z | PAILPIISE | 1.25 | WB |
| 104 | DPPA**I**LPIISEGNRN | 3Z | PAILPIISE | 1.78 | WB |
| 105 | PPA**I**LPIISEGNRNR | 3Z | ILPIISEGN | 3.21 | WB |
|  |  |  |  |  |  |
| L109M, I112V | 24P | 014:01:01 | Epitope 11A | 1WB --> 1NB | Lack of peptide interaction with BoLA-DRB3 |
| Position | Peptide | Gag consensus/Isolate | Core | Rank % | Binding affinity predictor |
| 101 | PPYDPPAV**L**PI**I**SEG | Consensus | PAVLPIISE | 10.95 | 0 |
| 102 | PYDPPAV**L**PI**I**SEGN | Consensus | PAVLPIISE | 8.87 | 0 |
| 103 | YDPPAV**L**PI**I**SEGNR | Consensus | PAVLPIISE | 4.89 | WB |
| 104 | DPPAV**L**PI**I**SEGNRN | Consensus | VLPIISEGN | 6.62 | 0 |
| 105 | PPAV**L**PI**I**SEGNRNR | Consensus | VLPIISEGN | 10.25 | 0 |
|  |  |  |  |  |  |
| 101 | PPYDPPAV**M**PI**V**SEG | 24P | PAVMPIVSE | 22.38 | 0 |
| 102 | PYDPPAV**M**PI**V**SEGN | 24P | PAVMPIVSE | 17.54 | 0 |
| 103 | YDPPAV**M**PI**V**SEGNR | 24P | PAVMPIVSE | 9.95 | NB |
| 104 | DPPAV**M**PI**V**SEGNRN | 24P | VMPIVSEGN | 10.73 | 0 |
| 105 | PPAV**M**PI**V**SEGNRNR | 24P | VMPIVSEGN | 17.28 | 0 |
|  |  |  |  |  |  |
| V144I | 12Kan | 015:01 | Epitope 13A | 2WB --> 2NB | Lack of peptides interactions with BoLA-DRB3 |
| V144I | 17Kan | 015:01 | Epitope 13A | 2WB --> 2NB | Lack of peptides interactions with BoLA-DRB3 |
| V144I | 20Kan | 015:01 | Epitope 13A | 2WB --> 2NB | Lack of peptides interactions with BoLA-DRB3 |
| Position | Peptide | Gag consensus/Isolate | Core | Rank % | Binding affinity predictor |
| 141 | GSQ**V**WIQTLRLAILQ | Consensus | WIQTLRLAI | 4.58 | WB |
| 142 | SQ**V**WIQTLRLAILQA | Consensus | WIQTLRLAI | 4.59 | WB |
| 143 | Q**V**WIQTLRLAILQAD | Consensus | WIQTLRLAI | 8.01 | 0 |
|  |  |  |  |  |  |
| 141 | GSQ**I**WIQTLRLAILQ | 12Kan | WIQTLRLAI | 5.01 | NB |
| 142 | SQ**I**WIQTLRLAILQA | 12Kan | WIQTLRLAI | 5.04 | NB |
| 143 | Q**I**WIQTLRLAILQAD | 12Kan | WIQTLRLAI | 8.81 | 0 |
|  |  |  |  |  |  |
| Mutation: A189T | Isolate:24K | BoLA-DRB3*007:01 | Epitope: 9 | Effect: 1WB --> 1NB | →Lack of peptide interaction with BoLA-DRB3 |
| Position | Peptide | Gag consensus/Isolate | Core | Rank % | Binding affinity predictor |
| 182 | SLTAAIA**A**AEAANTL | Consensus | IAAAEAANT | 6.62 | 0 |
| 183 | LTAAIA**A**AEAANTLQ | Consensus | IAAAEAANT | 5.45 | 0 |
| 184 | TAAIA**A**AEAANTLQG | Consensus | IAAAEAANT | 4.73 | WB |
|  |  |  |  |  |  |
| 182 | SLTAAIA**T**AEAANTL | 24K | IATAEAANT | 16.44 | 0 |
| 183 | LTAAIA**T**AEAANTLQ | 24K | IATAEAANT | 13.36 | 0 |
| 184 | TAAIA**T**AEAANTLQG | 24K | IATAEAANT | 12.26 | NB |
|  |  |  |  |  |  |
|  |  |  |  |  |  |
| A193T | 30P | 006:01 | Epitope 9 | 2WB --> 2NB | Lack of peptides interactions with BoLA-DRB3 |
| Position | Peptide | Gag consensus/Isolate | Core | Rank % | Binding affinity predictor |
| 182 | SLTAAIAAAEA**A**NTL | Consensus | IAAAEAANT | 8.06 | 0 |
| 183 | LTAAIAAAEA**A**NTLQ | Consensus | IAAAEAANT | 4.45 | WB |
| 184 | TAAIAAAEA**A**NTLQG | Consensus | IAAAEAANT | 4.00 | WB |
|  |  |  |  |  |  |
| 182 | SLTAAIAAAEA**T**NTL | 30P | IAAAEATNT | 15.80 | 0 |
| 183 | LTAAIAAAEA**T**NTLQ | 30P | IAAAEATNT | 11.21 | NB |
| 184 | TAAIAAAEA**T**NTLQG | 30P | IAAAEATNT | 10.65 | NB |
|  |  |  |  |  |  |
| A250T, V254I | 1Pak | 080:01 | Epitope 13B | 2WB --> 2NB | Lack of peptides interactions with BoLA-DRB3 |
| Position | Peptide | Gag consensus/Isolate | Core | Rank % | Binding affinity predictor |
| 237 | PSVQPWSTIVQGP**A**E | Consensus | WSTIVQGPA | 5.11 | 0 |
| 238 | SVQPWSTIVQGP**A**ES | Consensus | WSTIVQGPA | 4.13 | WB |
| 239 | VQPWSTIVQGP**A**ESY | Consensus | WSTIVQGPA | 4.93 | WB |
| 240 | QPWSTIVQGP**A**ESY**V** | Consensus | WSTIVQGPA | 10.39 | 0 |
| 241 | PWSTIVQGP**A**ESY**V**E | Consensus | IVQGPAESY | 22.55 | 0 |
|  |  |  |  |  |  |
| 237 | PSVQPWSTIVQGP**T**E | 1Pak | WSTIVQGPT | 6.02 | 0 |
| 238 | SVQPWSTIVQGP**T**ES | 1Pak | WSTIVQGPT | 5.34 | NB |
| 239 | VQPWSTIVQGP**T**ESY | 1Pak | WSTIVQGPT | 6.01 | NB |
| 240 | QPWSTIVQGP**T**ESY**I** | 1Pak | WSTIVQGPT | 8.81 | 0 |
| 241 | PWSTIVQGP**T**ESY**I**E | 1Pak | IVQGPTESY | 19.72 | 0 |
|  |  |  |  |  |  |
|  |  |  |  |  |  |
| A250T, V254I | 1Pak | 139:01:00 | Epitope 3 | 2WB --> 1WB, 1NB | Weakened binding affinity of Epitope to BoLA-DRB3 |
| Position | Peptide | Gag consensus/Isolate | Core | Rank % | Binding affinity predictor |
| 247 | QGP**A**ESY**V**EFVNRLQ | Consensus | YVEFVNRLQ | 10.97 | 0 |
| 248 | GP**A**ESY**V**EFVNRLQI | Consensus | YVEFVNRLQ | 4.36 | WB |
| 249 | P**A**ESY**V**EFVNRLQIS | Consensus | YVEFVNRLQ | 2.77 | WB |
| 250 | **A**ESY**V**EFVNRLQISL | Consensus | YVEFVNRLQ | 5.28 | 0 |
| 251 | ESY**V**EFVNRLQISLA | Consensus | YVEFVNRLQ | 11.68 | 0 |
| 252 | SY**V**EFVNRLQISLAD | Consensus | VNRLQISLA | 20.68 | 0 |
| 253 | Y**V**EFVNRLQISLADN | Consensus | VNRLQISLA | 22.23 | 0 |
|  |  |  |  |  |  |
| 247 | QGP**T**ESY**I**EFVNRLQ | 1Pak | YIEFVNRLQ | 14.78 | 0 |
| 248 | GP**T**ESY**I**EFVNRLQI | 1Pak | YIEFVNRLQ | 6.31 | NB |
| 249 | P**T**ESY**I**EFVNRLQIS | 1Pak | YIEFVNRLQ | 3.85 | WB |
| 250 | **T**ESY**I**EFVNRLQISL | 1Pak | YIEFVNRLQ | 7.31 | 0 |
| 251 | ESY**I**EFVNRLQISLA | 1Pak | YIEFVNRLQ | 13.92 | 0 |
| 252 | SY**I**EFVNRLQISLAD | 1Pak | VNRLQISLA | 22.95 | 0 |
| 253 | Y**I**EFVNRLQISLADN | 1Pak | VNRLQISLA | 23.63 | 0 |
|  |  |  |  |  |  |
|  |  |  |  |  |  |
| A250T, V254I | 2Pak | 116:01:00 | Epitope 3 | 3WB, 2NB --> 3SB, 2WB | Strengthened binding affinity of Epitope to BoLA-DRB3 |
| Position | Peptide | Gag consensus/Isolate | Core | Rank % | Binding affinity predictor |
| 247 | QGP**A**ESY**V**EFVNRLQ | Consensus | YVEFVNRLQ | 69.59 | 0 |
| 248 | GP**A**ESY**V**EFVNRLQI | Consensus | VEFVNRLQI | 8.17 | NB |
| 249 | P**A**ESY**V**EFVNRLQIS | Consensus | VEFVNRLQI | 1.76 | WB |
| 250 | **A**ESY**V**EFVNRLQISL | Consensus | VEFVNRLQI | 2.67 | WB |
| 251 | ESY**V**EFVNRLQISLA | Consensus | VEFVNRLQI | 2.47 | WB |
| 252 | SY**V**EFVNRLQISLAD | Consensus | FVNRLQISL | 6.96 | NB |
| 253 | Y**V**EFVNRLQISLADN | Consensus | FVNRLQISL | 11.17 | 0 |
|  |  |  |  |  |  |
| 247 | QGP**T**ESY**I**EFVNRLQ | 2Pak | SYIEFVNRL | 69.87 | 0 |
| 248 | GP**T**ESY**I**EFVNRLQI | 2Pak | IEFVNRLQI | 3.51 | WB |
| 249 | P**T**ESY**I**EFVNRLQIS | 2Pak | IEFVNRLQI | 0.43 | SB |
| 250 | **T**ESY**I**EFVNRLQISL | 2Pak | IEFVNRLQI | 0.65 | SB |
| 251 | ESY**I**EFVNRLQISLA | 2Pak | IEFVNRLQI | 0.61 | SB |
| 252 | SY**I**EFVNRLQISLAD | 2Pak | IEFVNRLQI | 2.98 | WB |
| 253 | Y**I**EFVNRLQISLADN | 2Pak | FVNRLQISL | 9.80 | 0 |
|  |  |  |  |  |  |
|  |  |  |  |  |  |
| A250T, V254I | 3Pak | 057:02 | Epitope 13B | 1NB --> 1WB | New binding site for BoLA-DRB3 |
| A250T, V254I | 4Pak | 057:02 | Epitope 13B | 1NB --> 1WB | New binding site for BoLA-DRB3 |
| A250T, V254I | 5Pak | 057:02 | Epitope 13B | 1NB --> 1WB | New binding site for BoLA-DRB3 |
| A250T, V254I | 6Pak | 057:02 | Epitope 13B | 1NB --> 1WB | New binding site for BoLA-DRB3 |
| Position | Peptide | Gag consensus/Isolate | Core | Rank % | Binding affinity predictor |
| 237 | PSVQPWSTIVQGP**A**E | Consensus | VQPWSTIVQ | 18.59 | 0 |
| 238 | SVQPWSTIVQGP**A**ES | Consensus | WSTIVQGPA | 26.40 | 0 |
| 239 | VQPWSTIVQGP**A**ESY | Consensus | WSTIVQGPA | 26.14 | 0 |
| 241 | PWSTIVQGP**A**ESY**V**E | Consensus | IVQGPAESY | 11.53 | NB |
|  |  |  |  |  |  |
| 237 | PSVQPWSTIVQGP**T**E | 3Pak | VQPWSTIVQ | 19.77 | 0 |
| 238 | SVQPWSTIVQGP**T**ES | 3Pak | WSTIVQGPT | 25.61 | 0 |
| 239 | VQPWSTIVQGP**T**ESY | 3Pak | WSTIVQGPT | 16.06 | 0 |
| 241 | PWSTIVQGP**T**ESY**I**E | 3Pak | IVQGPTESY | 3.45 | WB |
|  |  |  |  |  |  |
|  |  |  |  |  |  |
| A250T, V254I | 7Pak | 018:01 | Epitope 3 | 3WB, 1NB -->1SB, 3WB | Strengthened binding affinity of Epitope to BoLA-DRB3 |
| Position | Peptide | Gag consensus/Isolate | Core | Rank % | Binding affinity predictor |
| 247 | QGP**A**ESY**V**EFVNRLQ | Consensus | YVEFVNRLQ | 72.42 | 0 |
| 248 | GP**A**ESY**V**EFVNRLQI | Consensus | VEFVNRLQI | 10.28 | 0 |
| 249 | P**A**ESY**V**EFVNRLQIS | Consensus | VEFVNRLQI | 2.87 | WB |
| 250 | **A**ESY**V**EFVNRLQISL | Consensus | VEFVNRLQI | 3.66 | WB |
| 251 | ESY**V**EFVNRLQISLA | Consensus | VEFVNRLQI | 3.50 | WB |
| 252 | SY**V**EFVNRLQISLAD | Consensus | VEFVNRLQI | 8.84 | NB |
| 253 | Y**V**EFVNRLQISLADN | Consensus | FVNRLQISL | 17.80 | 0 |
|  |  |  |  |  |  |
| 247 | QGP**T**ESY**I**EFVNRLQ | 7Pak | YIEFVNRLQ | 77.57 | 0 |
| 248 | GP**T**ESY**I**EFVNRLQI | 7Pak | IEFVNRLQI | 5.83 | 0 |
| 249 | P**T**ESY**I**EFVNRLQIS | 7Pak | IEFVNRLQI | 0.97 | SB |
| 250 | **T**ESY**I**EFVNRLQISL | 7Pak | IEFVNRLQI | 1.37 | WB |
| 251 | ESY**I**EFVNRLQISLA | 7Pak | IEFVNRLQI | 1.34 | WB |
| 252 | SY**I**EFVNRLQISLAD | 7Pak | IEFVNRLQI | 4.15 | WB |
| 253 | Y**I**EFVNRLQISLADN | 7Pak | IEFVNRLQI | 14.11 | 0 |
|  |  |  |  |  |  |
|  |  |  |  |  |  |
| A250T, V254I | 8Pak | 024:33 | Epitope 13B | 2NB --> 2WB | The presence of a new binding site for the BoLA-DRB3 |
| Position | Peptide | Gag consensus/Isolate | Core | Rank % | Binding affinity predictor |
| 237 | PSVQPWSTIVQGP**A**E | Consensus | VQPWSTIVQ | 19.38 | 0 |
| 238 | SVQPWSTIVQGP**A**ES | Consensus | WSTIVQGPA | 24.53 | 0 |
| 239 | VQPWSTIVQGP**A**ESY | Consensus | WSTIVQGPA | 18.81 | 0 |
| 240 | QPWSTIVQGP**A**ESYV | Consensus | IVQGPAESY | 14.86 | 0 |
| 241 | PWSTIVQGP**A**ESYVE | Consensus | IVQGPAESY | 7.82 | NB |
| 242 | WSTIVQGP**A**ESY**V**EF | Consensus | IVQGPAESY | 8.16 | NB |
|  |  |  |  |  |  |
| 237 | PSVQPWSTIVQGP**T**E | 8Pak | VQPWSTIVQ | 22.84 | 0 |
| 238 | SVQPWSTIVQGP**T**ES | 8Pak | WSTIVQGPT | 33.32 | 0 |
| 239 | VQPWSTIVQGP**T**ESY | 8Pak | IVQGPTESY | 19.52 | 0 |
| 240 | QPWSTIVQGP**T**ESY**I** | 8Pak | IVQGPTESY | 6.83 | 0 |
| 241 | PWSTIVQGP**T**ESY**I**E | 8Pak | IVQGPTESY | 3.63 | WB |
| 242 | WSTIVQGP**T**ESY**I**EF | 8Pak | IVQGPTESY | 4.75 | WB |
|  |  |  |  |  |  |
|  |  |  |  |  |  |
| A250T, V254I | 8Pak | 024:33 | Epitope 3 | 1NB --> 1WB | The presence of a new binding site for the BoLA-DRB3 |
| Position | Peptide | Consensus/Isolate | Core | Rank % | Binding affinity predictor |
| 247 | QGP**A**ESY**V**EFVNRLQ | Consensus | YVEFVNRLQ | 33.78 | 0 |
| 248 | GP**A**ESY**V**EFVNRLQI | Consensus | YVEFVNRLQ | 13.81 | 0 |
| 249 | P**A**ESY**V**EFVNRLQIS | Consensus | YVEFVNRLQ | 5.81 | NB |
| 250 | AESY**V**EFVNRLQISL | Consensus | YVEFVNRLQ | 8.20 | 0 |
| 251 | ESY**V**EFVNRLQISLA | Consensus | VEFVNRLQI | 12.25 | 0 |
| 252 | SY**V**EFVNRLQISLAD | Consensus | VEFVNRLQI | 32.67 | 0 |
| 253 | Y**V**EFVNRLQISLADN | Consensus | VNRLQISLA | 44.48 | 0 |
|  |  |  |  |  |  |
| 247 | QGP**T**ESY**I**EFVNRLQ | 8Pak | YIEFVNRLQ | 35.51 | 0 |
| 248 | GP**T**ESY**I**EFVNRLQI | 8Pak | YIEFVNRLQ | 13.16 | 0 |
| 249 | P**T**ESY**I**EFVNRLQIS | 8Pak | IEFVNRLQI | 4.57 | WB |
| 250 | **T**ESY**I**EFVNRLQISL | 8Pak | IEFVNRLQI | 7.77 | 0 |
| 251 | ESY**I**EFVNRLQISLA | 8Pak | IEFVNRLQI | 10.21 | 0 |
| 252 | SY**I**EFVNRLQISLAD | 8Pak | IEFVNRLQI | 20.98 | 0 |
| 253 | Y**I**EFVNRLQISLADN | 8Pak | VNRLQISLA | 42.55 | 0 |
|  |  |  |  |  |  |
| I318V, I323V | 22Kan | 027:18 | Epitope 1A | 4WB --> 2WB, 2NB | Weakened binding affinity of Epitope to BoLA-DRB3 |
| Position | Peptide | Gag consensus/Isolate | Core | Rank % | Binding affinity predictor |
| 317 | K**I**KQPA**I**LVHTPGPK | Consensus | ILVHTPGPK | 10.78 | 0 |
| 318 | **I**KQPA**I**LVHTPGPKM | Consensus | ILVHTPGPK | 4.03 | WB |
| 319 | KQPA**I**LVHTPGPKMP | Consensus | ILVHTPGPK | 2.27 | WB |
| 320 | QPA**I**LVHTPGPKMPG | Consensus | ILVHTPGPK | 1.78 | WB |
| 321 | PA**I**LVHTPGPKMPGP | Consensus | ILVHTPGPK | 2.80 | WB |
| 322 | AILVHTPGPKMPGPR | Consensus | LVHTPGPKM | 16.33 | 0 |
|  |  |  |  |  |  |
| 317 | K**V**KQPA**V**LVHTPGPK | 22Kan | VLVHTPGPK | 18.76 | 0 |
| 318 | **V**KQPA**V**LVHTPGPKM | 22Kan | VLVHTPGPK | 9.10 | NB |
| 319 | KQPA**V**LVHTPGPKMP | 22Kan | VLVHTPGPK | 5.28 | NB |
| 320 | QPA**V**LVHTPGPKMPG | 22Kan | VLVHTPGPK | 4.08 | WB |
| 321 | PA**V**LVHTPGPKMPGP | 22Kan | LVHTPGPKM | 4.61 | WB |
| 322 | AVLVHTPGPKMPGPR | 22Kan | LVHTPGPKM | 22.63 | 0 |
|  |  |  |  |  |  |
|  |  |  |  |  |  |
| I318M; I323V | 3Kan | 107:01:00 | Epitope 1A | 2WB --> 2NB | Lack of peptides interactions with BoLA-DRB3 |
| Position | Peptide | Gag consensus/Isolate | Core | Rank % | Binding affinity predictor |
| 317 | K**I**KQPA**I**LVHTPGPK | Consensus | ILVHTPGPK | 23.39 | 0 |
| 318 | **I**KQPA**I**LVHTPGPKM | Consensus | ILVHTPGPK | 10.88 | 0 |
| 319 | KQPA**I**LVHTPGPKMP | Consensus | ILVHTPGPK | 4.83 | WB |
| 320 | QPA**I**LVHTPGPKMPG | Consensus | ILVHTPGPK | 3.80 | WB |
| 321 | PA**I**LVHTPGPKMPGP | Consensus | ILVHTPGPK | 6.86 | 0 |
| 322 | A**I**LVHTPGPKMPGPR | Consensus | LVHTPGPKM | 21.99 | 0 |
|  |  |  |  |  |  |
| 317 | K**M**KQPA**V**LVHTPGPK | 3Kan | VLVHTPGPK | 34.09 | 0 |
| 318 | **M**KQPA**V**LVHTPGPKM | 3Kan | VLVHTPGPK | 20.00 | 0 |
| 319 | KQPA**V**LVHTPGPKMP | 3Kan | VLVHTPGPK | 9.20 | NB |
| 320 | QPA**V**LVHTPGPKMPG | 3Kan | VLVHTPGPK | 7.47 | NB |
| 321 | PA**V**LVHTPGPKMPGP | 3Kan | LVHTPGPKM | 11.08 | 0 |
| 322 | A**V**LVHTPGPKMPGPR | 3Kan | LVHTPGPKM | 21.80 | 0 |
|  |  |  |  |  |  |
|  |  |  |  |  |  |
| I323V | 17USA | 160:01:00 | Epitope 1A | 3SB, 1WB --> 2SB, 2WB | Weakened binding affinity of Epitope to BoLA-DRB3 |
| Position | Peptide | Gag consensus/Isolate | Core | Rank % | Binding affinity predictor |
| 317 | KMKQPA**I**LVHTPGPK | Consensus | ILVHTPGPK | 10.36 | 0 |
| 318 | MKQPA**I**LVHTPGPKM | Consensus | ILVHTPGPK | 2.60 | WB |
| 319 | KQPA**I**LVHTPGPKMP | Consensus | ILVHTPGPK | 0.66 | SB |
| 320 | QPA**I**LVHTPGPKMPG | Consensus | ILVHTPGPK | 0.34 | SB |
| 321 | PA**I**LVHTPGPKMPGP | Consensus | ILVHTPGPK | 0.55 | SB |
| 322 | A**I**LVHTPGPKMPGPR | Consensus | LVHTPGPKM | 5.94 | 0 |
|  |  |  |  |  |  |
|  |  |  |  |  |  |
| 317 | KMKQPA**V**LVHTPGPK | 17USA | VLVHTPGPK | 19.52 | 0 |
| 318 | MKQPA**V**LVHTPGPKM | 17USA | VLVHTPGPK | 4.74 | WB |
| 319 | KQPA**V**LVHTPGPKMP | 17USA | VLVHTPGPK | 1.47 | WB |
| 320 | QPA**V**LVHTPGPKMPG | 17USA | VLVHTPGPK | 0.76 | SB |
| 321 | PA**V**LVHTPGPKMPGP | 17USA | LVHTPGPKM | 0.94 | SB |
| 322 | A**V**LVHTPGPKMPGPR | 17USA | LVHTPGPKM | 8.24 | 0 |
|  |  |  |  |  |  |
|  |  |  |  |  |  |
| I323V | 26USA | 130:01:00 | Epitope 1A | 3WB --> 2WB, 1NB | Weakened binding affinity of Epitope to BoLA-DRB3 |
| Position | Peptide | Gag consensus/Isolate | Core | Rank % | Binding affinity predictor |
| 317 | KMKQPA**I**LVHTPGPK | Consensus | ILVHTPGPK | 17.77 | 0 |
| 318 | MKQPA**I**LVHTPGPKM | Consensus | ILVHTPGPK | 9.54 | 0 |
| 319 | KQPA**I**LVHTPGPKMP | Consensus | ILVHTPGPK | 3.84 | WB |
| 320 | QPA**I**LVHTPGPKMPG | Consensus | ILVHTPGPK | 2.45 | WB |
| 321 | PA**I**LVHTPGPKMPGP | Consensus | LVHTPGPKM | 3.10 | WB |
| 322 | A**I**LVHTPGPKMPGPR | Consensus | LVHTPGPKM | 12.90 | 0 |
|  |  |  |  |  |  |
|  |  |  |  |  |  |
| 317 | KMKQPA**V**LVHTPGPK | 26USA | VLVHTPGPK | 31.29 | 0 |
| 318 | MKQPA**V**LVHTPGPKM | 26USA | VLVHTPGPK | 16.33 | 0 |
| 319 | KQPA**V**LVHTPGPKMP | 26USA | LVHTPGPKM | 6.15 | NB |
| 320 | QPA**V**LVHTPGPKMPG | 26USA | LVHTPGPKM | 3.72 | WB |
| 321 | PA**V**LVHTPGPKMPGP | 26USA | LVHTPGPKM | 3.66 | WB |
| 322 | A**V**LVHTPGPKMPGPR | 26USA | LVHTPGPKM | 14.46 | 0 |
|  |  |  |  |  |  |
|  |  |  |  |  |  |
| I323V | 30USA | 031:01 | Epitope 1A | 5SB, 1WB --> 4SB, 2WB | Weakened binding affinity of Epitope to BoLA-DRB3 |
| I323V | 5USA | 031:01 | Epitope 1A | 5SB, 1WB --> 4SB, 2WB | Weakened binding affinity of Epitope to BoLA-DRB3 |
| Position | Peptide | Gag consensus/Isolate | Core | Rank % | Binding affinity predictor |
| 317 | KMKQPA**I**LVHTPGPK | Consensus | ILVHTPGPK | 0.79 | SB |
| 318 | MKQPA**I**LVHTPGPKM | Consensus | ILVHTPGPK | 0.14 | SB |
| 319 | KQPA**I**LVHTPGPKMP | Consensus | ILVHTPGPK | 0.03 | SB |
| 320 | QPA**I**LVHTPGPKMPG | Consensus | ILVHTPGPK | 0.02 | SB |
| 321 | PA**I**LVHTPGPKMPGP | Consensus | ILVHTPGPK | 0.09 | SB |
| 322 | A**I**LVHTPGPKMPGPR | Consensus | ILVHTPGPK | 1.33 | WB |
|  |  |  |  |  |  |
| 317 | KMKQPA**V**LVHTPGPK | 30USA | VLVHTPGPK | 2.55 | WB |
| 318 | MKQPA**V**LVHTPGPKM | 30USA | VLVHTPGPK | 0.56 | SB |
| 319 | KQPA**V**LVHTPGPKMP | 30USA | VLVHTPGPK | 0.13 | SB |
| 320 | QPA**V**LVHTPGPKMPG | 30USA | VLVHTPGPK | 0.10 | SB |
| 321 | PA**V**LVHTPGPKMPGP | 30USA | VLVHTPGPK | 0.36 | SB |
| 322 | A**V**LVHTPGPKMPGPR | 30USA | VLVHTPGPK | 4.85 | WB |
|  |  |  |  |  |  |
|  |  |  |  |  |  |
| I323V | 30USA | 089:01 | Epitope 1A | 2SB, 2WB --> 3WB, 1NB | Weakened binding affinity of Epitope to BoLA-DRB3 |
| Position | Peptide | Gag consensus/Isolate | Core | Rank % | Binding affinity predictor |
| 317 | KMKQPA**I**LVHTPGPK | Consensus | ILVHTPGPK | 8.86 | 0 |
| 318 | MKQPA**I**LVHTPGPKM | Consensus | ILVHTPGPK | 2.69 | WB |
| 319 | KQPA**I**LVHTPGPKMP | Consensus | ILVHTPGPK | 0.58 | SB |
| 320 | QPA**I**LVHTPGPKMPG | Consensus | ILVHTPGPK | 0.39 | SB |
| 321 | PA**I**LVHTPGPKMPGP | Consensus | ILVHTPGPK | 1.34 | WB |
| 322 | A**I**LVHTPGPKMPGPR | Consensus | LVHTPGPKM | 9.39 | 0 |
|  |  |  |  |  |  |
| 317 | KMKQPA**V**LVHTPGPK | 30USA | VLVHTPGPK | 17.33 | 0 |
| 318 | MKQPA**V**LVHTPGPKM | 30USA | VLVHTPGPK | 7.50 | NB |
| 319 | KQPA**V**LVHTPGPKMP | 30USA | VLVHTPGPK | 2.22 | WB |
| 320 | QPA**V**LVHTPGPKMPG | 30USA | VLVHTPGPK | 1.60 | WB |
| 321 | PA**V**LVHTPGPKMPGP | 30USA | LVHTPGPKM | 2.94 | WB |
| 322 | A**V**LVHTPGPKMPGPR | 30USA | LVHTPGPKM | 12.80 | 0 |
